# Supplementary material for: Cohort profile: Biomarkers related to folate-dependent one-carbon metabolism in colorectal cancer recurrence and survival – the FOCUS Consortium
Source: BMJ Open. 2022 Dec 21;12(12):e062930. doi: 10.1136/bmjopen-2022-062930 (PMC9772678; doi:10.1136/bmjopen-2022-062930)
Supplement: Author's manuscript [file bmjopen-2022-062930.draft_revisions.pdf]

BMJ Open

BMJ Open is committed to open peer review. As part of this commitment we make the peer review history of every article we publish publicly available.

When an article is published we post the peer reviewers' comments and the authors' responses online. We also post the versions of the paper that were used during peer review. These are the versions that the peer review comments apply to.

The versions of the paper that follow are the versions that were submitted during the peer review process. They are not the versions of record or the final published versions. They should not be cited or distributed as the published version of this manuscript.

BMJ Open is an open access journal and the full, final, typeset and author-corrected version of record of the manuscript is available on our site with no access controls, subscription charges or pay-per-view fees (http://bmjopen.bmj.com).

If you have any questions on BMJ Open's open peer review process please email info.bmjopen@bmj.com

BMJ Open

Cohort profile: Biomarkers related to folate-dependent one-carbon metabolism in colorectal cancer recurrence and survival: The FOCUS Consortium

Journal:	*BMJ Open*
Manuscript ID	bmjopen-2022-062930
Article Type:	Cohort profile
Date Submitted by the Author:	15-Mar-2022
Complete List of Authors:	Gigic, Biljana; University Hospital Heidelberg, Department of General, Visceral and Transplantation Surgery<br>van Roekel, Eline ; Maastricht University, Department of Epidemiology<br>Holowatyj, Andreana; Vanderbilt University Medical Center, Medicine; Vanderbilt-Ingram Cancer Center,<br>Brezina, Stefanie; Medical University of Vienna, Institute of Cancer Research, Department of Medicine I<br>Geijsen, Anne; Wageningen University & Research, Division of Human Nutrition and Health<br>Ulvik, Arve; BEVITAL<br>Ose, Jennifer; Huntsman Cancer Institute; University of Utah, Department of Population Health Sciences<br>Koole, Janna L.; Maastricht University, Department of Epidemiology<br>Damerell, Victoria; University Hospital Heidelberg, Department of General, Visceral and Transplantation Surgery<br>Kiblawi, Rama; Huntsman Cancer Institute; University of Utah, Department of Population Health Sciences<br>Gumpenberger, Tanja; Medical University of Vienna, Institute of Cancer Research, Department of Medicine I<br>Lin, Tengda; Huntsman Cancer Institute; University of Utah, Department of Population Health Sciences<br>Kvalheim, Gry; BEVITAL<br>Koelsch, Torsten; Heidelberg University Hospital, Department of General, Visceral and Transplantation Surgery<br>Kok, Dieuwertje; Wageningen University & Research, Division of Human Nutrition and Health<br>van Duijnhoven, Franzel J; Wageningen University & Research, Division of Human Nutrition and Health<br>Bours, Martijn J.; Maastricht University, Department of Epidemiology<br>Baierl, Andreas; University of Vienna, Department of Statistics and Operations Research<br>Li, Christopher I.; Fred Hutchinson Cancer Research Center<br>Grady, William; Fred Hutchinson Cancer Research Center; University of Washington School of Medicine, Department of Medicine<br>Vickers, Kathy; Fred Hutchinson Cancer Research Center<br>Habermann, Nina; European Molecular Biology Laboratory (EMBL), Genome Biology<br>Schneider, Martin; Heidelberg University Hospital, Department of

General, Visceral and Transplantation Surgery
Kampman, Ellen; Wageningen University & Research, Division of Human Nutrition and Health
Ueland, Per Magne; BEVITAL
Ulrich, Alexis; Städtische Kliniken Neuss, Lukaskrankenhaus GmbH, Surgical Department I; University Hospital Heidelberg, Department of General, Visceral and Transplantation Surgery
Weijenberg, Matty ; Maastricht University, Department of Epidemiology
Gsur, Andrea; Medical University of Vienna, Institute of Cancer Research, Department of Medicine I
Ulrich, Cornelia; Huntsman Cancer Institute, Population Sciences; University of Utah, Department of Population Health Sciences

Keywords: Epidemiology < ONCOLOGY, Nutritional support < ONCOLOGY, Gastrointestinal tumours < ONCOLOGY

SCHOLARONE™ Manuscripts

I, the Submitting Author has the right to grant and does grant on behalf of all authors of the Work (as defined in the below author licence), an exclusive licence and/or a non-exclusive licence for contributions from authors who are: i) UK Crown employees; ii) where BMJ has agreed a CC-BY licence shall apply, and/or iii) in accordance with the terms applicable for US Federal Government officers or employees acting as part of their official duties; on a worldwide, perpetual, irrevocable, royalty-free basis to BMJ Publishing Group Ltd ("BMJ") its licensees and where the relevant Journal is co-owned by BMJ to the co-owners of the Journal, to publish the Work in this journal and any other BMJ products and to exploit all rights, as set out in our licence.

The Submitting Author accepts and understands that any supply made under these terms is made by BMJ to the Submitting Author unless you are acting as an employee on behalf of your employer or a postgraduate student of an affiliated institution which is paying any applicable article publishing charge ("APC") for Open Access articles. Where the Submitting Author wishes to make the Work available on an Open Access basis (and intends to pay the relevant APC), the terms of reuse of such Open Access shall be governed by a Creative Commons licence – details of these licences and which Creative Commons licence will apply to this Work are set out in our licence referred to above.

Other than as permitted in any relevant BMJ Author's Self Archiving Policies, I confirm this Work has not been accepted for publication elsewhere, is not being considered for publication elsewhere and does not duplicate material already published. I confirm all authors consent to publication of this Work and authorise the granting of this licence.

For peer review only

Cohort profile: Biomarkers related to folate-dependent one-carbon metabolism in colorectal cancer recurrence and survival: The FOCUS Consortium

Authors:
Biljana Gigic[1], Eline H. van Roekel[2], Andreana N. Holowatyj[3,4,5,6], Stefanie Brezina[7], Anne J.M.R. Geijsen[8], Arve Ulvik[9], Jennifer Ose[3,4], Janna L. Koole[2], Victoria Damerell[1], Rama Kiblawi[3,4], Tanja Gumpenberger[7], Tengda Lin[3,4], Gry Kvalheim[9], Torsten Koelsch[1], Dieuwertje E. Kok[8], Fränzel J.B. van Duijnhoven[8], Martijn J. L. Bours[2], Andreas Baierl[10], Christopher I. Li[11], William M. Grady[11,12,13], Kathy Vickers[11], Nina Habermann[14], Martin Schneider[1], Ellen Kampman[8,16], Per-Magne Ueland[9,16], Alexis B. Ulrich[1,15,16], Matty Weijenberg[2,16], Andrea Gsur[7,16,17] and Cornelia M. Ulrich[3,4,16,17]

Affiliations:
[1] Department of General, Visceral and Transplantation Surgery, Heidelberg University Hospital, Heidelberg, Germany
[2] Department of Epidemiology, Maastricht University, Maastricht, the Netherlands
[3] Huntsman Cancer Institute, Salt Lake City, UT
[4] Department of Population Health Sciences, University of Utah, Salt Lake City, UT
[5] Department of Medicine, Vanderbilt University Medical Center, Nashville, TN
[6] Vanderbilt-Ingram Cancer Center, Nashville, TN
[7] Institute of Cancer Research, Department of Medicine I, Medical University of Vienna, Austria
[8] Division of Human Nutrition and Health, Wageningen University & Research, Wageningen, The Netherlands
[9] BEVITAL, Bergen, Norway
[10] Department of Statistics and Operations Research, University of Vienna, Austria
[11] Public Health Sciences Division, Fred Hutchinson Cancer Research Center, Seattle, Washington
[12] Department of Medicine, University of Washington School of Medicine, Seattle, Washington
[13] Clinical Research Division, Fred Hutchinson Cancer Research Center, Seattle, Washington
[14] Genome Biology, European Molecular Biology Laboratory (EMBL), Heidelberg, Germany
[15] Department of Surgery I, Lukas Hospital Neuss, Neuss, Germany
[16] These authors contributed equally
[17] Corresponding authors

For peer review only

Word Count: 5,995 (including profile in a nutshell)

Tables: 3

Figures: 1

Keywords: cohort, consortium, B-vitamins, folate, folic acid, one-carbon metabolism, dietary supplements, biomarkers, treatment toxicity, colorectal cancer, recurrence, survival

For peer review only - http://bmjopen.bmj.com/site/about/guidelines.xhtml

Abstract

Purpose: The overarching goal of the FOCUS (Biomarkers related to folate-dependent one-carbon metabolism in colorectal cancer recurrence and survival) Consortium is to unravel the effect of folate and folate-mediated one-carbon metabolism (FOCM) biomarkers on colorectal cancer (CRC) prognosis to provide clinically relevant advice on folate intake to cancer patients and define future tertiary prevention strategies.

Participants: The FOCUS Consortium is an international, prospective cohort of 2,401 women and men above 18 years of age who were diagnosed with a primary invasive non-metastatic (stage I-III) CRC. The Consortium comprises patients from Austria, two sites from the Netherlands, Germany and two sites from the USA. Patients are recruited after CRC diagnosis and followed at six and twelve months after enrolment. At each time point, sociodemographic data, data on health behavior, and clinical data are collected, blood samples are drawn.

Findings to date: An increased risk of cancer recurrences was observed among patients with higher compared to lower circulating folic acid concentrations. Furthermore, specific folate species within the FOCM pathway were associated with both inflammation and angiogenesis pathways among CRC patients. In addition, higher vitamin B_6 status was associated with better quality of life at six months post treatment.

Future plans: Better insights into the research on associations between folate and FOCM biomarkers and clinical outcomes in CRC patients will facilitate the development of guidelines regarding folate intake in order to provide clinically relevant advice to cancer patients, health professionals involved in patient care, and ultimately further tertiary prevention strategies in the future.

Strengths and limitations of this study

- FOCUS is the largest consortium to date addressing the research question of folate and FOCM biomarkers in relation to survival, recurrence, treatment toxicity, and health-related quality of life outcomes in CRC patients
- The cohorts included in the FOCUS Consortium are designed to enable future pooling of data using harmonized and standardized methods to collect data and biospecimens
- The pooled sample size provides sufficient power to investigate subgroup analyses across CRC patients
- Study time point definitions differ between some of the cohorts and have to be adapted for specific projects
- A selection bias for follow-up can arise because it is possible that patients who experience more severe toxicities, worse clinical outcome or health-related quality of life are underrepresented among those completing follow-ups

For peer review only - http://bmjopen.bmj.com/site/about/guidelines.xhtml

Why was the FOCUS Consortium set up?

Among men and women world-wide, colorectal cancer (CRC) accounts for nearly 10% of all incident cancer cases.[1] In Europe, 5-year survival for CRC patients is approximately 55%, with substantial differences by stage.[2 3] The number of CRC patients continues to increase due to implementation of improved screening strategies and/or enhanced treatment modalities.[4] Many cancer patients seek information on what they can do themselves to improve survival - for instance by improving their dietary habits and other lifestyle factors.[5] However, there may be behavioural aspects among individuals diagnosed with CRC, which may be harmful in some cases, such as the use of high-dose nutritional supplements containing synthetic folate. In general, knowledge on short and long-term effects is insufficient to make sound recommendations on use of dietary supplements, in particular folate, to cancer survivors, even though dietary supplements are used by 20%-85% of patients with cancer[6-8], highlighting the importance of thorough evaluation of potential benefits and harms and to support development of evidence-based recommendations on use of dietary supplements to cancer patients.

Folic acid is the synthetic form of the B-vitamin folate and is often used in dietary supplements or fortified foods. Folate and folic acid play an important role in one-carbon metabolism, which is a complex series of biochemical reactions essential in nucleotide synthesis, methylation reactions and amino acid homeostasis (REF PMID: 27641100). One-carbon metabolism refers to a complex network of biochemical reactions linked to nucleotide synthesis and provides methyl groups for DNA, RNA or protein methylation. Thus, one-carbon metabolism is directly controlling processes determining DNA synthesis and integrity, both processes known to be linked to tumor growth.[9] To what extent folic acid supplement use and biomarkers of folate-mediated one-carbon metabolism (FOCM) impact cancer survival and treatment efficacy and toxicity still needs to be clarified.[9 10] Folate and FOCM biomarker deficiencies may increase cancer risk, but high levels, especially of synthetic folic acid, may also be driving factors in carcinogenesis.[6 11] An increasing body of evidence suggests that folate plays a dual role in carcinogenesis, involving both the prevention of early lesions and potential harm once pre-neoplastic or neoplastic lesions have developed.[6 10 12-18]

The overarching goal of the FOCUS (biomarkers related to FOlate-dependent one-carbon metabolism in Colorectal cancer recUrrence and Survival) Consortium is to study associations between folate and FOCM biomarkers and recurrence and survival in CRC patients. Better insights into these associations will facilitate the development of guidelines

For peer review only - http://bmjopen.bmj.com/site/about/guidelines.xhtml

regarding folate intake in order to provide clinically relevant advice to cancer patients, health professionals involved in patient care, and ultimately further tertiary prevention strategies in the future. The FOCUS Consortium is a large-scale international consortium with CRC patients from six prospective cohort studies. The primary objectives of the FOCUS Consortium are: (i) to determine possible associations of folate and other FOCM biomarkers at diagnosis with recurrence or survival in non-metastatic (stage I-III) CRC; (ii) to elucidate whether biomarkers related to FOCM are associated with dietary and supplemental intake of these respective nutrients; (iii) to explore whether FOCM biomarkers are associated with treatment toxicity in CRC patients undergoing chemotherapy; and (iv) to collect comprehensive data of patient characteristics at baseline and follow-up including biomarkers of FOCM to establish an unique resource for future scientific research. The FOCUS Consortium is funded by the European Research Area Network (ERA-NET) on Translational Cancer Research (TRANSCAN).

This joint research may lead to a better understanding of the role of folate- and FOCM-related mechanisms in the prognosis of CRC, which will be critical for the development of guidelines regarding folate intake among CRC patients.

Who is in the FOCUS Consortium?

The FOCUS Consortium is an international, prospective consortium including six cohort studies recruiting women and men age 18 years and older diagnosed with a primary CRC. The FOCUS Consortium is comprised of patients from the ColoCare Study at the University of Heidelberg, Germany (n=298, 12.3%), the ColoCare Study at the Huntsman Cancer Institute in Salt Lake City, Utah, USA (n=80, 3.3%), the ColoCare Study at the Fred Hutchinson Cancer Research Center in Seattle, Washington, USA (n=157, 6.3%), the COLON Study (COlorectal cancer: Longitudinal, Observational study on Nutritional and lifestyle factors that may influence colorectal tumor recurrence, survival and quality of life) at Wageningen University & Research in the Netherlands (n=1,365, 56.1%), the CORSA Study (Colorectal Cancer Study of Austria) at the Medical University of Vienna in Austria (n=218, 9.0%), and the EnCoRe Study (Energy for life after ColoRectal cancer) at Maastricht University Medical Center+ in the Netherlands (n=317, 13.0%).

In total, n=2,435 CRC patients were considered for the FOCUS Consortium, of which n=34 patients were excluded due to tumor staging being either 0 or IV leading to a total number of n=2,401 stage I-III CRC patients included in further analyses.

For peer review only - http://bmjopen.bmj.com/site/about/guidelines.xhtml

Patients were recruited after CRC diagnosis and repeated study measurements were conducted at time of recruitment, and at six and twelve months thereafter. At each study time point, sociodemographic data, data on lifestyle factors (e.g., diet, supplement use, physical activity), and clinical data (e.g., tumor site and stage, cancer treatment, treatment-induced toxicities, recurrence, survival) were collected, and blood samples were drawn (**Figure 1**). All individuals signed informed consent and the Institutional Review Board at each site approved the corresponding study. Below, a more specific description of each included study is provided.

<u>The ColoCare Study</u>

The ColoCare Study (ClinicalTrials.gov identifier: NCT02328677) is an ongoing international, multi-center prospective cohort study among women and men newly diagnosed with a primary invasive CRC, with the goal to investigate predictors of cancer recurrence, survival, treatment toxicities and health-related quality of life.[4 19] Three ColoCare Consortium sites participate in the FOCUS Consortium: the Fred Hutchinson Cancer Research Center in Seattle (Washington, USA), and the University Hospital Heidelberg, Heidelberg (Germany) as well as the Huntsman Cancer Institute in Salt Lake City (Utah, USA). Patients are enrolled prior to undergoing CRC surgery according to the following inclusion criteria: individuals who are 18 years of age and older, newly diagnosed (i.e., non-recurrent) with invasive CRC. Blood draws and other biospecimens are obtained prior to surgery and at regular intervals (e.g., six, twelve, twenty-four months). Questionnaires are administered to assess lifestyle behavior, health-related quality of life, and clinical outcomes such as CRC recurrence, treatment, and treatment symptoms at each study time point. Clinical data are obtained through reviews of patient medical records, pathology and imaging reports. Vital status is obtained through medical records, routine follow-up mailings, periodic requests for outside medical records, and state or national cancer and death registries. The Heidelberg ColoCare Study was approved by the Ethics Committee of the Medical Faculty of Heidelberg (approval no. S-310/2001 and S-134/2016). The ColoCare Study at the Huntsman Cancer Institute and the Fred Hutchinson Cancer Research Center were approved by the respective IRBs (#77147 and #6407).

<u>The COLON Study</u>

The COLON Study (ClinicalTrials.gov identifier: NCT03191110) started in 2010 and is an ongoing, multicenter prospective cohort study specifically designed to assess associations

For peer review only

For peer review only - http://bmjopen.bmj.com/site/about/guidelines.xhtml

between nutrition, lifestyle, and dietary supplement use with quality of life, CRC recurrence and survival among CRC patients (stages I-IV).[20] Persons with a history of CRC or (partial) bowel resection, chronic inflammatory bowel disease, or a known hereditary CRC syndrome are excluded from the study. Patients are recruited from eleven regional and academic hospitals prior to surgery. Individuals donate blood samples and provide information on diet, lifestyle, and dietary supplement use at CRC diagnosis, i.e. baseline, and at six, twelve (chemotherapy patients only), twenty-four and sixty months after diagnosis. Clinical data are collected through review of medical records (treatment-induced toxicity) or through linkage with the Dutch ColoRectal Audit (DCRA). Mortality data (i.e. death or alive and date of death) are retrieved from the Municipal Personal Records Database. Recurrence data have been retrieved in collaboration with the Netherlands Cancer Registry. Ethical approval for the study was granted by the Committee on Research involving Human Subjects, region Arnhem-Nijmegen under file number 2009/349.

<u>The CORSA Study</u>

CORSA is an ongoing case-control study of women and men recruiting CRC patients, patients with high and low risk adenomas and population-based colonoscopy negative controls, with an age range between 30 and 90 years. Since 2003, more than 13,500 participants have been recruited across nine sites in Austria. The multicentre recruitment within CORSA follows standardized protocols resulting in consistent data from all recruitment sites. These sites include the Medical University of Vienna (Department of Surgery), two hospitals in Vienna (Clinic Favoriten and Clinic Landstraße), and the Hospital Wiener Neustadt in Lower Austria. Furthermore, the recruitment for CORSA was performed in four hospitals in the federal state Burgenland within the population-based screening program "Burgenland PREvention Trial of colorectal cancer DIsease with ImmunologiCal Testing" (B-PREDICT). B-PREDICT is a two-stage screening project where more than 150,000 inhabitants of Burgenland aged between 40 and 80 are invited annually to participate in this program using a Fecal Immunochemical Test (FIT) as an initial screening. FIT-positive (≥10 μg hemoglobin / g feces) tested individuals are offered a complete colonoscopy and are asked to participate in CORSA. Biospecimen are collected at each site using harmonized protocols. CORSA participants provide a basic CORSA questionnaire assessing data on body mass index (BMI), smoking history, alcohol consumption, education level, family status, profession, basic dietary habits, and diabetes. Clinical data are abstracted from medical records and processed in a structured database following standardized documentation

For peer review only

For peer review only - http://bmjopen.bmj.com/site/about/guidelines.xhtml

guidelines and according to the General Data Protection Regulation (GDPR). All subjects gave written informed consent and the study was approved by the institutional review boards: 'Ethikkommission Burgenland' (33/2010), by the ethical review committee of the Medical University of Vienna (1160/2016) and by the "Ethikkommission der Stadt Wien" (EK 06-150VK).

The EnCoRe Study

The EnCoRe Study is a prospective cohort study initiated in 2012 at the Maastricht University Medical Center+ in the Netherlands that focuses on the importance of lifestyle factors for quality of life, recurrence and survival of CRC patients.[21] The EnCoRe Study is registered in the Netherlands Trial Registry for experimental and observational studies (www.trialregister.nl, registration number 7099). Stage I-III CRC patients are enrolled at diagnosis at three hospitals in the South-Eastern region of the Netherlands and followed up until five years after completion of treatment with repeated measurements at diagnosis (pre-treatment), and at six weeks and six, twelve, twenty-four, and sixty months after the end of the initial anti-cancer treatment (i.e., surgery, chemotherapy, radiotherapy). Patients with stage IV CRC, an inability to understand the Dutch language in speech or writing, with comorbidities obstructing successful participation (e.g., Alzheimer disease), or with severe visibility or hearing disorders are excluded from the study. Repeated home visits by trained dieticians are conducted and data are collected amongst others on sociodemographic factors, quality of life, functioning, physical activity, comorbidity, dietary intake, supplement use, and anthropometry. In addition, clinical data are collected from hospital records and blood samples are drawn at all time points. Recurrence data have been retrieved in collaboration with the Netherlands Cancer Registry. Mortality data (i.e. death or alive and date of death) are retrieved from the Municipal Personal Records Database. The EnCoRe Study was approved by the Medical Ethics Committee of the Maastricht University Hospital and Maastricht University (METC 11-3-075).

The FOCUS Consortium

Sociodemographic, clinical and lifestyle characteristics of the 2,401 participants in the FOCUS Consortium are presented in **Table 1**. The mean age at CRC diagnosis was 65.4 years (standard deviation (SD): 10.2; range: 22 to 93 years), and the majority of participants were male (64.0%). The mean BMI was 27.0 kg/m² (SD: 4.6).

For peer review only - http://bmjopen.bmj.com/site/about/guidelines.xhtml

Table 1: Sociodemographic, clinical, and lifestyle factors of eligible FOCUS Consortium participants at baseline (n=2,401)

	Overall	ColoCare HD	ColoCare FHCRC	ColoCare HCI	COLON	CORSA	EnCoRe
n (%)	2,401 (100)	298 (12.4)	150 (6.3)	80 (3.3)	1,338 (55.7)	218 (9.1)	317 (13.2)
**Age**							
**Mean** y ± SD	65.4 ± 10.2	63.7 ± 12.2	58.3 ± 12.8	61.3 ± 11.2	66.1 ± 8.7	67.3 ± 12.0	66.9 ± 9.3
**Sex**							
**Female** n (%)	864 (36.0)	100 (33.6)	67 (44.7)	32 (40.0)	486 (36.3)	76 (34.9)	103 (32.5)
**Male** n (%)	1,537 (64.0)	198 (66.4)	83 (55.3)	48 (60.0)	852 (63.7)	142 (65.1)	214 (67.5)
**Education**							
**Low** n (%)	861 (35.9)	123 (41.3)	18 (12.0)	17 (21.3)	543 (40.6)	73 (33.5)	87 (27.4)
**Intermediate** n (%)	694 (28.9)	57 (19.1)	71 (47.3)	40 (50.0)	332 (24.8)	73 (33.5)	121 (38.2)
**High** n (%)	658 (27.4)	88 (29.5)	28 (18.7)	8 (10.0)	417 (31.7)	14 (6.4)	103 (32.5)
**Unknown** n (%)	188 (7.8)	30 (10.1)	33 (22.0)	15 (18.7)	46 (3.4)	58 (26.6)	6 (1.9)
**Marital status**							
**Unmarried** n (%)	122 (5.1)	19 (6.4)	15 (10.0)	2 (2.5)	60 (4.5)	10 (4.6)	16 (5.1)
**Married** n (%)	1,738 (72.4)	193 (64.8)	71 (47.3)	50 (62.5)	1,071 (80.0)	113 (51.8)	240 (75.7)
**Divorced/Separated** n (%)	138 (5.7)	24 (8.0)	11 (7.3)	10 (12.5)	64 (4.8)	14 (6.4)	15 (4.7)
**Widowed** n (%)	194 (8.1)	31 (10.4)	7 (4.7)	1 (1.3)	90 (6.7)	26 (11.9)	39 (12.3)
**Living community** n (%)	20 (0.8)	0	13 (8.7)	2 (2.5)	4 (0.3)	1 (0.5)	0
**Unknown** n (%)	189 (8.9)	31 (10.4)	33 (22.0)	15 (18.7)	49 (3.7)	54 (24.8)	7 (2.2)
**Smoking status**							
**Current** n (%)	290 (12.1)	53 (17.8)	8 (5.3)	6 (7.5)	143 (10.7)	38 (17.4)	42 (13.3)
**Former** n (%)	1,219 (50.8)	132 (44.3)	53 (35.32)	21 (26.3)	767 (57.3)	76 (34.9)	170 (53.6)
**Never** n (%)	767 (31.9)	90 (30.2)	56 (37.3)	39 (48.7)	386 (28.9)	97 (44.5)	99 (31.2)
**Unknown** n (%)	125 (5.2)	23 (7.7)	33 (22.0)	14 (17.5)	42 (3.1)	7 (3.2)	6 (1.9)
**Alcohol intake**							
**Mean** g/day ± SD	13.6 ± 17.1	16.1 ± 19.2	8.8 ± 18.5	2.3 ± 10.2	14.0 ± 17.0		12.9 ± 16.5
**Unknown** n (%)	633 (26.4)	208 (69.8)	81 (54.0)	60 (75.0)	54 (4.1)	218 (100)	12 (3.8)
**BMI at diagnosis**							
**Mean** kg/m^2 ± SD	27.1 ± 4.6	26.6 ± 4.1	28.7 ± 7.3	29.4 ± 7.5	26.6 ± 4.0	27.7 ± 4.4	28.3 ± 4.6
**Unknown** n (%)	42 (1.7)	2 (0.7)	0	14 (17.5)	10 (0.8)	14 (6.4)	2 (0.6)
	**Overall**	**ColoCare HD**	**ColoCare FHCRC**	**ColoCare HCI**	**COLON**	**CORSA**	**EnCoRe**

For peer review only - http://bmjopen.bmj.com/site/about/guidelines.xhtml

**Tumor site**							
**Colon** n (%)	1,450 (60.4)	140 (57.0)	75 (50.0)	45 (56.3)	867 (64.8)	131 (60.1)	192 (60.6)
**Rectum** n (%)	862 (35.9)	143 (48.0)	60 (40.0)	22 (27.5)	434 (32.4)	78 (35.8)	125 (39.4)
**Rectosigmoid** n (%)	42 (1.7)	15 (5.0)	15 (10.0)	7 (8.7)	0	5 (2.3)	0
**Unknown** n (%)	47 (2.0)	0	0	6 (7.5)	37 (2.8)	4 (1.8)	0
**Tumor stage**							
**I** n (%)	629 (26.2)	76 (25.5)	35 (23.3)	21 (26.3)	326 (24.4)	82 (37.6)	89 (28.1)
**II** n (%)	694 (28.9)	114 (38.3)	49 (32.7)	23 (28.7)	386 (28.9)	52 (23.9)	70 (22.1)
**III** n (%)	973 (40.5)	108 (36.2)	66 (44.0)	36 (45.0)	564 (42.1)	52 (23.9)	147 (46.4)
**Unspecified*** n (%)	16 (0.7)	0	0	0	4 (0.3)	12 (5.5)	0
**Unknown** n (%)	89 (3.7)	0	0	0	58 (4.3)	20 (9.2)	11 (3.5)
**Treatment**							
**Neoadjuvant chemotherapy** n (%)	331 (13.8)	55 (18.5)	47 (31.3)	15 (18.8)	138 (10.3)	14 (6.4)	62 (19.6)
**Unknown** n (%)	58 (2.4)	2 (0.7)	1 (0.7)	10 (12.5)	38 (2.8)	7 (3.2)	0
**Neoadjuvant radiotherapy** n (%)	523 (21.8)	72 (24.2)	48 (32.0)	13 (16.3)	290 (21.7)	14 (6.4)	86 (27.1)
**Unknown** n (%)	58 (2.4)	2 (0.7)	1 (0.7)	10 (12.5)	38 (2.8)	7 (3.2)	0
**Adjuvant chemotherapy** n (%)	682 (28.4)	100 (33.6)	80 (53.3)	32 (40.0)	315 (23.8)	56 (25.7)	99 (31.2)
**Unknown** n (%)	121 (5.0)	17 (5.7)	3 (2.0)	28 (35.0)	63 (4.7)	9 (4.1)	1 (0.3)
**Adjuvant radiotherapy** n (%)	227 (9.5)	188 (63.1)	7 (4.7)	1 (1.3)	16 (1.2)	13 (6.0)	2 (0.6)
**Unknown** n (%)	164 (6.8)	17 (5.7)	3 (2.0)	28 (35.0)	106 (7.9)	9 (4.1)	1 (0.3)
**Surgery** n (%)	2,291 (95.4)	298 (100.0)	149 (99.3)	71 (88.7)	1,290 (96.4)	195 (89.5)	288 (90.9)
**Unknown** n (%)	44 (1.8)	0	0	3 (3.8)	39 (2.9)	1 (0.5)	1 (0.3)
**Folic acid supplementation**							
**Yes** n (%)	405 (16.9)	4 (1.3)	9 (5.9)	2 (2.5)	325 (24.3)		65 (20.5)
**Unknown** n (%)	383 (15.9)	27 (9.1)	86 (56.6)	14 (17.5)	38 (2.8)	218 (100)	0
**Vitamin B_{12} supplementation**							
**Yes** n (%)	430 (17.9)	10 (3.4)	8 (5.3)	14 (17.5)	336 (25.1)		62 (19.6)
**Unknown** n (%)	384 (16.0)	27 (9.1)	86 (56.6)	14 (17.5)	39 (2.9)	218 (100)	0
**Vitamin B_6 supplementation**							
**Yes** n (%)	77 (3.2)	8 (2.7)	4 (2.6)	4 (5.0)			61 (19.2)
**Unknown** n (%)	1,684 (70.1)	27 (9.1)	86 (56.6)	14 (17.5)	1,339 (100)	218 (100)	0

For peer review only - http://bmjopen.bmj.com/site/about/guidelines.xhtml

	Overall	ColoCare HD	ColoCare FHCRC	ColoCare HCI	COLON	CORSA	EnCoRe
**Vitamin B_2 supplementation**							
**Yes** n (%)	62 (2.6)	0	2 (1.3)	0			60 (18.9)
**Unknown** n (%)	1,684 (70.1)	27 (9.1)	86 (56.6)	14 (17.5)	1,339 (100)	218 (100)	0
**Dietary intake of folate equivalents**							
**Mean** µg/day ± SD	227.1 ± 94.3	115.1 ± 38.1	149.6 ± 97.5	116.2 ± 112.8	229.0 ± 88.7		276.3 ± 82.2
**Unknown** n (%)	638 (26.5)	208 (69.8)	83 (54.6)	60 (75.0)	57 (4.3)	218 (100)	12 (3.8)
**Dietary intake of vitamin B_{12}**							
**Mean** µg/day ± SD	4.6 ± 2.4	6.2 ± 2.9	7.1 ± 5.8	4.2 ± 2.2	4.3 ± 2.1		4.6 ± 2.0
**Unknown** n (%)	638 (26.5)	208 (69.8)	83 (54.6)	60 (75.0)	57 (4.3)	218 (100)	12 (3.8)
**Dietary intake of vitamin B_6**							
**Mean** mg/day ± SD	1.5 ± 0.5	1.7 ± 0.5	2.0 ± 0.7	1.5 ± 0.7	1.5 ± 0.5		1.8 ± 0.5
**Unknown** n (%)	638 (26.5)	208 (69.8)	83 (54.6)	60 (75.0)	57 (4.3)	218 (100)	12 (3.8)
**Dietary intake of vitamin B_2**							
**Mean** mg/day ± SD	1.4 ± 0.5	1.5 ± 0.5	2.2 ± 1.0	1.6 ± 0.8	1.3 ± 0.4		1.4 ± 0.5
**Unknown** n (%)	638 (26.5)	208 (69.8)	83 (54.6)	60 (75.0)	57 (4.3)	218 (100)	12 (3.8)
**Total energy intake**							
**Mean** kcal/day ± SD	1,941.8 ± 593.6	2,330.9 ± 714.0	1835.0 ± 775.7	1,398.5 ± 597.8	1,856.9 ± 517.2		2,243.7 ± 651.0
**Unknown** n (%)	638 (26.5)	208 (69.8)	83 (54.6)	60 (75.0)	57 (4.3)	218 (100)	12 (3.8)
**Adherence to physical activity guidelines****							
**Yes** n (%)	1,310 (54.5)	84 (28.2)	35 (23.0)	20 (25.0)	941 (70.3)		230 (72.6)
**Unknown** n (%)	370 (15.4)	33 (11.1)	53 (34.9)	15 (18.7)	45 (3.4)	218 (100)	6 (1.9)

*Distinguishing between stage I and II or II and III not possible

**Self-reported engagement in at least 150 minutes per week of moderate-to-vigorous physical activity

For peer review only - http://bmjopen.bmj.com/site/about/guidelines.xhtml

In terms of education status, 35.9% of the patients reported lower education, 28.8% reported intermediate education and 27.3% high education. Most of the patients were married or part of a living community (73.1%). About half of the overall cohort reported to be a former smoker (50.8%), 31.7% were never smokers, and 12.1% were current smokers (5.4% were unknown). Median alcohol intake was 7.9 g/day. In total, 16.9% of the patients reported regular dietary supplementation (i.e., at least once per week during the last four weeks) of folic acid. Over half of all participants (54.6%) reported adherence to the physical activity guidelines of at least 150 minutes per week of moderate-to-vigorous physical activity.

Regarding clinical characteristics, 57.0% of participants were diagnosed with colon cancer, 33.9% with rectal cancer, and 1.1% with rectosigmoid cancer (8.1% were of unknown tumor subsite). In total, 26.0% were diagnosed with stage I, 28.7% with stage II, and 40.2% with stage III CRC. Approximately 1% of participants were classified with an unspecified cancer stage, as distinction between stage I and II or II and III was not possible, and for 4.4% of the total population the cancer stage was unknown. In total, 89.3% of patients underwent surgery, whereas neoadjuvant chemotherapy was administered to 10.6% of patients and 26.8% received adjuvant chemotherapy. Neoadjuvant radiotherapy was administered to 20.7% of patients and adjuvant radiotherapy in 9.4% of the population.

Patient and public involvement

Patients and the public were not involved in the design, conduct, reporting or dissemination plans of this research.

How often have study participants been followed up?

At each study site, collection of biospecimen, clinical, demographic, questionnaire and anthropometric data occurred at baseline, and at six and twelve months following recruitment. Baseline measurements in CORSA, COLON and EnCoRe have been performed at diagnosis (preferably prior to any cancer treatment) while in the ColoCare Study such measurements have been done prior to surgery (i.e. neoadjuvant treatment might have been applied prior to baseline blood and data collection).

Blood samples at baseline were collected from n=2,132 (88.8%) study participants. N=1,537 (64.0%) participants donated blood at the 6 month and n=614 (25.6%) at the 12 month follow-up time point. Additionally, n=251 (10.5%) EnCoRe patients provided blood

For peer review only

For peer review only - http://bmjopen.bmj.com/site/about/guidelines.xhtml

samples at 6 weeks post-treatment, a subset specifically of interest at the Maastricht study site.

What has been measured/recorded within the FOCUS Consortium?

Harmonized baseline and follow-up time points are summarized in **Table 2** and are briefly described below.

Table 2: Variables available at baseline, and at 6 and 12 month follow-up within the FOCUS Consortium.

Category	Variables	Baseline	6m	12m
**Demographics**	Age	x	x	x
	Gender	x		
	Highest education	x		
	Social status	x		
	Height	x		
	Weight	x	x	x
	BMI	x	x	x
	Smoking status	x	x	x
	Smoking duration	x	x	x
	Smoking pack years	x	x	x
	Menopausal status	x		
	Postmenopausal hormone use	x		
	Race	x		
**Cancer characteristics**	Cancer site	x		
	Cancer stage	x		
	TNM classification	x		
**Treatment**	Preoperative chemotherapy	x		
	Preoperative radiotherapy	x		
	Surgery	x	x	
	Postoperative chemotherapy		x	x
	Postoperative radiotherapy		x	x
**Supplement intake**	Folic acid supplements	x	x	x
	Vitamin B_2 supplements	x	x	x
	Vitamin B_6 supplements	x	x	x
	Vitamin B_{12} supplements	x	x	x
	Vitamin A supplements	x	x	x
	Vitamin C supplements	x	x	x
	Vitamin D supplements	x	x	x

For peer review only

**Supplement intake**	Vitamin E supplements	x	x	x
	Calcium supplements	x	x	x
	Magnesium supplements	x	x	x
	Iron supplements	x	x	x
	Multivitamins	x	x	x
**Dietary nutrients**	Folate equivalents	x	x	x
	Vitamin B_2	x	x	x
	Vitamin B_6	x	x	x
	Vitamin B_{12}	x	x	x
	Vitamin A	x	x	x
	Vitamin C	x	x	x
	Vitamin D	x	x	x
	Vitamin E	x	x	x
	Total protein	x	x	x
	Total fat	x	x	x
	Total carbohydrate	x	x	x
	Fibre	x	x	x
	Saturated fatty acids	x	x	x
	Monounsaturated fatty acids	x	x	x
	Polyunsaturated fatty acids	x	x	x
	Alcohol	x	x	x
	Total energy	x	x	x
**Physical activity**	Light physical activity	x	x	x
	Moderate physical activity	x	x	x
	Vigorous physical activity	x	x	x
	Adherence to physical activity guidelines	x	x	x
**Medical history**	Diabetes mellitus	x	x	x
	Asthma, Chronical Bronchitis, COPD, Emphysema	x		
	Heart attack, Heart Failure	x		
	Hypertension	x		
	Stroke	x		
	Ulcer of Stomach or Duodenum	x		
	Hypothyroidism/Hyperthyroidism	x		
	Systemic Lupus Erythematosus	x		
	Inflammatory Bowel Disease (Crohn's disease, ulcerative colitis)	x		
	Familial Adenomatous Polyposis	x		

For peer review only - http://bmjopen.bmj.com/site/about/guidelines.xhtml

**Medical history**	Lynch Syndrome (hereditary nonpolyposis colorectal cancer)	x		
**Regular medication use**	Aspirin	x	x	x
	NSAID	x	x	x
	Ibuprofen	x	x	x
	Naproxen	x	x	x
	Celecoxib/Etoricoxib	x	x	x
**Health-related quality of life**	EORTC QLQ-C30	x	x	x
	EORTC QLQ-CR29	x	x	x
	EORTC QLQ-CIPN20	x	x	X
**General information**	Date of questionnaire completion	x	x	x
	Date of blood collection	x	x	x
	Freeze-thaw cycles of blood samples	x	x	x
	Hemolysis	x	x	x
	Time between blood draw and processing/storage	x	x	x

NSAID=non-steroidal anti-inflammatory drug; EORTC=European Organization for Research and Treatment of Cancer; QLQ=quality of life questionnaire; C30=core 30-items; CR29=colon/rectum 29-items; CIPN20=chemotherapy-induced polyneuropathy 20-items.

Lifestyle and demographic data

ColoCare questionnaires and standardized Food Frequency Questionnaires (FFQ) are used to assess intake of dietary supplements and medication, smoking, dietary intake and other health behaviors at each study time point. In COLON, patients provide information on diet, lifestyle, and supplement use via COLON questionnaires and FFQs. Patients from the CORSA Study are requested to complete a questionnaire assessing anthropometric and demographic factors. Patients enrolled in EnCoRe receive repeated home visits by trained dieticians, where extensive measurements are performed that include assessment of demographic data, physical activity (both questionnaire- and accelerometry-based) and smoking behavior (questionnaire data), dietary intake (FFQs at diagnosis and 7-day food diaries at follow-up measurements), supplement use (registered by dieticians), and anthropometry measurements.

Clinical data and outcomes - Medical chart abstraction

All cancers and medical diagnoses are classified according to ICD-10 (International Classification of Diseases, Tenth Revision) codes. Details on CRC treatment, including type

For peer review only

of treatment regimens and treatment toxicity, are abstracted via medical record for all cohorts. Detailed information on the primary outcomes of interest, CRC recurrence and survival, are ascertained through reviews of medical records, pathology reports, and imaging reports documenting the diagnosis of a recurrence. Data on recurrence and vital status is supplemented from the clinical cancer registries and survival data is verified by the inhabitant registries that exist at all study sites. For COLON and EnCoRe, all data on recurrence are retrieved in collaboration with the Netherlands Cancer Registry. Moreover, survival data for CORSA participants was obtained by the Main Association of Austrian Social Insurance Carriers as well as from Statistics Austria.

<u>Patient-reported outcomes</u>

Health-related quality of life is assessed by the validated and widely used cancer-specific 30-item core questionnaire QLQ-C30 and the 29-item CRC module QLQ-CR29, developed by the European Organization for Research and Treatment of Cancer (EORTC).[22] Chemotherapy-induced peripheral neuropathy (CIPN), which is a common toxicity in CRC patients, is measured by the EORTC QLQ-CIPN20.[23] Patient-reported outcomes are available for ColoCare Heidelberg, COLON and EnCoRe.

<u>Biomarkers of FOCM</u>

Blood is processed in identical settings across all study centers. Plasma (CORSA, COLON and EnCoRe) and serum (for the three ColoCare Study sites) samples were collected and immediately centrifuged, aliquoted, and stored at -80°C.

All biological analyses were performed at BEVITAL AS (Bergen, Norway, http://www.bevital.no), which carried out metabolic profiling of biomarkers allocated to seven complementary analytical platforms. Apart from analyses of microbiological active folate[24] and vitamin B_{12}[25]), all analyses were based on mass spectrometry. Circulating folate, separate folate species, 5-methyltetrahydrofolate (the main form in plasma), folic acid (folate in supplements), 4a-OH 5-methyltetrahydrofolate (the primary oxidation form of circulating folate), folate catabolites (p-aminobenzoylglutamate and acetyl p-aminobenzoylglutamate)[26], B_6, B_1 and B_3 vitamins, kynurenines, cotinine, trans-3'-hydroxycotinine, trigonelline[27], choline and its metabolites, creatinine, methylhistidines, arginine and methylated arginines[28] were analyzed by liquid chromatography-tandem mass spectroscopy, whereas other amino acids, in addition to total homocysteine, total cysteine and methylmalonic acid were analyzed

For peer review only

327 by gass chromatography-tandem mass spectroscopy.[29] C-reactive protein (hsCRP) and
328 cystatin C and its variants were measured by MALDI-TOF mass spectroscopy.[30]

329 A comprehensive overview of the panel of biomarkers measured in blood samples of
330 patients enrolled in the FOCUS Consortium is provided in **Table 3**.

Table 3: Measured metabolites and biomarkers within the FOCUS Consortium

**Folate and one-carbon metabolites**	**Abbreviation**	**Description**
**Anthranilic acid**	AA	Tryptophan metabolite
**Asymmetric dimethylarginine**	ADMA	Inhibitor of nitric oxide synthase
**α-Ketoglutaric acid**	aKG	Keto acid of the Krebs cycle
**Alanine**	Ala	Amino acid
**Acetamidobenzoylglutamate**	apABG	Folate catabolite
**Arginine**	Arg	Amino acid
**Asparagine**	Asn	Amino acid
**Aspartic acid**	Asp	Amino acid
**Betaine**	Betaine	Methyl donor
**Choline**	Choline	Methyl donor
**C-reactive protein**	hsCRP	Inflammatory marker
**Cystatin C-desS**	CnCds	Cystatin C isoform
**Cystatin C-desSSP**	CnDcssp	Cystatin C isoform
**Cystatin C**	CnCn	Marker of kidney function
**3Pro-OH cystatin C**	CnCo	Cystatin C isoform
**3Pro-OH cystatin C-desS**	CnCods	Cystatin C isoform
**Total concentration of detected cystatin C isoforms**	CnCt	Marker of kidney function
**Cobalamin**	B_{12}	Vitamin B_{12}
**Cotinine**	Cot	Nicotine metabolite
**Creatinine**	Creat	Marker of kidney function
**C-reactive protein**	CRP	Inflammation
**Cystathionine**	Cysta	Thioether, transsulfuration intermediate
**Dimethylglycine**	DMG	Betaine metabolite
**Folic acid**	B_9	Synthetic form of folate
**Flavin mononucleotide**	FMN	B_2 vitamin
**5-Formyl-tetrahydrofolate**	fTHF	Folate species
**Glutamine**	Gln	Amino acid
**Glutamic acid**	Glu	Amino acid
**Glycine**	Gly	Amino acid
**3-Hydroxyanthranilic acid**	HAA	Tryptophan metabolite
**Homoarginine**	hArg	Amino acid
**Histidine**	Hist	Tryptophan metabolite
**3-Hydroxykynurenine**	HK	Tryptophan metabolite
**4-Alfa-hydroxy-5-methyl-THF**	hmTHF	Folate oxidation product
**Isoleucine**	Ile	Amino acid

For peer review only - http://bmjopen.bmj.com/site/about/guidelines.xhtml

**Kynurenic acid**	KA	Tryptophan metabolite
**Kynurenine**	Kyn	Tryptophan metabolite
**Leucine**	Leu	Amino acid
**Lysine**	Lys	Amino acid
**3-Methylhistidine (3-MH)**	m3His	Marker of muscle degradation and meat intake
**1-Methylhistidine (1-MH)**	m1His	Marker of muscle degradation and meat intake
**Methionine**	Met	Amino acid
**Methionine sulfoxide**	MetSo	Oxidation product of Met
**Methylmalonic acid**	MMA	Marker of B_{12} status
**N1-methylnicotinamide**	mNAM	B_3 vitamin
**5-Methyl-tetrahydrofolate**	mTHF	Folate species
**Nicotinic acid**	NA	B_3 vitamin
**Nicotinamide**	NAM	B_3 vitamin
**Neopterin**	Neopt	Inflammatory marker
**Trans-3'-hydroxycotinine**	OHCot	Nicotine metabolite
**Ornithine**	Orn	Amino acid
**4-Pyridoxic acid**	PA	Vitamin B_6 catabolite
**Para-aminobenzoylglutamate**	pABG	Folate catabolite
**Phenylalanine**	Phe	Amino acid
**Picolinic acid**	Pic	Tryptophan metabolite
**Pyridoxal**	PL	B_6 vitamin
**Pyridoxal 5'-phosphate**	PLP	B_6 vitamin
**Pyridoxine**	PN	Synthetic form of vitamin B_6
**Proline**	Pro	Amino acid
**Quinolinic acid**	QA	Tryptophan metabolite
**Riboflavin**	Ribo	Main circulating B_2 form
**Symmetric dimethylarginine**	SDMA	Marker of renal function
**Serine**	Ser	Amino acid
**Folate**	spFolate	Microbiologically active folate
**Total cysteine**	tCys	Amino acid
**Homocysteine**	tHcy	Marker of folate and B_{12} status
**Thiamine**	Thi	B_1 vitamin
**Threonine**	Thr	Amino acid
**Trimethylamineoxide**	TMAO	Choline metabolite
**Trimethyllysine**	TML	Amino acid
**Thiamine monophosphate**	TMP	B_1 vitamin
**Trigonelline**	Trig	Marker of coffee consumption
**Tryptophan**	Trp	Amino acid
**Valine**	Val	Amino acid
**Leucine**	Leu	Amino acid
**Xanthurenic acid**	XA	Tryptophan metabolite
**Kyn/Trp ratio**	KTR	Marker of immune activation
**HK/XA ratio**		Marker of B_6 status

For peer review only - http://bmjopen.bmj.com/site/about/guidelines.xhtml

What has the FOCUS Consortium found? Key findings and publications

The FOCUS Consortium provides a unique opportunity to conduct comprehensive research on folate and FOCM biomarkers status in the tertiary prevention of CRC using data collected both at diagnosis and during standardized follow-up time points. This well-characterized study design provides sufficient statistical power to discern prospective associations with relevant clinical outcomes, including CRC recurrence and survival within relevant subgroups.

Key findings and publications

We investigated associations of circulating concentrations of folate, folic acid, and folate catabolites pABG and apABG, measured around time of diagnosis, with recurrence and survival among 2024 patients diagnosed with stage I-III CRC within the international FOCUS Consortium. We did not observe any statistically significant associations for folate, pABG, and apABG concentrations. However, an increased risk of cancer recurrences was observed among patients with higher compared to lower circulating folic acid concentrations.[31] Further, Kiblawi et al. measured associations between one-carbon metabolites, inflammation- and angiogenesis-related biomarkers in a cross-sectional analysis of 238 patients from the ColoCare Heidelberg cohort. The study showed that specific folate species within the one-carbon metabolism pathway are associated with both inflammation and angiogenesis pathways among CRC patients. Our findings reinforce the notion that B vitamins involved in the one-carbon metabolism may be correlated with carcinogenic processes.[32] This and further research will support the evidence base needed for the development of dietary guidelines for CRC patients.

Further, we investigated circulating concentrations of nine biomarkers related to the B-vitamins folate, riboflavin, vitamin B_6, and cobalamin, measured at diagnosis and six months post-diagnosis, in association with health-related quality of life as assessed by the EORTC QLQ-C30 questionnaire six months post-treatment in three FOCUS cohorts (ColoCare Heidelberg, EnCoRe and COLON).[33] Higher pyridoxal 5'-phosphate (PLP) concentrations were cross-sectionally associated with better physical, role, and social functioning, and reduced fatigue six months post-diagnosis. Higher HKr (3-hydroxykynurenine:(kynurenic acid+xanthurenic acid+3-hydroxyanthranilic acid+anthranilic acid)), an inverse marker of vitamin B_6 status, was cross-sectionally associated with worse global quality of life, and lower physical and role functioning. Dose-response relations were observed for PLP with global

For peer review only - http://bmjopen.bmj.com/site/about/guidelines.xhtml

quality of life, physical, role, and social functioning. No associations were observed for changes in biomarker concentrations between diagnosis and six months with quality of life outcomes. We therefore concluded that higher vitamin B_6 status was associated with better quality of life at six months post treatment and that further study is needed to clarify the role of vitamin B_6 in relation to quality of life.

Previous relevant findings from individual cohorts within the consortium

To date, individual cohorts from the FOCUS Consortium have initiated the examination of dietary supplement use and dietary habits over time. Among CRC patients enrolled in the ColoCare Study the proportion of supplement users was found to be highest post-diagnosis (35%).[34] Moreover, within an international investigation including ColoCare participants from multiple sites, Ulrich et al. showed differences in plasma folate concentration between Heidelberg and the US sites, probably reflecting variation in folic acid fortification and supplement use.[11] Furthermore, ColoCare has published data on RECQ helicase expression[35], NTRK3[36], RET[37], tumor-infiltrating lymphocytes and T cell receptor sequences[38], 25-25-hydroxyvitamin D_3[11 39], DNA methylation[40-43], miRNAs[44 45], fecal microbiota[46-48], metabolomics and transcriptomics[49-51], plasma proteins[52], gene expression[53], branched-chain amino acids[54], genetic variants[55], body composition[51 56 57], physical activity[39], and dietary patterns[4] in CRC patients. Within the COLON study, results have been published on body weight trajectories[58], changes in lifestyle[59], 25-hydroxy vitamin D levels[60 61], and inflammation markers[62] over time, as well as vitamin D[62], calcium or magnesium intake[63], physical activity[64], inflammation[65 66], skeletal muscle mass[67]/ density[68] and other measures of body composition[69] in relation to cancer recurrence, survival, or physical functioning or fatigue. Moreover, in a subset of patients undergoing chemotherapy, dietary factors in relation to chronic CIPN[70 71] as well as chemosensory perception and food preferences[72] were studied. The Austrian CORSA Study has published results on genomic data[73-75], telomere length[76], DNA repair processes[77], tumor autoantibodies[78] as well as metabolomics.[31 79] To date, publications from the EnCoRe Study have reported on associations of physical activity and sedentary behaviour[80-83], adherence to lifestyle guidelines[84], and parameters of body composition[85 86] measured through CT scans with quality of life, functioning and fatigue in CRC survivors. Recently, longitudinal associations between supplement use and fatigue were investigated from diagnosis to 2 years post-CRC treatment. No overall association between supplement use and fatigue was found but results suggest that increased levels of fatigue may be a reason for the use of supplements among CRC survivors.[87] Higher concentrations of

For peer review only - http://bmjopen.bmj.com/site/about/guidelines.xhtml

25OHD3 were longitudinally associated with better global quality of life and less fatigue in colorectal cancer survivors within EnCoRe suggesting a potential beneficial role of vitamin D in colorectal cancer survivors.[33] In a mixed-method study using data of the EnCoRe study, colorectal cancer (treatment) related health and functioning problems negatively impacted the ability of nearly 1 in 5 long-term CRC survivors to participate in everyday life situations and their satisfaction with participation.[88] The validity of the food frequency questionnaire for measuring dietary intake among survivors of colorectal cancer within the EnCoRe study appeared to be moderate to good for most nutrients and food groups, relative to a 7-day dietary record.[89] Prediction models that we developed for estimating 1-year risk of low health-related quality of life in seven domains in colorectal cancer survivors performed well when externally validated among survivors within the EnCoRe and COLON studies.[90]

For peer review only - http://bmjopen.bmj.com/site/about/guidelines.xhtml

What are the main strengths and weaknesses of the FOCUS Consortium?

This is the largest consortium to date addressing the research question of folate and FOCM biomarkers in relation to survival, recurrence, treatment toxicity, and health-related quality of life outcomes in CRC patients. The cohorts included in the FOCUS Consortium are designed to enable future pooling of data.[20] For that reason, methodologies, time points and measurement instruments generally overlap, with each study presenting unique features such as additional blood collection at the 6-week follow-up time point within the EnCoRe Study and blood draws during chemotherapy within the COLON Study. The pooled sample size provides sufficient power to investigate subgroup analyses across CRC patients, e.g., within groups of patients who underwent 5-floururacil based chemotherapy or stratified by disease stage. Furthermore, the assessment of biomarkers related to FOCM are conducted at a single, state-of-the art laboratory and the biological materials are processed and stored according to standardized operation protocols across all study sites, enabling precise and accurate measurements of FOCM biomarkers. The study population is predominantly based on European cohorts (90.4%) in countries that have not implemented mandatory folic acid fortification. This enables us to study a population where dietary intake and dietary supplement use determine differences in folate status, yielding information of direct relevance to cancer patients. However, the generalizability of results to populations that have introduced folic acid fortification, including United States, might therefore also be limited.[91]

Another limitation of the consortium includes differences in collection strategies of the baseline study time point across included studies: CORSA, COLON and EnCoRe are recruiting CRC patients at diagnosis, preferably prior to any cancer treatment while ColoCare is recruiting patients prior to surgery (i.e. neoadjuvant treatment might have occurred prior to baseline blood and data collection). Nevertheless, the ColoCare protocol requires that no blood draw occurs within 2 weeks of neoadjuvant or adjuvant chemotherapy, limiting the influence of ongoing treatments on blood biomarkers. In some cohorts, the timing with respect to adjuvant chemotherapy is less clearly defined and this will be carefully considered in sensitivity analyses. Furthermore, survivor bias – a type of selection bias – may be introduced as some patients died or did not complete follow-up questionnaires or did not provide blood samples. It is possible that patients who experience more severe toxicities, worse clinical outcomes or worse health-related quality of life are underrepresented among those patients who completed follow-up measurements.[4]

For peer review only - http://bmjopen.bmj.com/site/about/guidelines.xhtml

Can I get hold of the data? Where can I find out more?

A substantial amount of time has been spent in creating the harmonized dataset of baseline variables from cohorts within the FOCUS Consortium, with follow-up data collection and FOCM biomarker analyses. Any person interested in learning about the FOCUS Consortium or in getting access to FOCUS data and in-depth analyses can contact the coordinating study PIs Prof. Andrea Gsur (andrea.gsur@meduniwien.ac.at) and Prof. Cornelia Ulrich (neli.ulrich@hci.utah.edu). Requests for data will be discussed and decided by all PIs and will require a Data Transfer Agreement.

For peer review only

For peer review only - http://bmjopen.bmj.com/site/about/guidelines.xhtml

FOCUS Consortium profile in a nutshell

- The FOCUS Consortium is an international prospective cohort with newly-diagnosed non-metastatic (stage I-III) colorectal cancer (CRC) patients, designed to study the associations between folate and folate-mediated one-carbon metabolism (FOCM) biomarkers and CRC prognosis, to provide clinically relevant advice to cancer patients and define future tertiary prevention strategies.
- 2,401 women and men age 18 years and older newly-diagnosed with a primary invasive CRC, stage I-III, from the ColoCare Study at the University of Heidelberg in Heidelberg, Germany; the ColoCare Study at the Huntsman Cancer Institute in Salt Lake City, Utah, USA; the ColoCare Study at the Fred Hutchinson Cancer Research Center in Seattle, Washington, USA; CORSA Study at the Medical University of Vienna in Austria; the COLON Study at Wageningen University in the Netherlands; and the EnCoRe Study at Maastricht University Medical Center in the Netherlands were eligible and recruited between 2003 and 2016.
- Blood samples at baseline were collected from n=2,132 (88.8%) study participants. N=1,537 (64.0%) participants donated blood at the 6 month and n=614 (25.6%) at the 12 month follow-up time points.
- The harmonized dataset comprises sociodemographic data and data on lifestyle behaviors (e.g., dietary supplement use, dietary nutrient intake, and physical activity), clinical data (e.g., cancer characteristics, treatment regimens), health-related quality of life, and biomarkers of one-carbon metabolism. Follow-up data additionally includes CRC recurrence, survival, treatment-induced toxicity, and health-related quality of life.
- We will welcome new collaborations, for which requests can be sent to the corresponding authors, PIs Prof. Andrea Gsur (andrea.gsur@meduniwien.ac.at) and Prof. Cornelia Ulrich (neli.ulrich@hci.utah.edu).

Acknowledgements

ColoCare Heidelberg: We thank all ColoCare study participants, the liquid biobank of the National Center for Tumor Diseases for storage of the blood samples according to the SOPs of the Biomaterialbank Heidelberg (BMBH), Michaela Magenreuter, Maria Thomalla-Starzl, Mareen, Dupovac, Marzena Knyssok-Sypniewski, Lin Zielske, Anett Brendel, Renate Skatula, and Marita Wenzel for laboratory work, Rifraz Farook, Dr. Werner Diehl and Inna Müller for data management and clinical follow-up data collection, Dr. Clare Abbenhardt-

For peer review only - http://bmjopen.bmj.com/site/about/guidelines.xhtml

Martin, Susanne Jakob, Judith Kammer for patient recruitment and follow-up, and Dr. Petra Schrotz-Kind and Dr. Jürgen Boehm for study coordination.

ColoCare Huntsman Cancer Institute: We thank all ColoCare study participants, the biorepository at Huntsman Cancer Institute for storage of the blood samples according to standardized SOPs, and Christy Warby for laboratory work. We thank Tyler Farr, Sarah Fischbuch and Debra Ma for data management and clinical follow-up data collection. We are very grateful for patient recruitment and follow-up by Elisa Santori, Hanna Omar-Payne and Marissa Grande, and Dr. Jürgen Boehm for study coordination.

ColoCare Fred Hutchinson Cancer Research Center: We thank all ColoCare study participants, the biorepository at Fred Hutchinson Cancer Research Center for storage of the blood samples according to standardized SOPs and the laboratory team for sample processing. We thank the ColoCare team for patient recruitment and follow-up and data management.

EnCoRe: We would like to thank all participants of the EnCoRe study and the health professionals in the three hospitals involved in the recruitment of participants of the study: Maastricht University Medical Center+, VieCuri Medical Center, and Zuyderland Medical Center. We would also like to thank the MEMIC center for data and information management for facilitating the logistic processes and data management of our study. Finally, we would like to thank the research dieticians and research assistant who are responsible for patient inclusion and follow-up, performing home visits, as well as data collection and processing.

CORSA: We kindly thank all individuals who agreed to participate in the CORSA study. Furthermore, we thank Thomas Bachleitner-Hofmann, Michael M. Bergmann, Judith Karner-Hanusch and Anton Stift (Department of Surgery, Medical University of Vienna), Karl Mach, Azita Deutinger-Permoon (KRAGES, Austria), and their co-workers for supporting CORSA recruitment. In addition, we would like to acknowledge Elisabeth Feik, Philipp Hofer and Cornelia Zöchmeister, for patient recruitment, sample processing, and follow-up.

COLON: The authors would like to thank the COLON participants, COLON investigators at Wageningen University & Research and the involved co-workers in the participating hospitals.

For peer review only

Contributors

BG, MW, EK, PMU, CMU, AG, ABU, NH: conceived of the project, developed the overall research plan, and provided study oversight; BG, EHvR, ANH, SB, AJMRG, AU, JO, JLK, VD, RK, TG, TL, GK, TK, DEK, FJBvD, MJLB, AB, CIL, WMG, KV: conducted hands-on experiments and data collection; BG, EHvR, ANH, SB, AJMRG, AU, JO, JLK, VD, RK, TG, TL, GK, TK, DEK, FJBvD, CIL, WMG, KV, CMU: provided essential reagents or essential materials, databases, and so forth, necessary for the research; BG, EHvR, MJLB, AB, ANH, VD, SB: analyzed the data or performed the statistical analysis and made a major contribution to writing the paper; BG, MS, EK, PMU, ABU, MW, AG, CMU: had primary responsibility for the final content; all authors: reviewed the manuscript critically, provided feedback, and read and approved the final manuscript.

Funding

The FOCUS Consortium is an ERA-NET (European Research Area Network) on Translational Cancer Research (TRANSCAN) project funded by the local research funding agencies (German Ministry of Education and Research, Germany, 01KT1503; Dutch Cancer Society with grant number UW2014-6877, the Netherlands; FWF Austrian Science Fund, Austria (API02104FW); Research Council Norway/Norwegian Cancer Society (RNC), Norway). The COLON study is further sponsored by Wereld Kanker Onderzoek Fonds, including funds from grant 2014/1179 as part of the World Cancer Research Fund International Regular Grant Programme; Alpe d'Huzes/Dutch Cancer Society (UM 2012-5653, UW 2013-5927, UW 2015-7946); and ERA-NET on Translational Cancer Research (TRANSCAN/Dutch Cancer Society: UW2013-6397 and the Netherlands Organization for Health Research and Development (ZonMw), the Netherlands). The EnCoRe study was supported by grants from the Stichting Alpe d'HuZes within the research program 'Leven met kanker' of the Dutch Cancer Society (Grant No. UM-2010-4867 and UM-2012-5653), and by grants from Kankeronderzoekfonds Limburg as part of Health Foundation Limburg (Grant No. 00005739), and by a grant from Wereld Kanker Onderzoek Fonds (WKOF), as part of the World Cancer Research Fund International grant programme (grant number 2016/1620).

Biljana Gigic was funded by the ERA-NET on Translational Cancer Research (TRANSCAN), the German Ministry of Education and Research project 01KT1503, the National Institutes of Health/National Cancer Institute (NHI/NCI) projects R01 CA189184 and U01 CA206110, the

For peer review only

Stiftung LebensBlicke, and the Matthias-Lackas Foundations. Eline H. van Roekel was funded by the Wereld Kanker Onderzoek Fonds (WKOF), part of the World Cancer Research Fund International grant programme (grant number 2016/1620). Martijn Bours was funded by Alpe d'HuZes within the research program 'Leven met kanker' of the Dutch Cancer Society (Grant No. UM-2012-5653). Dieuwertje E. Kok is supported by a Veni grant of the Netherlands Organisation for Scientific Research (grant no. 016.Veni.188.082). Andreana N. Holowatyj was supported by the National Institutes of Health under Ruth L. Kirschstein National Research Service Award T32 HG008962 from the National Human Genome Research Institute. Nina Habermann was funded by the TRANSCAN project 01KT1512, Cornelia M. Ulrich and Jennifer Ose were funded by the Huntsman Cancer Foundation, and NIH/NCI projects R01 CA189184, R01 CA207371, U01 CA206110, U01 CA206110 and P30 CA042014. NIH grants (P30 CA15704, U01 CA152756, R01 CA194663, R01 CA220004, P01 CA077852), Rodger C. Haggitt Endowed Chair, R.A.C.E. Charities, Cottrell Family Fund, Listwin Family Foundation, Seattle Translational Tumor Research program, Fred Hutchinson Cancer Research Center (William M. Grady). Stefanie Brezina was funded by the ERA-NET on Translational Cancer Research (TRANSCAN) project FOCUS I2104-B26 and the ERA-NET on Translational Cancer Research (TRANSCAN) project MetaboCCC I 1578 – B19. J.L. Koole was supported by Kankeronderzoekfonds Limburg as part of Health Foundation Limburg (Grant No. 00005739).

Disclaimer

The funder had no role in the design of the FOCUS consortium, or conduct of the study, collection, management, analysis, and interpretation of the data, preparation, review, or approval of the manuscript, or decision to submit the manuscript for publication.

Competing interests

None declared.

Patient consent for publication

Not applicable.

Patient and public involvement

Patients and/or the public were not involved in the design, or conduct, or reporting, or dissemination plans of this research.

For peer review only

For peer review only - http://bmjopen.bmj.com/site/about/guidelines.xhtml

Ethics approval

The Heidelberg ColoCare Study was approved by the Ethics Committee of the Medical Faculty of Heidelberg (approval no. S-310/2001 and S-134/2016). The ColoCare Study at the Huntsman Cancer Institute and the Fred Hutchinson Cancer Research Center were approved by the respective IRBs (#77147 and #6407). For the CORSA study all subjects gave written informed consent and the study was approved by the institutional review boards: 'Ethikkommission Burgenland' (33/2010), by the ethical review committee of the Medical University of Vienna (1160/2016) and by the "Ethikkommission der Stadt Wien" (EK 06-150VK). The EnCoRe Study was approved by the Medical Ethics Committee of the Maastricht University Hospital and Maastricht University (METC 11-3-075).

For peer review only

For peer review only - http://bmjopen.bmj.com/site/about/guidelines.xhtml

References

1. Ferlay J, Colombet M, Soerjomataram I, et al. Estimating the global cancer incidence and mortality in 2018: GLOBOCAN sources and methods. *International journal of cancer Journal international du cancer* 2019;144(8):1941-53. doi: 10.1002/ijc.31937 [published Online First: 2018/10/24]
2. De Angelis R, Sant M, Coleman MP, et al. Cancer survival in Europe 1999-2007 by country and age: results of EUROCARE--5-a population-based study. *The Lancet Oncology* 2014;15(1):23-34. doi: 10.1016/S1470-2045(13)70546-1
3. Ferlay J, Colombet M, Soerjomataram I, et al. Cancer incidence and mortality patterns in Europe: Estimates for 40 countries and 25 major cancers in 2018. *European journal of cancer* 2018;103:356-87. doi: 10.1016/j.ejca.2018.07.005 [published Online First: 2018/08/14]
4. Gigic B, Boeing H, Toth R, et al. Associations Between Dietary Patterns and Longitudinal Quality of Life Changes in Colorectal Cancer Patients: The ColoCare Study. *Nutrition and cancer* 2017:1-10. doi: 10.1080/01635581.2018.1397707 [published Online First: 2017/12/16]
5. Velicer CM, Ulrich CM. Vitamin and mineral supplement use among US adults after cancer diagnosis: a systematic review. *Journal of clinical oncology : official journal of the American Society of Clinical Oncology* 2008;26(4):665-73. doi: 10.1200/JCO.2007.13.5905
6. Ulrich CM, Potter JD. Folate supplementation: too much of a good thing? *Cancer Epidemiol Biomarkers Prev* 2006;15(2):189-93. doi: 10.1158/1055-9965.EPI-152CO [published Online First: 2006/02/24]
7. Jatoi A, Williams B, Nichols F, et al. Is voluntary vitamin and mineral supplementation associated with better outcome in non-small cell lung cancer patients? Results from the Mayo Clinic lung cancer cohort. *Lung Cancer* 2005;49(1):77-84. doi: 10.1016/j.lungcan.2005.01.004 [published Online First: 2005/06/14]
8. Ferrucci LM, McCorkle R, Smith T, et al. Factors related to the use of dietary supplements by cancer survivors. *J Altern Complement Med* 2009;15(6):673-80. doi: 10.1089/acm.2008.0387
9. Miller JW, Ulrich CM. Folic acid and cancer--where are we today? *Lancet* 2013;381(9871):974-6. doi: 10.1016/S0140-6736(13)60110-5 [published Online First: 2013/01/29]
10. Kim YI. Folate and colorectal cancer: an evidence-based critical review. *Mol Nutr Food Res* 2007;51(3):267-92. doi: 10.1002/mnfr.200600191 [published Online First: 2007/02/14]
11. Ulrich CM, Toriola AT, Siegel EM, et al. Plasma 25-hydroxyvitamin D3, folate and vitamin B12 biomarkers among international colorectal cancer patients: a pilot study. *J Nutr Sci* 2013;2:e9. doi: 10.1017/jns.2012.28
12. Cole BF, Baron JA, Sandler RS, et al. Folic acid for the prevention of colorectal adenomas: a randomized clinical trial. *Jama* 2007;297(21):2351-9. doi: 10.1001/jama.297.21.2351 [published Online First: 2007/06/07]
13. Lawrance AK, Deng L, Rozen R. Methylenetetrahydrofolate reductase deficiency and low dietary folate reduce tumorigenesis in Apc min/+ mice. *Gut* 2009;58(6):805-11. doi: 10.1136/gut.2007.143107 [published Online First: 2009/01/29]
14. Lindzon GM, Medline A, Sohn KJ, et al. Effect of folic acid supplementation on the progression of colorectal aberrant crypt foci. *Carcinogenesis* 2009;30(9):1536-43. doi: 10.1093/carcin/bgp152 [published Online First: 2009/06/23]
15. Mason JB. Folate, cancer risk, and the Greek god, Proteus: a tale of two chameleons. *Nutrition reviews* 2009;67(4):206-12. doi: 10.1111/j.1753-4887.2009.00190.x [published Online First: 2009/04/02]
16. Ulrich CM, Miller JW. Folate and health: present and future. *Mol Nutr Food Res* 2013;57(4):561. doi: 10.1002/mnfr.201370034 [published Online First: 2013/04/05]
17. Choi JH, Yates Z, Martin C, et al. Gene-Nutrient Interaction between Folate and Dihydrofolate Reductase in Risk for Adenomatous Polyp Occurrence: A Preliminary Report. *J Nutr Sci Vitaminol (Tokyo)* 2015;61(6):455-9. doi: 10.3177/jnsv.61.455 [published Online First: 2016/02/16]
18. Sawaengsri H, Wang J, Reginaldo C, et al. High folic acid intake reduces natural killer cell cytotoxicity in aged mice. *J Nutr Biochem* 2016;30:102-7. doi: 10.1016/j.jnutbio.2015.12.006 [published Online First: 2016/03/26]

For peer review only - http://bmjopen.bmj.com/site/about/guidelines.xhtml

19. Ulrich CM, Gigic B, Bohm J, et al. The ColoCare Study: A Paradigm of Transdisciplinary Science in Colorectal Cancer Outcomes. *Cancer Epidemiol Biomarkers Prev* 2019;28(3):591-601. doi: 10.1158/1055-9965.EPI-18-0773 [published Online First: 2018/12/14]

20. Winkels RM, Heine-Broring RC, van Zutphen M, et al. The COLON study: Colorectal cancer: Longitudinal, Observational study on Nutritional and lifestyle factors that may influence colorectal tumour recurrence, survival and quality of life. *BMC cancer* 2014;14:374. doi: 10.1186/1471-2407-14-374 [published Online First: 2014/06/03]

21. van Roekel EH, Bours MJ, de Brouwer CP, et al. The applicability of the international classification of functioning, disability, and health to study lifestyle and quality of life of colorectal cancer survivors. *Cancer Epidemiol Biomarkers Prev* 2014;23(7):1394-405. doi: 10.1158/1055-9965.EPI-13-1144 [published Online First: 2014/05/08]

22. Fayers P, Bottomley A, Group EQoL, et al. Quality of life research within the EORTC-the EORTC QLQ-C30. European Organisation for Research and Treatment of Cancer. *European journal of cancer* 2002;38 Suppl 4:S125-33.

23. Postma TJ, Aaronson NK, Heimans JJ, et al. The development of an EORTC quality of life questionnaire to assess chemotherapy-induced peripheral neuropathy: the QLQ-CIPN20. *European journal of cancer* 2005;41(8):1135-9. doi: 10.1016/j.ejca.2005.02.012

24. Molloy AM, Scott JM. Microbiological assay for serum, plasma, and red cell folate using cryopreserved, microtiter plate method. *Methods Enzymol* 1997;281:43-53. [published Online First: 1997/01/01]

25. Kelleher BP, Broin SD. Microbiological assay for vitamin B12 performed in 96-well microtitre plates. *J Clin Pathol* 1991;44(7):592-5. [published Online First: 1991/07/01]

26. Hannisdal R, Ueland PM, Svardal A. Liquid chromatography-tandem mass spectrometry analysis of folate and folate catabolites in human serum. *Clin Chem* 2009;55(6):1147-54. doi: 10.1373/clinchem.2008.114389 [published Online First: 2009/04/11]

27. Midttun O, Hustad S, Ueland PM. Quantitative profiling of biomarkers related to B-vitamin status, tryptophan metabolism and inflammation in human plasma by liquid chromatography/tandem mass spectrometry. *Rapid Commun Mass Spectrom* 2009;23(9):1371-9. doi: 10.1002/rcm.4013 [published Online First: 2009/04/02]

28. Midttun O, Kvalheim G, Ueland PM. High-throughput, low-volume, multianalyte quantification of plasma metabolites related to one-carbon metabolism using HPLC-MS/MS. *Anal Bioanal Chem* 2013;405(6):2009-17. doi: 10.1007/s00216-012-6602-6 [published Online First: 2012/12/13]

29. Midttun O, McCann A, Aarseth O, et al. Combined Measurement of 6 Fat-Soluble Vitamins and 26 Water-Soluble Functional Vitamin Markers and Amino Acids in 50 muL of Serum or Plasma by High-Throughput Mass Spectrometry. *Anal Chem* 2016;88(21):10427-36. doi: 10.1021/acs.analchem.6b02325 [published Online First: 2016/11/02]

30. Meyer K, Ueland PM. Targeted quantification of C-reactive protein and cystatin c and its variants by immuno-MALDI-MS. *Anal Chem* 2014;86(12):5807-14. doi: 10.1021/ac500704y [published Online First: 2014/05/23]

31. Geijsen A, Ulvik A, Gigic B, et al. Circulating Folate and Folic Acid Concentrations: Associations With Colorectal Cancer Recurrence and Survival. *JNCI Cancer Spectr* 2020;4(5):pkaa051. doi: 10.1093/jncics/pkaa051 [published Online First: 2020/11/03]

32. Kiblawi R, Holowatyj AN, Gigic B, et al. One-carbon metabolites, B-vitamins and associations with systemic inflammation and angiogenesis biomarkers among colorectal cancer patients: results from the ColoCare Study. *The British journal of nutrition* 2020:1-32. doi: 10.1017/S0007114520000422 [published Online First: 2020/02/06]

33. Koole JL, Bours MJL, Geijsen A, et al. Circulating B-vitamin biomarkers and B-vitamin supplement use in relation to quality of life in patients with colorectal cancer: results from the FOCUS consortium. *The American journal of clinical nutrition* 2021 doi: 10.1093/ajcn/nqaa422 [published Online First: 2021/03/06]

34. Schrotz-King P, Dölp K, Paskow MJ, et al. Dietary Supplement Use among German Colorectal Cancer Patients: the ColoCare Study: AACR/MEG Post-GWAS Horizons in Molecular Epidemiology: Digging Deeper into the Environment., 2012.

35. Lao VV, Welcsh P, Luo Y, et al. Altered RECQ Helicase Expression in Sporadic Primary Colorectal Cancers. *Transl Oncol* 2013;6(4):458-69.

For peer review only

For peer review only - http://bmjopen.bmj.com/site/about/guidelines.xhtml

36. Luo Y, Kaz AM, Kanngurn S, et al. NTRK3 is a potential tumor suppressor gene commonly inactivated by epigenetic mechanisms in colorectal cancer. *PLoS Genet* 2013;9(7):e1003552. doi: 10.1371/journal.pgen.1003552

37. Luo Y, Tsuchiya KD, Il Park D, et al. RET is a potential tumor suppressor gene in colorectal cancer. *Oncogene* 2013;32(16):2037-47. doi: 10.1038/onc.2012.225

38. Sherwood AM, Emerson RO, Scherer D, et al. Tumor-infiltrating lymphocytes in colorectal tumors display a diversity of T cell receptor sequences that differ from the T cells in adjacent mucosal tissue. *Cancer Immunol Immunother* 2013;62(9):1453-61. doi: 10.1007/s00262-013-1446-2

39. Skender S, Bohm J, Schrotz-King P, et al. Plasma 25-Hydroxyvitamin D3 Levels in Colorectal Cancer Patients and Associations with Physical Activity. *Nutrition and cancer* 2017;69(2):229-37. doi: 10.1080/01635581.2017.1265131

40. Luo Y, Wong CJ, Kaz AM, et al. Differences in DNA methylation signatures reveal multiple pathways of progression from adenoma to colorectal cancer. *Gastroenterology* 2014;147(2):418-29 e8. doi: 10.1053/j.gastro.2014.04.039

41. Barrow TM, Klett H, Toth R, et al. Smoking is associated with hypermethylation of the APC 1A promoter in colorectal cancer: the ColoCare Study. *J Pathol* 2017;243(3):366-75. doi: 10.1002/path.4955 [published Online First: 2017/08/10]

42. Yu M, Carter KT, Makar KW, et al. MethyLight droplet digital PCR for detection and absolute quantification of infrequently methylated alleles. *Epigenetics* 2015;10(9):803-9. doi: 10.1080/15592294.2015.1068490

43. Klett H, Balavarca Y, Toth R, et al. Robust prediction of gene regulation in colorectal cancer tissues from DNA methylation profiles. *Epigenetics* 2018;13(4):386-97. doi: 10.1080/15592294.2018.1460034 [published Online First: 2018/04/27]

44. Ristau J, Staffa J, Schrotz-King P, et al. Suitability of circulating miRNAs as potential prognostic markers in colorectal cancer. *Cancer Epidemiol Biomarkers Prev* 2014;23(12):2632-7. doi: 10.1158/1055-9965.EPI-14-0556

45. Yuan Z, Baker K, Redman MW, et al. Dynamic plasma microRNAs are biomarkers for prognosis and early detection of recurrence in colorectal cancer. *British journal of cancer* 2017;117(8):1202-10. doi: 10.1038/bjc.2017.266 [published Online First: 2017/08/16]

46. Zeller G, Tap J, Voigt AY, et al. Potential of fecal microbiota for early-stage detection of colorectal cancer. *Mol Syst Biol* 2014;10:766. doi: 10.15252/msb.20145645

47. Thomas AM, Manghi P, Asnicar F, et al. Metagenomic analysis of colorectal cancer datasets identifies cross-cohort microbial diagnostic signatures and a link with choline degradation. *Nat Med* 2019;25(4):667-78. doi: 10.1038/s41591-019-0405-7 [published Online First: 2019/04/03]

48. Wirbel J, Pyl PT, Kartal E, et al. Meta-analysis of fecal metagenomes reveals global microbial signatures that are specific for colorectal cancer. *Nat Med* 2019;25(4):679-89. doi: 10.1038/s41591-019-0406-6 [published Online First: 2019/04/03]

49. Liesenfeld DB, Grapov D, Fahrmann JF, et al. Metabolomics and transcriptomics identify pathway differences between visceral and subcutaneous adipose tissue in colorectal cancer patients: the ColoCare study. *The American journal of clinical nutrition* 2015;102(2):433-43. doi: 10.3945/ajcn.114.103804

50. Liesenfeld DB, Habermann N, Toth R, et al. Changes in urinary metabolic profiles of colorectal cancer patients enrolled in a prospective cohort study (ColoCare). *Metabolomics* 2015;11(4):998-1012. doi: 10.1007/s11306-014-0758-3 [published Online First: 2015/08/01]

51. Ose J, Gigic B, Lin T, et al. Multiplatform Urinary Metabolomics Profiling to Discriminate Cachectic from Non-Cachectic Colorectal Cancer Patients: Pilot Results from the ColoCare Study. *Metabolites* 2019;9(9) doi: 10.3390/metabo9090178 [published Online First: 2019/09/11]

52. Bohm J, Pianka F, Stuttgen N, et al. Discovery of novel plasma proteins as biomarkers for the development of incisional hernias after midline incision in patients with colorectal cancer: The ColoCare study. *Surgery* 2017;161(3):808-17. doi: 10.1016/j.surg.2016.08.025

53. Gong J, Hutter CM, Newcomb PA, et al. Genome-Wide Interaction Analyses between Genetic Variants and Alcohol Consumption and Smoking for Risk of Colorectal Cancer. *PLoS Genet*

For peer review only - http://bmjopen.bmj.com/site/about/guidelines.xhtml

2016;12(10):e1006296. doi: 10.1371/journal.pgen.1006296 [published Online First: 2016/10/11]
54. Delphan M, Lin T, Liesenfeld DB, et al. Associations of branched-chain amino acids with parameters of energy balance and survival in colorectal cancer patients: Results from the ColoCare Study. *Metabolomics* 2018;2018(14) doi: 10.1007/s11306-017-1314-8 [published Online First: 2018/05/01]
55. Rosenthal EA, Shirts BH, Amendola LM, et al. Rare loss of function variants in candidate genes and risk of colorectal cancer. *Hum Genet* 2018 doi: 10.1007/s00439-018-1938-4 [published Online First: 2018/09/30]
56. Nattenmueller J, Hoegenauer H, Boehm J, et al. CT-based compartmental quantification of adipose tissue versus body metrics in colorectal cancer patients. *Eur Radiol* 2016;26(11):4131-40. doi: 10.1007/s00330-016-4231-8
57. Ozoya OO, Siegel EM, Srikumar T, et al. Quantitative Assessment of Visceral Obesity and Postoperative Colon Cancer Outcomes. *J Gastrointest Surg* 2017;21(3):534-42. doi: 10.1007/s11605-017-3362-9 [published Online First: 2017/01/20]
58. van Zutphen M, Geelen A, Boshuizen HC, et al. Pre-to-post diagnosis weight trajectories in colorectal cancer patients with non-metastatic disease. *Supportive care in cancer : official journal of the Multinational Association of Supportive Care in Cancer* 2019;27(4):1541-49. doi: 10.1007/s00520-018-4560-z [published Online First: 2018/11/30]
59. van Zutphen M, Boshuizen HC, Kok DE, et al. Colorectal cancer survivors only marginally change their overall lifestyle in the first 2 years following diagnosis. *J Cancer Surviv* 2019;13(6):956-67. doi: 10.1007/s11764-019-00812-7 [published Online First: 2019/10/28]
60. Wesselink E, Bours MJL, de Wilt JHW, et al. Chemotherapy and vitamin D supplement use are determinants of serum 25-hydroxyvitamin D levels during the first six months after colorectal cancer diagnosis. *J Steroid Biochem Mol Biol* 2020;199:105577. doi: 10.1016/j.jsbmb.2020.105577 [published Online First: 2020/01/10]
61. Wesselink E, Kok DE, de Wilt JHW, et al. Sufficient 25-Hydroxyvitamin D Levels 2 Years after Colorectal Cancer Diagnosis are Associated with a Lower Risk of All-cause Mortality. *Cancer Epidemiol Biomarkers Prev* 2021;30(4):765-73. doi: 10.1158/1055-9965.EPI-20-1388 [published Online First: 2021/02/04]
62. Wesselink E, Balvers M, Bours MJL, et al. The association between circulating levels of vitamin D and inflammatory markers in the first 2 years after colorectal cancer diagnosis. *Therap Adv Gastroenterol* 2020;13:1756284820923922. doi: 10.1177/1756284820923922 [published Online First: 2020/06/18]
63. Wesselink E, Kok DE, Bours MJL, et al. Vitamin D, magnesium, calcium, and their interaction in relation to colorectal cancer recurrence and all-cause mortality. *The American journal of clinical nutrition* 2020;111(5):1007-17. doi: 10.1093/ajcn/nqaa049 [published Online First: 2020/03/20]
64. van Zutphen M, Winkels RM, van Duijnhoven FJ, et al. An increase in physical activity after colorectal cancer surgery is associated with improved recovery of physical functioning: a prospective cohort study. *BMC cancer* 2017;17(1):74. doi: 10.1186/s12885-017-3066-2 [published Online First: 2017/01/27]
65. Wesselink E, van Baar H, van Zutphen M, et al. Inflammation Is a Mediating Factor in the Association between Lifestyle and Fatigue in Colorectal Cancer Patients. *Cancers (Basel)* 2020;12(12) doi: 10.3390/cancers12123701 [published Online First: 2020/12/16]
66. Wesselink E, Staritsky LE, van Zutphen M, et al. The association between the adapted dietary inflammatory index and colorectal cancer recurrence and all-cause mortality. *Clin Nutr* 2021;40(6):4436-43. doi: 10.1016/j.clnu.2021.01.004 [published Online First: 2021/01/23]
67. van Baar H, Winkels RM, Brouwer JGM, et al. Associations of Abdominal Skeletal Muscle Mass, Fat Mass, and Mortality among Men and Women with Stage I-III Colorectal Cancer. *Cancer Epidemiol Biomarkers Prev* 2020;29(5):956-65. doi: 10.1158/1055-9965.EPI-19-1134 [published Online First: 2020/03/07]
68. van Baar H, Beijer S, Bours MJL, et al. Low radiographic muscle density is associated with lower overall and disease-free survival in early-stage colorectal cancer patients. *J Cancer Res Clin Oncol* 2018;144(11):2139-47. doi: 10.1007/s00432-018-2736-z [published Online First: 2018/08/19]

For peer review only - http://bmjopen.bmj.com/site/about/guidelines.xhtml

69. van Baar H, Bours MJL, Beijer S, et al. Body composition and its association with fatigue in the first 2 years after colorectal cancer diagnosis. *J Cancer Surviv* 2021;15(4):597-606. doi: 10.1007/s11764-020-00953-0 [published Online First: 2020/10/18]

70. Winkels RM, van Brakel L, van Baar H, et al. Are Ergothioneine Levels in Blood Associated with Chronic Peripheral Neuropathy in Colorectal Cancer Patients Who Underwent Chemotherapy? *Nutrition and cancer* 2020;72(3):451-59. doi: 10.1080/01635581.2019.1637005 [published Online First: 2019/07/13]

71. Wesselink E, Winkels RM, van Baar H, et al. Dietary Intake of Magnesium or Calcium and Chemotherapy-Induced Peripheral Neuropathy in Colorectal Cancer Patients. *Nutrients* 2018;10(4) doi: 10.3390/nu10040398 [published Online First: 2018/03/24]

72. Postma EM, Kok DE, de Graaf C, et al. Chemosensory perception and food preferences in colorectal cancer patients undergoing adjuvant chemotherapy. *Clin Nutr ESPEN* 2020;40:242-51. doi: 10.1016/j.clnesp.2020.09.012 [published Online First: 2020/11/14]

73. Hofer P, Hagmann M, Brezina S, et al. Bayesian and frequentist analysis of an Austrian genome-wide association study of colorectal cancer and advanced adenomas. *Oncotarget* 2017;8(58):98623-34. doi: 10.18632/oncotarget.21697 [published Online First: 2017/12/13]

74. Schmit SL, Edlund CK, Schumacher FR, et al. Novel Common Genetic Susceptibility Loci for Colorectal Cancer. *Journal of the National Cancer Institute* 2019;111(2):146-57. doi: 10.1093/jnci/djy099 [published Online First: 2018/06/20]

75. Schafmayer C, Harrison JW, Buch S, et al. Genome-wide association analysis of diverticular disease points towards neuromuscular, connective tissue and epithelial pathomechanisms. *Gut* 2019 doi: 10.1136/gutjnl-2018-317619 [published Online First: 2019/01/21]

76. Zochmeister C, Brezina S, Hofer P, et al. Leukocyte telomere length throughout the continuum of colorectal carcinogenesis. *Oncotarget* 2018;9(17):13582-92. doi: 10.18632/oncotarget.24431 [published Online First: 2018/03/24]

77. Jiraskova K, Hughes DJ, Brezina S, et al. Functional Polymorphisms in DNA Repair Genes Are Associated with Sporadic Colorectal Cancer Susceptibility and Clinical Outcome. *Int J Mol Sci* 2018;20(1) doi: 10.3390/ijms20010097 [published Online First: 2018/12/29]

78. Luna Coronell JA, Sergelen K, Hofer P, et al. The Immunome of Colon Cancer: Functional In Silico Analysis of Antigenic Proteins Deduced from IgG Microarray Profiling. *Genomics Proteomics Bioinformatics* 2018;16(1):73-84. doi: 10.1016/j.gpb.2017.10.002 [published Online First: 2018/03/06]

79. Geijsen A, van Roekel EH, van Duijnhoven FJB, et al. Plasma metabolites associated with colorectal cancer stage: Findings from an international consortium. *International journal of cancer Journal international du cancer* 2019 doi: 10.1002/ijc.32666 [published Online First: 2019/09/10]

80. Van Roekel EH, Bours MJ, Breedveld-Peters JJ, et al. Light Physical Activity Is Associated with Quality of Life after Colorectal Cancer. *Med Sci Sports Exerc* 2015;47(12):2493-503. doi: 10.1249/MSS.0000000000000698 [published Online First: 2015/05/15]

81. van Roekel EH, Winkler EA, Bours MJ, et al. Associations of sedentary time and patterns of sedentary time accumulation with health-related quality of life in colorectal cancer survivors. *Prev Med Rep* 2016;4:262-9. doi: 10.1016/j.pmedr.2016.06.022 [published Online First: 2016/07/16]

82. van Roekel EH, Bours MJ, Breedveld-Peters JJ, et al. Modeling how substitution of sedentary behavior with standing or physical activity is associated with health-related quality of life in colorectal cancer survivors. *Cancer causes & control : CCC* 2016;27(4):513-25. doi: 10.1007/s10552-016-0725-6 [published Online First: 2016/02/20]

83. van Roekel EH, Duchateau J, Bours MJL, et al. Longitudinal associations of light-intensity physical activity with quality of life, functioning and fatigue after colorectal cancer. *Quality of life research : an international journal of quality of life aspects of treatment, care and rehabilitation* 2020;29(11):2987-98. doi: 10.1007/s11136-020-02566-7 [published Online First: 2020/07/04]

84. Breedveld-Peters JJL, Koole JL, Muller-Schulte E, et al. Colorectal cancers survivors' adherence to lifestyle recommendations and cross-sectional associations with health-related quality of life. *The British journal of nutrition* 2018;120(2):188-97. doi: 10.1017/S0007114518000661 [published Online First: 2018/04/17]

For peer review only - http://bmjopen.bmj.com/site/about/guidelines.xhtml

85. van Roekel EH, Bours MJL, Te Molder MEM, et al. Associations of adipose and muscle tissue parameters at colorectal cancer diagnosis with long-term health-related quality of life. *Quality of life research : an international journal of quality of life aspects of treatment, care and rehabilitation* 2017;26(7):1745-59. doi: 10.1007/s11136-017-1539-z [published Online First: 2017/03/21]
86. van Heinsbergen M, Konsten JL, Bours MJL, et al. Preoperative handgrip strength is not associated with complications and health-related quality of life after surgery for colorectal cancer. *Sci Rep* 2020;10(1):13005. doi: 10.1038/s41598-020-69806-1 [published Online First: 2020/08/05]
87. Koole JL, Bours MJL, Breedveld-Peters JJL, et al. Is dietary supplement use longitudinally associated with fatigue in stage I-III colorectal cancer survivors? *Clin Nutr* 2019 doi: 10.1016/j.clnu.2018.12.037 [published Online First: 2019/02/17]
88. Breedveld-Peters JJL, Bours MJL, Cords CI, et al. The impact of participation restrictions on everyday life in long-term colorectal cancer survivors in the EnCoRe study: A mixed-method study. *Eur J Oncol Nurs* 2020;45:101724. doi: 10.1016/j.ejon.2020.101724 [published Online First: 2020/02/12]
89. Koole JL, Bours MJL, Breedveld-Peters JJL, et al. Evaluating the Validity of a Food Frequency Questionnaire in Comparison with a 7-Day Dietary Record for Measuring Dietary Intake in a Population of Survivors of Colorectal Cancer. *J Acad Nutr Diet* 2020;120(2):245-57. doi: 10.1016/j.jand.2019.09.008 [published Online First: 2019/12/07]
90. Revesz D, van Kuijk SMJ, Mols F, et al. Development and internal validation of prediction models for colorectal cancer survivors to estimate the 1-year risk of low health-related quality of life in multiple domains. *BMC Med Inform Decis Mak* 2020;20(1):54. doi: 10.1186/s12911-020-1064-9 [published Online First: 2020/03/14]
91. Ose J, Botma A, Balavarca Y, et al. Pathway analysis of genetic variants in folate-mediated one-carbon metabolism-related genes and survival in a prospectively followed cohort of colorectal cancer patients. *Cancer Med* 2018 doi: 10.1002/cam4.1407 [published Online First: 2018/05/31]

For peer review only - http://bmjopen.bmj.com/site/about/guidelines.xhtml

Figure legends

Figure 1 FOCUS Consortium design

For peer review only

For peer review only - http://bmjopen.bmj.com/site/about/guidelines.xhtml

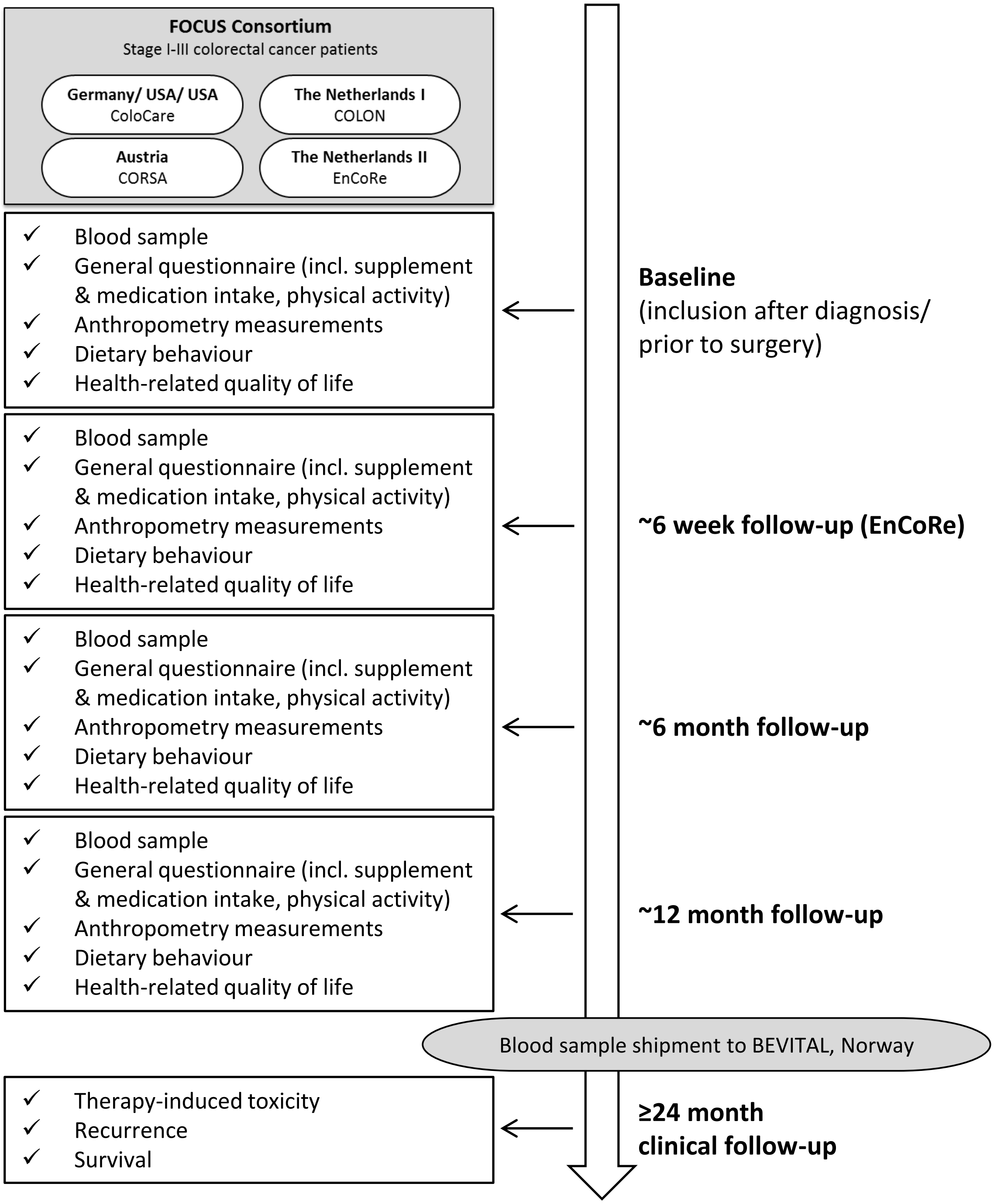
FOCUS Consortium
Stage I-III colorectal cancer patients
Germany/ USA/ USA
ColoCare
The Netherlands I
COLON
Austria
CORSA
The Netherlands II
EnCoRe
✓ Blood sample
✓ General questionnaire (incl. supplement & medication intake, physical activity)
✓ Anthropometry measurements
✓ Dietary behaviour
✓ Health-related quality of life
Baseline
(inclusion after diagnosis/ prior to surgery)
✓ Blood sample
✓ General questionnaire (incl. supplement & medication intake, physical activity)
✓ Anthropometry measurements
✓ Dietary behaviour
✓ Health-related quality of life
~6 week follow-up (EnCoRe)
✓ Blood sample
✓ General questionnaire (incl. supplement & medication intake, physical activity)
✓ Anthropometry measurements
✓ Dietary behaviour
✓ Health-related quality of life
~6 month follow-up
✓ Blood sample
✓ General questionnaire (incl. supplement & medication intake, physical activity)
✓ Anthropometry measurements
✓ Dietary behaviour
✓ Health-related quality of life
~12 month follow-up
Blood sample shipment to BEVITAL, Norway
✓ Therapy-induced toxicity
✓ Recurrence
✓ Survival
≥24 month
clinical follow-up

BMJ Open

Cohort profile: Biomarkers related to folate-dependent one-carbon metabolism in colorectal cancer recurrence and survival: The FOCUS Consortium

Journal:	*BMJ Open*
Manuscript ID	bmjopen-2022-062930.R1
Article Type:	Cohort profile
Date Submitted by the Author:	07-Sep-2022

Complete List of Authors:	Gigic, Biljana; University Hospital Heidelberg, Department of General, Visceral and Transplantation Surgery<br>van Roekel, Eline ; Maastricht University, Department of Epidemiology<br>Holowatyj, Andreana; Vanderbilt University Medical Center, Medicine; Vanderbilt-Ingram Cancer Center,<br>Brezina, Stefanie; Medical University of Vienna, Institute of Cancer Research, Department of Medicine I<br>Geijsen, Anne; Wageningen University & Research, Division of Human Nutrition and Health<br>Ulvik, Arve; BEVITAL<br>Ose, Jennifer; Huntsman Cancer Institute; University of Utah, Department of Population Health Sciences<br>Koole, Janna L.; Maastricht University, Department of Epidemiology<br>Damerell, Victoria; University Hospital Heidelberg, Department of General, Visceral and Transplantation Surgery<br>Kiblawi, Rama; Huntsman Cancer Institute; University of Utah, Department of Population Health Sciences<br>Gumpenberger, Tanja; Medical University of Vienna, Institute of Cancer Research, Department of Medicine I<br>Lin, Tengda; Huntsman Cancer Institute; University of Utah, Department of Population Health Sciences<br>Kvalheim, Gry; BEVITAL<br>Koelsch, Torsten; Heidelberg University Hospital, Department of General, Visceral and Transplantation Surgery<br>Kok, Dieuwertje; Wageningen University & Research, Division of Human Nutrition and Health<br>van Duijnhoven, Franzel J; Wageningen University & Research, Division of Human Nutrition and Health<br>Bours, Martijn J.; Maastricht University, Department of Epidemiology<br>Baierl, Andreas; University of Vienna, Department of Statistics and Operations Research<br>Li, Christopher I.; Fred Hutchinson Cancer Research Center<br>Grady, William; Fred Hutchinson Cancer Research Center; University of Washington School of Medicine, Department of Medicine<br>Vickers, Kathy; Fred Hutchinson Cancer Research Center<br>Habermann, Nina; European Molecular Biology Laboratory (EMBL), Genome Biology<br>Schneider, Martin; Heidelberg University Hospital, Department of

	General, Visceral and Transplantation Surgery<br>Kampman, Ellen; Wageningen University & Research, Division of Human Nutrition and Health<br>Ueland, Per Magne; BEVITAL<br>Ulrich, Alexis; Städtische Kliniken Neuss, Lukaskrankenhaus GmbH, Surgical Department I; University Hospital Heidelberg, Department of General, Visceral and Transplantation Surgery<br>Weijenberg, Matty ; Maastricht University, Department of Epidemiology<br>Gsur, Andrea; Medical University of Vienna, Institute of Cancer Research, Department of Medicine I<br>Ulrich, Cornelia; Huntsman Cancer Institute, Population Sciences; University of Utah, Department of Population Health Sciences
**Primary Subject Heading**:	Epidemiology
Secondary Subject Heading:	Nutrition and metabolism, Oncology, Public health
Keywords:	Epidemiology < ONCOLOGY, Nutritional support < ONCOLOGY, Gastrointestinal tumours < ONCOLOGY

SCHOLARONE™
Manuscripts

I, the Submitting Author has the right to grant and does grant on behalf of all authors of the Work (as defined in the below author licence), an exclusive licence and/or a non-exclusive licence for contributions from authors who are: i) UK Crown employees; ii) where BMJ has agreed a CC-BY licence shall apply, and/or iii) in accordance with the terms applicable for US Federal Government officers or employees acting as part of their official duties; on a worldwide, perpetual, irrevocable, royalty-free basis to BMJ Publishing Group Ltd ("BMJ") its licensees and where the relevant Journal is co-owned by BMJ to the co-owners of the Journal, to publish the Work in this journal and any other BMJ products and to exploit all rights, as set out in our licence.

The Submitting Author accepts and understands that any supply made under these terms is made by BMJ to the Submitting Author unless you are acting as an employee on behalf of your employer or a postgraduate student of an affiliated institution which is paying any applicable article publishing charge ("APC") for Open Access articles. Where the Submitting Author wishes to make the Work available on an Open Access basis (and intends to pay the relevant APC), the terms of reuse of such Open Access shall be governed by a Creative Commons licence – details of these licences and which Creative Commons licence will apply to this Work are set out in our licence referred to above.

Other than as permitted in any relevant BMJ Author's Self Archiving Policies, I confirm this Work has not been accepted for publication elsewhere, is not being considered for publication elsewhere and does not duplicate material already published. I confirm all authors consent to publication of this Work and authorise the granting of this licence.

For peer review only

Cohort profile: Biomarkers related to folate-dependent one-carbon metabolism in colorectal cancer recurrence and survival: The FOCUS Consortium

Authors:
Biljana Gigic[1], Eline H. van Roekel[2], Andreana N. Holowatyj[3,4,5,6], Stefanie Brezina[7], Anne J.M.R. Geijsen[8], Arve Ulvik[9], Jennifer Ose[3,4], Janna L. Koole[2], Victoria Damerell[1], Rama Kiblawi[3,4], Tanja Gumpenberger[7], Tengda Lin[3,4], Gry Kvalheim[9], Torsten Koelsch[1], Dieuwertje E. Kok[8], Fränzel J.B. van Duijnhoven[8], Martijn J. L. Bours[2], Andreas Baierl[10], Christopher I. Li[11], William M. Grady[11,12,13], Kathy Vickers[11], Nina Habermann[14], Martin Schneider[1], Ellen Kampman[8,16], Per-Magne Ueland[9,16], Alexis B. Ulrich[1,15,16], Matty Weijenberg[2,16], Andrea Gsur[7,16,17] and Cornelia M. Ulrich[3,4,16,17]

Affiliations:
[1] Department of General, Visceral and Transplantation Surgery, Heidelberg University Hospital, Heidelberg, Germany
[2] Department of Epidemiology, Maastricht University, Maastricht, the Netherlands
[3] Huntsman Cancer Institute, Salt Lake City, UT
[4] Department of Population Health Sciences, University of Utah, Salt Lake City, UT
[5] Department of Medicine, Vanderbilt University Medical Center, Nashville, TN
[6] Vanderbilt-Ingram Cancer Center, Nashville, TN
[7] Institute of Cancer Research, Department of Medicine I, Medical University of Vienna, Austria
[8] Division of Human Nutrition and Health, Wageningen University & Research, Wageningen, The Netherlands
[9] BEVITAL, Bergen, Norway
[10] Department of Statistics and Operations Research, University of Vienna, Austria
[11] Public Health Sciences Division, Fred Hutchinson Cancer Research Center, Seattle, Washington
[12] Department of Medicine, University of Washington School of Medicine, Seattle, Washington
[13] Clinical Research Division, Fred Hutchinson Cancer Research Center, Seattle, Washington
[14] Genome Biology, European Molecular Biology Laboratory (EMBL), Heidelberg, Germany
[15] Department of Surgery I, Lukas Hospital Neuss, Neuss, Germany
[16] These authors contributed equally
[17] Corresponding authors

Keywords: cohort, consortium, B-vitamins, folate, folic acid, one-carbon metabolism, dietary supplements, biomarkers, treatment toxicity, colorectal cancer, recurrence, survival

Corresponding author:
Cornelia M. Ulrich, MS, PhD
Chief Scientific Officer and Executive Director of the Cancer Center
Huntsman Cancer Institute
2000 Circle of Hope Drive
Salt Lake City, UT 84112
Phone: +1 (801) 213-5716
Email: neli.ulrich@hci.utah.edu

For peer review only - http://bmjopen.bmj.com/site/about/guidelines.xhtml

Abstract

Purpose: The overarching goal of the FOCUS (Biomarkers related to folate-dependent one-carbon metabolism in colorectal cancer recurrence and survival) Consortium is to unravel the effect of folate and folate-mediated one-carbon metabolism (FOCM) biomarkers on colorectal cancer (CRC) prognosis to provide clinically relevant advice on folate intake to cancer patients and define future tertiary prevention strategies.

Participants: The FOCUS Consortium is an international, prospective cohort of 2,401 women and men above 18 years of age who were diagnosed with a primary invasive non-metastatic (stage I-III) CRC. The Consortium comprises patients from Austria, two sites from the Netherlands, Germany and two sites from the USA. Patients are recruited after CRC diagnosis and followed at six and twelve months after enrolment. At each time point, sociodemographic data, data on health behavior, and clinical data are collected, blood samples are drawn.

Findings to date: An increased risk of cancer recurrences was observed among patients with higher compared to lower circulating folic acid concentrations. Furthermore, specific folate species within the FOCM pathway were associated with both inflammation and angiogenesis pathways among CRC patients. In addition, higher vitamin B_6 status was associated with better quality of life at six months post treatment.

Future plans: Better insights into the research on associations between folate and FOCM biomarkers and clinical outcomes in CRC patients will facilitate the development of guidelines regarding folate intake in order to provide clinically relevant advice to cancer patients, health professionals involved in patient care, and ultimately further tertiary prevention strategies in the future. The FOCUS Consortium offers an excellent infrastructure for short- and long-term research projects and for combining additional biomarkers and data resulting from the individual cohorts within the next years, e.g. microbiome data, omics- and multi-omics data or CT-quantified body composition data.

Strengths and limitations of this study

- FOCUS is the largest consortium to date addressing the research question of folate and FOCM biomarkers in relation to survival, recurrence, treatment toxicity, and health-related quality of life outcomes in CRC patients
- The cohorts included in the FOCUS Consortium are designed to enable future pooling of data using harmonized and standardized methods to collect data and biospecimens
- The pooled sample size provides sufficient power to investigate subgroup analyses across CRC patients
- Study time point definitions differ between some of the cohorts and have to be adapted for specific projects
- A selection bias for follow-up can arise because it is possible that patients who experience more severe toxicities, worse clinical outcome or health-related quality of life are underrepresented among those completing follow-ups

For peer review only - http://bmjopen.bmj.com/site/about/guidelines.xhtml

Introduction

Among men and women world-wide, colorectal cancer (CRC) accounts for nearly 10% of all incident cancer cases.[1] In Europe, 5-year survival for CRC patients is approximately 55%, with substantial differences by stage.[2 3] The number of CRC patients continues to increase due to implementation of improved screening strategies and/or enhanced treatment modalities.[4] Many cancer patients seek information on what they can do themselves to improve survival - for instance by improving their dietary habits and other lifestyle factors.[5] However, there may be behavioural aspects among individuals diagnosed with CRC, which may be harmful in some cases, such as the use of high-dose nutritional supplements containing synthetic folate. In general, knowledge on short and long-term effects is insufficient to make sound recommendations on use of dietary supplements, in particular folate, to cancer survivors, even though dietary supplements are used by 20%-85% of patients with cancer[6-8], highlighting the importance of thorough evaluation of potential benefits and harms and to support development of evidence-based recommendations on use of dietary supplements to cancer patients.

Folic acid is the synthetic form of the B-vitamin folate and is often used in dietary supplements or fortified foods. However, there is broad agreement that food folate is less bioavailable than folic acid with a median relative bioavailability of 65% (range: 44–80%), an estimate that approximates the 60% value derived from the Dietary Folate Equivalents equation.[9] Folate and folic acid play an important role in one-carbon metabolism, which is a complex series of biochemical reactions essential in nucleotide synthesis, methylation reactions and amino acid homeostasis.[10] One-carbon metabolism refers to a complex network of biochemical reactions linked to nucleotide synthesis and provides methyl groups for DNA, RNA or protein methylation. Thus, one-carbon metabolism is directly controlling processes determining DNA synthesis and integrity, both processes known to be linked to tumor growth.[11] To what extent folic acid supplement use and biomarkers of folate-mediated one-carbon metabolism (FOCM) impact cancer survival and treatment efficacy and toxicity still needs to be clarified.[11 12] Folate and FOCM biomarker deficiencies may increase cancer risk, but high levels, especially of synthetic folic acid, may also be driving factors in carcinogenesis.[6 13] An increasing body of evidence suggests that folate plays a dual role in carcinogenesis, involving both the prevention of early lesions and potential harm once pre-neoplastic or neoplastic lesions have developed.[6 12 14-20]

For peer review only - http://bmjopen.bmj.com/site/about/guidelines.xhtml

The overarching goal of the FOCUS (biomarkers related to FOlate-dependent one-carbon metabolism in Colorectal cancer recUrrence and Survival) Consortium is to study associations between folate and FOCM biomarkers and recurrence and survival in CRC patients. Better insights into these associations will facilitate the development of guidelines regarding folate intake in order to provide clinically relevant advice to cancer patients, health professionals involved in patient care, and ultimately further tertiary prevention strategies in the future. The FOCUS Consortium is a large-scale international consortium with CRC patients from six prospective cohort studies. The primary objectives of the FOCUS Consortium are: (i) to determine possible associations of folate and other FOCM biomarkers at diagnosis with recurrence or survival in non-metastatic (stage I-III) CRC; (ii) to elucidate whether biomarkers related to FOCM are associated with dietary and supplemental intake of these respective nutrients; (iii) to explore whether FOCM biomarkers are associated with treatment toxicity in CRC patients undergoing chemotherapy; and (iv) to collect comprehensive data of patient characteristics at baseline and follow-up including biomarkers of FOCM to establish an unique resource for future scientific research. The FOCUS Consortium is funded by the European Research Area Network (ERA-NET) on Translational Cancer Research (TRANSCAN).

The main purpose of the FOCUS Cohort profile is to (1) inform the scientific community about the FOCUS Consortium, (2) describe the complex methodology of a large consortium, (3) present ongoing studies using this infrastructure as well as (4) advise interested researchers of opportunities for collaboration. This joint research may lead to a better understanding of the role of folate- and FOCM-related mechanisms in the prognosis of CRC and be a precursor for data for future randomized controlled trials, which will be critical for the development of guidelines regarding folate intake among CRC patients.

For peer review only

Cohort description

The FOCUS Consortium is an international, prospective consortium including six cohort studies recruiting women and men age 18 years and older diagnosed with a primary CRC. The FOCUS Consortium is comprised of patients from the ColoCare Study at the University of Heidelberg, Germany (n=298, 12.3%), the ColoCare Study at the Huntsman Cancer Institute in Salt Lake City, Utah, USA (n=80, 3.3%), the ColoCare Study at the Fred Hutchinson Cancer Research Center in Seattle, Washington, USA (n=157, 6.3%), the COLON Study (COlorectal cancer: Longitudinal, Observational study on Nutritional and lifestyle factors that may influence colorectal tumor recurrence, survival and quality of life) at Wageningen University & Research in the Netherlands (n=1,365, 56.1%), the CORSA Study (Colorectal

Cancer Study of Austria) at the Medical University of Vienna in Austria (n=218, 9.0%), and the EnCoRe Study (Energy for life after ColoRectal cancer) at Maastricht University Medical Center+ in the Netherlands (n=317, 13.0%).

In total, n=2,435 CRC patients were considered for the FOCUS Consortium, of which n=34 patients were excluded due to tumor staging being either 0 or IV leading to a total number of n=2,401 stage I-III CRC patients included in further analyses.

Patients were recruited after CRC diagnosis and repeated study measurements were conducted at time of recruitment, and at six and twelve months thereafter. At each study time point, sociodemographic data, data on lifestyle factors (e.g., diet, supplement use, physical activity), and clinical data (e.g., tumor site and stage, cancer treatment, treatment-induced toxicities, recurrence, survival) were collected, and blood samples were drawn (**Figure 1**). All individuals signed informed consent and the Institutional Review Board at each site approved the corresponding study. Below, a more specific description of each included study is provided.

For peer review only

The ColoCare Study

The ColoCare Study (ClinicalTrials.gov identifier: NCT02328677) is an ongoing international, multi-center prospective cohort study among women and men newly diagnosed with a primary invasive CRC, with the goal to investigate predictors of cancer recurrence, survival, treatment toxicities and health-related quality of life.[4 21] Three ColoCare Consortium sites participate in the FOCUS Consortium: the Fred Hutchinson Cancer Research Center in Seattle (Washington, USA), and the University Hospital Heidelberg, Heidelberg (Germany) as well as the Huntsman Cancer Institute in Salt Lake City (Utah, USA). Patients are enrolled prior to undergoing CRC surgery according to the following inclusion criteria: individuals who are 18 years of age and older, newly diagnosed (i.e., non-recurrent) with invasive CRC. Blood draws and other biospecimens are obtained prior to surgery and at regular intervals (e.g., six, twelve, twenty-four months). Questionnaires are administered to assess lifestyle behavior, health-related quality of life, and clinical outcomes such as CRC recurrence, treatment, and treatment symptoms at each study time point. Clinical data are obtained through reviews of patient medical records, pathology and imaging reports. Vital status is obtained through medical records, routine follow-up mailings, periodic requests for outside medical records, and state or national cancer and death registries. The Heidelberg ColoCare Study was approved by the Ethics Committee of the Medical Faculty of Heidelberg (approval no. S-310/2001 and S-134/2016). The ColoCare Study at the Huntsman Cancer Institute and

For peer review only - http://bmjopen.bmj.com/site/about/guidelines.xhtml

the Fred Hutchinson Cancer Research Center were approved by the respective IRBs (#77147 and #6407).

The COLON Study

The COLON Study (ClinicalTrials.gov identifier: NCT03191110) started in 2010 and is an ongoing, multicenter prospective cohort study specifically designed to assess associations between nutrition, lifestyle, and dietary supplement use with quality of life, CRC recurrence and survival among CRC patients (stages I-IV).[22] Persons with a history of CRC or (partial) bowel resection, chronic inflammatory bowel disease, or a known hereditary CRC syndrome are excluded from the study. Patients are recruited from eleven regional and academic hospitals prior to surgery. Individuals donate blood samples and provide information on diet, lifestyle, and dietary supplement use at CRC diagnosis, i.e. baseline, and at six, twelve (chemotherapy patients only), twenty-four and sixty months after diagnosis. Clinical data are collected through review of medical records (treatment-induced toxicity) or through linkage with the Dutch ColoRectal Audit (DCRA). Mortality data (i.e. death or alive and date of death) are retrieved from the Municipal Personal Records Database. Recurrence data have been retrieved in collaboration with the Netherlands Cancer Registry. Ethical approval for the study was granted by the Committee on Research involving Human Subjects, region Arnhem-Nijmegen under file number 2009/349.

The CORSA Study

CORSA is an ongoing case-control study of women and men recruiting CRC patients, patients with high and low risk adenomas and population-based colonoscopy negative controls, with an age range between 30 and 90 years. Since 2003, more than 13,500 participants have been recruited across nine sites in Austria. The multicentre recruitment within CORSA follows standardized protocols resulting in consistent data from all recruitment sites. These sites include the Medical University of Vienna (Department of Surgery), two hospitals in Vienna (Clinic Favoriten and Clinic Landstraße), and the Hospital Wiener Neustadt in Lower Austria. Furthermore, the recruitment for CORSA was performed in four hospitals in the federal state Burgenland within the population-based screening program "Burgenland PREvention Trial of colorectal cancer DIsease with ImmunologiCal Testing" (B-PREDICT). B-PREDICT is a two-stage screening project where more than 150,000 inhabitants of Burgenland aged between 40 and 80 are invited annually to participate in this program using a Fecal Immunochemical Test (FIT) as an initial screening. FIT-positive

For peer review only - http://bmjopen.bmj.com/site/about/guidelines.xhtml

(≥10 µg hemoglobin / g feces) tested individuals are offered a complete colonoscopy and are asked to participate in CORSA. Biospecimen are collected at each site using harmonized protocols. CORSA participants provide a basic CORSA questionnaire assessing data on body mass index (BMI), smoking history, alcohol consumption, education level, family status, profession, basic dietary habits, and diabetes. Clinical data are abstracted from medical records and processed in a structured database following standardized documentation guidelines and according to the General Data Protection Regulation (GDPR). All subjects gave written informed consent and the study was approved by the institutional review boards: 'Ethikkommission Burgenland' (33/2010), by the ethical review committee of the Medical University of Vienna (1160/2016) and by the "Ethikkommission der Stadt Wien" (EK 06-150VK).

The EnCoRe Study

The EnCoRe Study is a prospective cohort study initiated in 2012 at the Maastricht University Medical Center+ in the Netherlands that focuses on the importance of lifestyle factors for quality of life, recurrence and survival of CRC patients.[23] The EnCoRe Study is registered in the Netherlands Trial Registry for experimental and observational studies (www.trialregister.nl, registration number 7099). Stage I-III CRC patients are enrolled at diagnosis at three hospitals in the South-Eastern region of the Netherlands and followed up until five years after completion of treatment with repeated measurements at diagnosis (pre-treatment), and at six weeks and six, twelve, twenty-four, and sixty months after the end of the initial anti-cancer treatment (i.e., surgery, chemotherapy, radiotherapy). Patients with stage IV CRC, an inability to understand the Dutch language in speech or writing, with comorbidities obstructing successful participation (e.g., Alzheimer disease), or with severe visibility or hearing disorders are excluded from the study. Repeated home visits by trained dieticians are conducted and data are collected amongst others on sociodemographic factors, quality of life, functioning, physical activity, comorbidity, dietary intake, supplement use, and anthropometry. In addition, clinical data are collected from hospital records and blood samples are drawn at all time points. Recurrence data have been retrieved in collaboration with the Netherlands Cancer Registry. Mortality data (i.e. death or alive and date of death) are retrieved from the Municipal Personal Records Database. The EnCoRe Study was approved by the Medical Ethics Committee of the Maastricht University Hospital and Maastricht University (METC 11-3-075).

For peer review only

For peer review only - http://bmjopen.bmj.com/site/about/guidelines.xhtml

The FOCUS Consortium

Sociodemographic and clinical characteristics of the 2,401 participants in the FOCUS Consortium are presented in **Table 1**. The mean age at CRC diagnosis was 65.4 years (standard deviation (SD): 10.2; range: 22 to 93 years), and the majority of participants were male (64.0%). The mean BMI was 27.0 kg/m² (SD: 4.6).

In terms of education status, 35.9% of the patients reported lower education, 28.8% reported intermediate education and 27.3% high education. Most of the patients were married or part of a living community (73.1%). About half of the overall cohort reported to be a former smoker (50.8%), 31.7% were never smokers, and 12.1% were current smokers (5.4% were unknown). Median alcohol intake was 7.9 g/day. In total, 16.9% of the patients reported regular dietary supplementation (i.e., at least once per week during the last four weeks) of folic acid. Over half of all participants (54.6%) reported adherence to the physical activity guidelines of at least 150 minutes per week of moderate-to-vigorous physical activity. Lifestyle characteristics of the FOCUS cohort are presented in **Table 2**.

Regarding clinical characteristics, 57.0% of participants were diagnosed with colon cancer, 33.9% with rectal cancer, and 1.1% with rectosigmoid cancer (8.1% were of unknown tumor subsite). In total, 26.0% were diagnosed with stage I, 28.7% with stage II, and 40.2% with stage III CRC. Approximately 1% of participants were classified with an unspecified cancer stage, as distinction between stage I and II or II and III was not possible, and for 4.4% of the total population the cancer stage was unknown. In total, 89.3% of patients underwent surgery, whereas neoadjuvant chemotherapy was administered to 10.6% of patients and 26.8% received adjuvant chemotherapy. Neoadjuvant radiotherapy was administered to 20.7% of patients and adjuvant radiotherapy in 9.4% of the population.

Patient and public involvement

Patients and the public were not involved in the design, conduct, reporting or dissemination plans of this research.

For peer review only

For peer review only - http://bmjopen.bmj.com/site/about/guidelines.xhtml

Table 1: Sociodemographic and clinical factors of eligible FOCUS Consortium participants at baseline (n=2,401)

	Overall	ColoCare HD	ColoCare FHCRC	ColoCare HCI	COLON	CORSA	EnCoRe
n (%)	2,401 (100)	298 (12.4)	150 (6.3)	80 (3.3)	1,338 (55.7)	218 (9.1)	317 (13.2)
**Age**							
**Mean** y ± SD	65.4 ± 10.2	63.7 ± 12.2	58.3 ± 12.8	61.3 ± 11.2	66.1 ± 8.7	67.3 ± 12.0	66.9 ± 9.3
**Sex**							
**Female** n (%)	864 (36.0)	100 (33.6)	67 (44.7)	32 (40.0)	486 (36.3)	76 (34.9)	103 (32.5)
**Male** n (%)	1,537 (64.0)	198 (66.4)	83 (55.3)	48 (60.0)	852 (63.7)	142 (65.1)	214 (67.5)
**Education**							
**Low** n (%)	861 (35.9)	123 (41.3)	18 (12.0)	17 (21.3)	543 (40.6)	73 (33.5)	87 (27.4)
**Intermediate** n (%)	694 (28.9)	57 (19.1)	71 (47.3)	40 (50.0)	332 (24.8)	73 (33.5)	121 (38.2)
**High** n (%)	658 (27.4)	88 (29.5)	28 (18.7)	8 (10.0)	417 (31.7)	14 (6.4)	103 (32.5)
**Unknown** n (%)	188 (7.8)	30 (10.1)	33 (22.0)	15 (18.7)	46 (3.4)	58 (26.6)	6 (1.9)
**Marital status**							
**Unmarried** n (%)	122 (5.1)	19 (6.4)	15 (10.0)	2 (2.5)	60 (4.5)	10 (4.6)	16 (5.1)
**Married** n (%)	1,738 (72.4)	193 (64.8)	71 (47.3)	50 (62.5)	1,071 (80.0)	113 (51.8)	240 (75.7)
**Divorced/Separated** n (%)	138 (5.7)	24 (8.0)	11 (7.3)	10 (12.5)	64 (4.8)	14 (6.4)	15 (4.7)
**Widowed** n (%)	194 (8.1)	31 (10.4)	7 (4.7)	1 (1.3)	90 (6.7)	26 (11.9)	39 (12.3)
**Living community** n (%)	20 (0.8)	0	13 (8.7)	2 (2.5)	4 (0.3)	1 (0.5)	0
**Unknown** n (%)	189 (8.9)	31 (10.4)	33 (22.0)	15 (18.7)	49 (3.7)	54 (24.8)	7 (2.2)
**Smoking status**							
**Current** n (%)	290 (12.1)	53 (17.8)	8 (5.3)	6 (7.5)	143 (10.7)	38 (17.4)	42 (13.3)
**Former** n (%)	1,219 (50.8)	132 (44.3)	53 (35.32)	21 (26.3)	767 (57.3)	76 (34.9)	170 (53.6)
**Never** n (%)	767 (31.9)	90 (30.2)	56 (37.3)	39 (48.7)	386 (28.9)	97 (44.5)	99 (31.2)
**Unknown** n (%)	125 (5.2)	23 (7.7)	33 (22.0)	14 (17.5)	42 (3.1)	7 (3.2)	6 (1.9)
**Alcohol intake**							
**Mean** g/day ± SD	13.6 ± 17.1	16.1 ± 19.2	8.8 ± 18.5	2.3 ± 10.2	14.0 ± 17.0		12.9 ± 16.5
**Unknown** n (%)	633 (26.4)	208 (69.8)	81 (54.0)	60 (75.0)	54 (4.1)	218 (100)	12 (3.8)
**BMI at diagnosis**							
**Mean** kg/m² ± SD	27.1 ± 4.6	26.6 ± 4.1	28.7 ± 7.3	29.4 ± 7.5	26.6 ± 4.0	27.7 ± 4.4	28.3 ± 4.6
**Unknown** n (%)	42 (1.7)	2 (0.7)	0	14 (17.5)	10 (0.8)	14 (6.4)	2 (0.6)

For peer review only

For peer review only - http://bmjopen.bmj.com/site/about/guidelines.xhtml

	Overall	ColoCare HD	ColoCare FHCRC	ColoCare HCI	COLON	CORSA	EnCoRe
**Tumor site**							
**Colon** n (%)	1,450 (60.4)	140 (57.0)	75 (50.0)	45 (56.3)	867 (64.8)	131 (60.1)	192 (60.6)
**Rectum** n (%)	862 (35.9)	143 (48.0)	60 (40.0)	22 (27.5)	434 (32.4)	78 (35.8)	125 (39.4)
**Rectosigmoid** n (%)	42 (1.7)	15 (5.0)	15 (10.0)	7 (8.7)	0	5 (2.3)	0
**Unknown** n (%)	47 (2.0)	0	0	6 (7.5)	37 (2.8)	4 (1.8)	0
**Tumor stage**							
**I** n (%)	629 (26.2)	76 (25.5)	35 (23.3)	21 (26.3)	326 (24.4)	82 (37.6)	89 (28.1)
**II** n (%)	694 (28.9)	114 (38.3)	49 (32.7)	23 (28.7)	386 (28.9)	52 (23.9)	70 (22.1)
**III** n (%)	973 (40.5)	108 (36.2)	66 (44.0)	36 (45.0)	564 (42.1)	52 (23.9)	147 (46.4)
**Unspecified*** n (%)	16 (0.7)	0	0	0	4 (0.3)	12 (5.5)	0
**Unknown** n (%)	89 (3.7)	0	0	0	58 (4.3)	20 (9.2)	11 (3.5)
**Treatment**							
**Neoadjuvant chemotherapy** n (%)	331 (13.8)	55 (18.5)	47 (31.3)	15 (18.8)	138 (10.3)	14 (6.4)	62 (19.6)
**Unknown** n (%)	58 (2.4)	2 (0.7)	1 (0.7)	10 (12.5)	38 (2.8)	7 (3.2)	0
**Neoadjuvant radiotherapy** n (%)	523 (21.8)	72 (24.2)	48 (32.0)	13 (16.3)	290 (21.7)	14 (6.4)	86 (27.1)
**Unknown** n (%)	58 (2.4)	2 (0.7)	1 (0.7)	10 (12.5)	38 (2.8)	7 (3.2)	0
**Adjuvant chemotherapy** n (%)	682 (28.4)	100 (33.6)	80 (53.3)	32 (40.0)	315 (23.8)	56 (25.7)	99 (31.2)
**Unknown** n (%)	121 (5.0)	17 (5.7)	3 (2.0)	28 (35.0)	63 (4.7)	9 (4.1)	1 (0.3)
**Adjuvant radiotherapy** n (%)	227 (9.5)	188 (63.1)	7 (4.7)	1 (1.3)	16 (1.2)	13 (6.0)	2 (0.6)
**Unknown** n (%)	164 (6.8)	17 (5.7)	3 (2.0)	28 (35.0)	106 (7.9)	9 (4.1)	1 (0.3)
**Surgery** n (%)	2,291 (95.4)	298 (100.0)	149 (99.3)	71 (88.7)	1,290 (96.4)	195 (89.5)	288 (90.9)
**Unknown** n (%)	44 (1.8)	0	0	3 (3.8)	39 (2.9)	1 (0.5)	1 (0.3)

*Distinguishing between stage I and II or II and III not possible

For peer review only - http://bmjopen.bmj.com/site/about/guidelines.xhtml

Table 2: Lifestyle characteristics of eligible FOCUS Consortium participants at baseline (n=2,401)

	Overall	ColoCare HD	ColoCare FHCRC	ColoCare HCI	COLON	CORSA	EnCoRe
n (%)	2,401 (100)	298 (12.4)	150 (6.3)	80 (3.3)	1,338 (55.7)	218 (9.1)	317 (13.2)
**Folic acid supplementation**							
**Yes** n (%)	405 (16.9)	4 (1.3)	9 (5.9)	2 (2.5)	325 (24.3)		65 (20.5)
**Unknown** n (%)	383 (15.9)	27 (9.1)	86 (56.6)	14 (17.5)	38 (2.8)	218 (100)	0
**Vitamin B_{12} supplementation**							
**Yes** n (%)	430 (17.9)	10 (3.4)	8 (5.3)	14 (17.5)	336 (25.1)		62 (19.6)
**Unknown** n (%)	384 (16.0)	27 (9.1)	86 (56.6)	14 (17.5)	39 (2.9)	218 (100)	0
**Vitamin B_6 supplementation**							
**Yes** n (%)	77 (3.2)	8 (2.7)	4 (2.6)	4 (5.0)			61 (19.2)
**Unknown** n (%)	1,684 (70.1)	27 (9.1)	86 (56.6)	14 (17.5)	1,339 (100)	218 (100)	0
**Vitamin B_2 supplementation**							
**Yes** n (%)	62 (2.6)	0	2 (1.3)	0			60 (18.9)
**Unknown** n (%)	1,684 (70.1)	27 (9.1)	86 (56.6)	14 (17.5)	1,339 (100)	218 (100)	0
**Dietary intake of folate equivalents**							
**Mean** µg/day ± SD	227.1 ± 94.3	115.1 ± 38.1	149.6 ± 97.5	116.2 ± 112.8	229.0 ± 88.7		276.3 ± 82.2
**Unknown** n (%)	638 (26.5)	208 (69.8)	83 (54.6)	60 (75.0)	57 (4.3)	218 (100)	12 (3.8)
**Dietary intake of vitamin B_{12}**							
**Mean** µg/day ± SD	4.6 ± 2.4	6.2 ± 2.9	7.1 ± 5.8	4.2 ± 2.2	4.3 ± 2.1		4.6 ± 2.0
**Unknown** n (%)	638 (26.5)	208 (69.8)	83 (54.6)	60 (75.0)	57 (4.3)	218 (100)	12 (3.8)
**Dietary intake of vitamin B_6**							
**Mean** mg/day ± SD	1.5 ± 0.5	1.7 ± 0.5	2.0 ± 0.7	1.5 ± 0.7	1.5 ± 0.5		1.8 ± 0.5
**Unknown** n (%)	638 (26.5)	208 (69.8)	83 (54.6)	60 (75.0)	57 (4.3)	218 (100)	12 (3.8)
**Dietary intake of vitamin B_2**							
**Mean** mg/day ± SD	1.4 ± 0.5	1.5 ± 0.5	2.2 ± 1.0	1.6 ± 0.8	1.3 ± 0.4		1.4 ± 0.5
**Unknown** n (%)	638 (26.5)	208 (69.8)	83 (54.6)	60 (75.0)	57 (4.3)	218 (100)	12 (3.8)
**Total energy intake**							
**Mean** kcal/day ± SD	1,941.8 ± 593.6	2,330.9 ± 714.0	1835.0 ± 775.7	1,398.5 ± 597.8	1,856.9 ± 517.2		2,243.7 ± 651.0
**Unknown** n (%)	638 (26.5)	208 (69.8)	83 (54.6)	60 (75.0)	57 (4.3)	218 (100)	12 (3.8)
**Adherence to physical activity guidelines***							

For peer review only - http://bmjopen.bmj.com/site/about/guidelines.xhtml

**Yes** n (%)	1,310 (54.5)	84 (28.2)	35 (23.0)	20 (25.0)	941 (70.3)		230 (72.6)
**Unknown** n (%)	370 (15.4)	33 (11.1)	53 (34.9)	15 (18.7)	45 (3.4)	218 (100)	6 (1.9)

*Self-reported engagement in at least 150 minutes per week of moderate-to-vigorous physical activity

For peer review only

Cohort follow-up

At each study site, collection of biospecimen, clinical, demographic, questionnaire and anthropometric data occurred at baseline, and at six and twelve months following recruitment. Baseline measurements in CORSA, COLON and EnCoRe have been performed at diagnosis (preferably prior to any cancer treatment) while in the ColoCare Study such measurements have been done prior to surgery (i.e. neoadjuvant treatment might have been applied prior to baseline blood and data collection).

Blood samples at baseline were collected from n=2,132 (88.8%) study participants. N=1,537 (64.0%) participants donated blood at the 6 month and n=614 (25.6%) at the 12 month follow-up time point. Additionally, n=251 (10.5%) EnCoRe patients provided blood samples at 6 weeks post-treatment, a subset specifically of interest at the Maastricht study site.

Data collection

Harmonized baseline and follow-up time points are summarized in **Table 3** and are briefly described below.

Lifestyle and demographic data

ColoCare questionnaires and standardized Food Frequency Questionnaires (FFQ) are used to assess intake of dietary supplements and medication, smoking, dietary intake and other health behaviors at each study time point. In COLON, patients provide information on diet, lifestyle, and supplement use via COLON questionnaires and FFQs. Patients from the CORSA Study are requested to complete a questionnaire assessing anthropometric and demographic factors. Patients enrolled in EnCoRe receive repeated home visits by trained dieticians, where extensive measurements are performed that include assessment of demographic data, physical activity (both questionnaire- and accelerometry-based) and smoking behavior (questionnaire data), dietary intake (FFQs at diagnosis and 7-day food diaries at follow-up measurements), supplement use (registered by dieticians), and anthropometry measurements.

For peer review only

Table 3: Variables available at baseline, and at 6 and 12 month follow-up within the FOCUS Consortium.

Category	Variables	Baseline	6m	12m
**Demographics**	Age	x	x	x
	Gender	x		
	Highest education	x		
	Social status	x		
	Height	x		
	Weight	x	x	x
	BMI	x	x	x
	Smoking status	x	x	x
	Smoking duration	x	x	x
	Smoking pack years	x	x	x
	Menopausal status	x		
	Postmenopausal hormone use	x		
	Race	x		
**Cancer characteristics**	Cancer site	x		
	Cancer stage	x		
	TNM classification	x		
**Treatment**	Preoperative chemotherapy	x		
	Preoperative radiotherapy	x		
	Surgery	x	x	
	Postoperative chemotherapy		x	x
	Postoperative radiotherapy		x	x
**Supplement intake**	Folic acid supplements	x	x	x
	Vitamin B_2 supplements	x	x	x
	Vitamin B_6 supplements	x	x	x
	Vitamin B_{12} supplements	x	x	x
	Vitamin A supplements	x	x	x
	Vitamin C supplements	x	x	x
	Vitamin D supplements	x	x	x
**Supplement intake**	Vitamin E supplements	x	x	x
	Calcium supplements	x	x	x
	Magnesium supplements	x	x	x
	Iron supplements	x	x	x
	Multivitamins	x	x	x
**Dietary nutrients**	Folate equivalents	x	x	x
	Vitamin B_2	x	x	x
	Vitamin B_6	x	x	x
	Vitamin B_{12}	x	x	x
	Vitamin A	x	x	x
	Vitamin C	x	x	x
	Vitamin D	x	x	x
	Vitamin E	x	x	x
	Total protein	x	x	x
	Total fat	x	x	x
	Total carbohydrate	x	x	x

For peer review only - http://bmjopen.bmj.com/site/about/guidelines.xhtml

**Dietary nutrients**	Fibre	x	x	x
	Saturated fatty acids	x	x	x
	Monounsaturated fatty acids	x	x	x
	Polyunsaturated fatty acids	x	x	x
	Alcohol	x	x	x
	Total energy	x	x	x
**Physical activity**	Light physical activity	x	x	x
	Moderate physical activity	x	x	x
	Vigorous physical activity	x	x	x
	Adherence to physical activity guidelines	x	x	x
**Medical history**	Diabetes mellitus	x	x	x
	Asthma, Chronical Bronchitis, COPD, Emphysema	x		
	Heart attack, Heart Failure	x		
	Hypertension	x		
	Stroke	x		
	Ulcer of Stomach or Duodenum	x		
	Hypothyroidism/Hyperthyroidism	x		
	Systemic Lupus Erythematosus	x		
	Inflammatory Bowel Disease (Crohn's disease, ulcerative colitis)	x		
	Familial Adenomatous Polyposis	x		
**Medical history**	Lynch Syndrome (hereditary nonpolyposis colorectal cancer)	x		
**Regular medication use**	Aspirin	x	x	x
	NSAID	x	x	x
	Ibuprofen	x	x	x
	Naproxen	x	x	x
	Celecoxib/Etoricoxib	x	x	x
**Health-related quality of life**	EORTC QLQ-C30	x	x	x
	EORTC QLQ-CR29	x	x	x
	EORTC QLQ-CIPN20	x	x	x
**General information**	Date of questionnaire completion	x	x	x
	Date of blood collection	x	x	x
	Freeze-thaw cycles of blood samples	x	x	x
	Hemolysis	x	x	x
	Time between blood draw and processing/storage	x	x	x

NSAID=non-steroidal anti-inflammatory drug; EORTC=European Organization for Research and Treatment of Cancer; QLQ=quality of life questionnaire; C30=core 30-items; CR29=colon/rectum 29-items; CIPN20=chemotherapy-induced polyneuropathy 20-items.

306

For peer review only

Clinical data and outcomes - Medical chart abstraction

All cancers and medical diagnoses are classified according to ICD-10 (International Classification of Diseases, Tenth Revision) codes. Details on CRC treatment, including type of treatment regimens and treatment toxicity, are abstracted via medical record for all cohorts. Detailed information on the primary outcomes of interest, CRC recurrence and survival, are ascertained through reviews of medical records, pathology reports, and imaging reports documenting the diagnosis of a recurrence. Data on recurrence and vital status is supplemented from the clinical cancer registries and survival data is verified by the inhabitant registries that exist at all study sites. For COLON and EnCoRe, all data on recurrence are retrieved in collaboration with the Netherlands Cancer Registry. Moreover, survival data for CORSA participants was obtained by the Main Association of Austrian Social Insurance Carriers as well as from Statistics Austria.

Patient-reported outcomes

Health-related quality of life is assessed by the validated and widely used cancer-specific 30-item core questionnaire QLQ-C30 and the 29-item CRC module QLQ-CR29, developed by the European Organization for Research and Treatment of Cancer (EORTC).[24] Chemotherapy-induced peripheral neuropathy (CIPN), which is a common toxicity in CRC patients, is measured by the EORTC QLQ-CIPN20.[25] Patient-reported outcomes are available for ColoCare Heidelberg, COLON and EnCoRe.

Biomarkers of FOCM

Blood is processed in identical settings across all study centers. Plasma (CORSA, COLON and EnCoRe) and serum (for the three ColoCare Study sites) samples were collected and immediately centrifuged, aliquoted, and stored at -80°C.

All biological analyses were performed at BEVITAL AS (Bergen, Norway, http://www.bevital.no), which carried out metabolic profiling of biomarkers allocated to seven complementary analytical platforms. Apart from analyses of microbiological active folate[26] and vitamin B_{12}[27]), all analyses were based on mass spectrometry. Circulating folate, separate folate species, 5-methyltetrahydrofolate (the main form in plasma), folic acid (folate in supplements), 4a-OH 5-methyltetrahydrofolate (the primary oxidation form of circulating folate), folate catabolites (p-aminobenzoylglutamate and acetyl p-aminobenzoylglutamate)[28], B_6, B_1 and B_3 vitamins, kynurenines, cotinine, trans-3'-hydroxycotinine, trigonelline[29], choline and its metabolites, creatinine, methylhistidines, arginine and methylated arginines[30]

For peer review only

For peer review only - http://bmjopen.bmj.com/site/about/guidelines.xhtml

were analyzed by liquid chromatography-tandem mass spectroscopy, whereas other amino acids, in addition to total homocysteine, total cysteine and methylmalonic acid were analyzed by gass chromatography-tandem mass spectroscopy.[31] C-reactive protein (hsCRP) and cystatin C and its variants were measured by MALDI-TOF mass spectroscopy.[32]

A comprehensive overview of the panel of biomarkers measured in blood samples of patients enrolled in the FOCUS Consortium is provided in **Table 4**.

Findings to date

The FOCUS Consortium provides a unique opportunity to conduct comprehensive research on folate and FOCM biomarkers status in the tertiary prevention of CRC using data collected both at diagnosis and during standardized follow-up time points. This well-characterized study design provides sufficient statistical power to discern prospective associations with relevant clinical outcomes, including CRC recurrence and survival within relevant subgroups.

Key findings and publications

We investigated associations of circulating concentrations of folate, folic acid, and folate catabolites pABG and apABG, measured around time of diagnosis, with recurrence and survival among 2024 patients diagnosed with stage I-III CRC within the international FOCUS Consortium. We did not observe any statistically significant associations for folate, pABG, and apABG concentrations. However, an increased risk of cancer recurrences was observed among patients with higher compared to lower circulating folic acid concentrations.[33] Further, Kiblawi et al. measured associations between one-carbon metabolites, inflammation- and angiogenesis-related biomarkers in a cross-sectional analysis of 238 patients from the ColoCare Heidelberg cohort. The study showed that specific folate species within the one-carbon metabolism pathway are associated with both inflammation and angiogenesis pathways among CRC patients. In particular, vitamin B6 species, pyridoxal 5'-phosphate (PLP), pyridoxal (PL) and pyridoxic acid (PA), were inversely associated with inflammatory biomarkers C-reactive protein (CRP), serum amyloid A (SAA), IL-6 and IL-8. Thiamine and thiamine monophosphate were inversely correlated with the CRP and IL-6. In addition, positive correlations of PA, PL and PLP with angiogenesis biomarker VEGF-D were observed. Our findings reinforce the notion that B vitamins involved in the one-carbon metabolism may be correlated with carcinogenic processes.[34] This and further research will support the evidence base needed for the development of dietary guidelines for CRC patients.

For peer review only - http://bmjopen.bmj.com/site/about/guidelines.xhtml

Table 4: Measured metabolites and biomarkers within the FOCUS Consortium

Folate and one-carbon metabolites	Abbreviation	Description
**Anthranilic acid**	AA	Tryptophan metabolite
**Asymmetric dimethylarginine**	ADMA	Inhibitor of nitric oxide synthase
**α-Ketoglutaric acid**	aKG	Keto acid of the Krebs cycle
**Alanine**	Ala	Amino acid
**Acetamidobenzoylglutamate**	apABG	Folate catabolite
**Arginine**	Arg	Amino acid
**Asparagine**	Asn	Amino acid
**Aspartic acid**	Asp	Amino acid
**Betaine**	Betaine	Methyl donor
**Choline**	Choline	Methyl donor
**C-reactive protein**	hsCRP	Inflammatory marker
**Cystatin C-desS**	CnCds	Cystatin C isoform
**Cystatin C-desSSP**	CnDcssp	Cystatin C isoform
**Cystatin C**	CnCn	Marker of kidney function
**3Pro-OH cystatin C**	CnCo	Cystatin C isoform
**3Pro-OH cystatin C-desS**	CnCods	Cystatin C isoform
**Total concentration of detected cystatin C isoforms**	CnCt	Marker of kidney function
**Cobalamin**	B_{12}	Vitamin B_{12}
**Cotinine**	Cot	Nicotine metabolite
**Creatinine**	Creat	Marker of kidney function
**C-reactive protein**	CRP	Inflammation
**Cystathionine**	Cysta	Thioether, transsulfuration intermediate
**Dimethylglycine**	DMG	Betaine metabolite
**Folic acid**	B_9	Synthetic form of folate
**Flavin mononucleotide**	FMN	B_2 vitamin
**5-Formyl-tetrahydrofolate**	fTHF	Folate species
**Glutamine**	Gln	Amino acid
**Glutamic acid**	Glu	Amino acid
**Glycine**	Gly	Amino acid
**3-Hydroxyanthranilic acid**	HAA	Tryptophan metabolite
**Homoarginine**	hArg	Amino acid
**Histidine**	Hist	Tryptophan metabolite
**3-Hydroxykynurenine**	HK	Tryptophan metabolite
**4-Alfa-hydroxy-5-methyl-THF**	hmTHF	Folate oxidation product
**Isoleucine**	Ile	Amino acid
**Kynurenic acid**	KA	Tryptophan metabolite
**Kynurenine**	Kyn	Tryptophan metabolite
**Leucine**	Leu	Amino acid
**Lysine**	Lys	Amino acid
**3-Methylhistidine (3-MH)**	m3His	Marker of muscle degradation and meat intake
**1-Methylhistidine (1-MH)**	m1His	Marker of muscle degradation and meat intake
**Methionine**	Met	Amino acid
**Methionine sulfoxide**	MetSo	Oxidation product of Met
**Methylmalonic acid**	MMA	Marker of B_{12} status
**N1-methylnicotinamide**	mNAM	B_3 vitamin
**5-Methyl-tetrahydrofolate**	mTHF	Folate species

For peer review only - http://bmjopen.bmj.com/site/about/guidelines.xhtml

**Nicotinic acid**	NA	B_3 vitamin
**Nicotinamide**	NAM	B_3 vitamin
**Neopterin**	Neopt	Inflammatory marker
**Trans-3'-hydroxycotinine**	OHCot	Nicotine metabolite
**Ornithine**	Orn	Amino acid
**4-Pyridoxic acid**	PA	Vitamin B_6 catabolite
**Para-aminobenzoylglutamate**	pABG	Folate catabolite
**Phenylalanine**	Phe	Amino acid
**Picolinic acid**	Pic	Tryptophan metabolite
**Pyridoxal**	PL	B_6 vitamin
**Pyridoxal 5'-phosphate**	PLP	B_6 vitamin
**Pyridoxine**	PN	Synthetic form of vitamin B_6
**Proline**	Pro	Amino acid
**Quinolinic acid**	QA	Tryptophan metabolite
**Riboflavin**	Ribo	Main circulating B_2 form
**Symmetric dimethylarginine**	SDMA	Marker of renal function
**Serine**	Ser	Amino acid
**Folate**	spFolate	Microbiologically active folate
**Total cysteine**	tCys	Amino acid
**Homocysteine**	tHcy	Marker of folate and B_{12} status
**Thiamine**	Thi	B_1 vitamin
**Threonine**	Thr	Amino acid
**Trimethylamineoxide**	TMAO	Choline metabolite
**Trimethyllysine**	TML	Amino acid
**Thiamine monophosphate**	TMP	B_1 vitamin
**Trigonelline**	Trig	Marker of coffee consumption
**Tryptophan**	Trp	Amino acid
**Valine**	Val	Amino acid
**Leucine**	Leu	Amino acid
**Xanthurenic acid**	XA	Tryptophan metabolite
**Kyn/Trp ratio**	KTR	Marker of immune activation
**HK/XA ratio**		Marker of B_6 status

Further, we investigated circulating concentrations of nine biomarkers related to the B-vitamins folate, riboflavin, vitamin B_6, and cobalamin, measured at diagnosis and six months post-diagnosis, in association with health-related quality of life as assessed by the EORTC QLQ-C30 questionnaire six months post-treatment in three FOCUS cohorts (ColoCare Heidelberg, EnCoRe and COLON).[35] Higher PLP concentrations were cross-sectionally associated with better physical, role, and social functioning, and reduced fatigue six months post-diagnosis. Higher HKr (3-hydroxykynurenine:(kynurenic acid+xanthurenic acid+3-hydroxyanthranilic acid+anthranilic acid)), an inverse marker of vitamin B_6 status, was cross-sectionally associated with worse global quality of life, and lower physical and role functioning. Dose-response relations were observed for PLP with global quality of life, physical, role, and social functioning. No associations were observed for changes in biomarker concentrations between diagnosis and six months with quality of life outcomes. We

For peer review only

therefore concluded that higher vitamin B_6 status was associated with better quality of life at six months post treatment and that further study is needed to clarify the role of vitamin B_6 in relation to quality of life.

Previous relevant findings from individual cohorts within the consortium

To date, individual cohorts from the FOCUS Consortium have initiated the examination of dietary supplement use and dietary habits over time. Among CRC patients enrolled in the ColoCare Study the proportion of supplement users was found to be highest post-diagnosis (35%).[36] Moreover, within an international investigation including ColoCare participants from multiple sites, Ulrich et al. showed differences in plasma folate concentration between Heidelberg and the US sites, probably reflecting variation in folic acid fortification and supplement use.[13] Furthermore, ColoCare has published data on RECQ helicase expression[37], NTRK3[38], RET[39], tumor-infiltrating lymphocytes and T cell receptor sequences[40], 25-25-hydroxyvitamin D_3[13 41], DNA methylation[42-45], miRNAs[46 47], fecal microbiota[48-50], metabolomics and transcriptomics[51-53], plasma proteins[54], gene expression[55], branched-chain amino acids[56], genetic variants[57], body composition[53 58 59], physical activity[41], and dietary patterns[4] in CRC patients. Within the COLON study, results have been published on body weight trajectories[60], changes in lifestyle[61], 25-hydroxy vitamin D levels[62 63], and inflammation markers[64] over time, as well as vitamin D[64], calcium or magnesium intake[65], physical activity[66], inflammation[67 68], skeletal muscle mass[69]/ density[70] and other measures of body composition[71] in relation to cancer recurrence, survival, or physical functioning or fatigue. Moreover, in a subset of patients undergoing chemotherapy, dietary factors in relation to chronic CIPN[72 73] as well as chemosensory perception and food preferences[74] were studied. The Austrian CORSA Study has published results on genomic data[75-77], telomere length[78], DNA repair processes[79], tumor autoantibodies[80] as well as metabolomics.[33 81] To date, publications from the EnCoRe Study have reported on associations of physical activity and sedentary behaviour[82-85], adherence to lifestyle guidelines[86], and parameters of body composition[87 88] measured through CT scans with quality of life, functioning and fatigue in CRC survivors. Recently, longitudinal associations between supplement use and fatigue were investigated from diagnosis to 2 years post-CRC treatment. No overall association between supplement use and fatigue was found but results suggest that increased levels of fatigue may be a reason for the use of supplements among CRC survivors.[89] Higher concentrations of 25OHD3 were longitudinally associated with better global quality of life and less fatigue in colorectal cancer survivors within EnCoRe suggesting a potential beneficial role of vitamin D

For peer review only

For peer review only - http://bmjopen.bmj.com/site/about/guidelines.xhtml

in colorectal cancer survivors.[35] In a mixed-method study using data of the EnCoRe study, colorectal cancer (treatment) related health and functioning problems negatively impacted the ability of nearly 1 in 5 long-term CRC survivors to participate in everyday life situations and their satisfaction with participation.[90] The validity of the food frequency questionnaire for measuring dietary intake among survivors of colorectal cancer within the EnCoRe study appeared to be moderate to good for most nutrients and food groups, relative to a 7-day dietary record.[91] Prediction models that we developed for estimating 1-year risk of low health-related quality of life in seven domains in colorectal cancer survivors performed well when externally validated among survivors within the EnCoRe and COLON studies.[92]

Future plans

The consortium specified a comprehensive manuscript list of future projects using data from the FOCUS Consortium. Some selected projects are described below:

a) Recently, the investigation of longitudinal associations of adherence to the dietary World Cancer Research Fund/American Institute for Cancer Research (WCRF/AICR) and Dutch Healthy Diet (DHD) recommendations with plasma kynurenine levels in colorectal cancer survivors after treatment has been finalized and the corresponding manuscript is under review at an international journal.

b) Further, near-term future plans include the investigation of (1) biomarkers related to FOCM and associations with folate intake (from diet and supplements); (2) associations between FOCM biomarkers such as vitamin B12 and tryptophan and recurrence, survival, and patient-reported outcomes in CRC; (3) the impact of folate status (FOCM biomarkers and diet/supplements) on treatment toxicity in patients treated with 5-FU modifiers; (4) the interaction between biomarkers related to FOCM and polymorphisms in FOCM-related genes in relation to CRC prognosis (recurrence & survival); (5) prognosis (disease-free and overall survival) in stage I-III CRC and associations with dietary and supplement use at diagnosis and changes during and after treatment; (6) FOCM-related biomarkers and their association with body composition in stage I to III CRC patients; (7) associations between folate status (FOCM biomarkers and diet/supplement use) and recurrence, survival and patient-reported outcomes in young-onset CRC.

c) Long-term plans include the combination of additional biomarkers measured by the individual cohorts within the next years (e.g. microbiome data, omics- and multi-omics data etc.).

For peer review only - http://bmjopen.bmj.com/site/about/guidelines.xhtml

Strengths and limitations

This is the largest consortium to date addressing the research question of folate and FOCM biomarkers in relation to survival, recurrence, treatment toxicity, and health-related quality of life outcomes in CRC patients. The cohorts included in the FOCUS Consortium are designed to enable future pooling of data.[22] For that reason, methodologies, time points and measurement instruments generally overlap, with each study presenting unique features such as additional blood collection at the 6-week follow-up time point within the EnCoRe Study and blood draws during chemotherapy within the COLON Study. The pooled sample size provides sufficient power to investigate subgroup analyses across CRC patients, e.g., within groups of patients who underwent 5-floururacil based chemotherapy or stratified by disease stage. Furthermore, the assessment of biomarkers related to FOCM are conducted at a single, state-of-the art laboratory and the biological materials are processed and stored according to standardized operation protocols across all study sites, enabling precise and accurate measurements of FOCM biomarkers. Another advantage of this cohort is the ability to include a time-varying exposure on dietary supplement intake for future studies to consider. The study population is predominantly based on European cohorts (90.4%) in countries that have not implemented mandatory folic acid fortification. This enables us to study a population where dietary intake and dietary supplement use determine differences in folate status, yielding information of direct relevance to cancer patients. However, the generalizability of results to populations that have introduced folic acid fortification, including the United States, might therefore also be limited.[93] Performing sensitivity analyses by excluding countries without folic acid fortification (e.g. Germany) or investigating analyses separately for Germany and the US might help to address differences in fortification status. Moreover, patients were predominantly White, thus, it is not possible to address racial and ethnic minorities. Ethnicity/race is an important determinant of folate status and metabolism may be different between African Americans and Hispanics[94], thus, recommendations should be limited to this current population. Future studies are warranted in diverse populations and compared with the FOCUS cohort.

Another limitation of the consortium includes differences in collection strategies of the baseline study time point across included studies: CORSA, COLON and EnCoRe are recruiting CRC patients at diagnosis, preferably prior to any cancer treatment while ColoCare is recruiting patients prior to surgery (i.e. neoadjuvant treatment might have occurred prior to baseline blood and data collection). Nevertheless, the ColoCare protocol requires that no blood draw occurs within 2 weeks of neoadjuvant or adjuvant chemotherapy, limiting the

For peer review only - http://bmjopen.bmj.com/site/about/guidelines.xhtml

influence of ongoing treatments on blood biomarkers. In some cohorts, the timing with respect to adjuvant chemotherapy is less clearly defined and this will be carefully considered in sensitivity analyses. Furthermore, survivor bias – a type of selection bias – may be introduced as some patients died or did not complete follow-up questionnaires or did not provide blood samples. It is possible that patients who experience more severe toxicities, worse clinical outcomes, or worse health-related quality of life, are underrepresented among those patients who completed follow-up measurements.[4] Cohort studies such as the one presented here generate critical knowledge about preventable causes of disease. However, selection bias may affect estimates. This is particularly true for non-participation at follow-up that may depend on both the exposure and outcome. Within a review, Nohr et al. showed a range of methods to quantify and adjust for selection bias. Even with limited data on nonparticipants and those lost to follow up, it is possible to examine how effect estimates in a specific study may be biased by selection.[95] The likelihood for reverse causation is small in this prospective cohort, as the exposure measurements (blood folate levels and intake through diet/supplements) were collected before the outcome (survival, recurrence, and quality of life) occurred. Therefore, these outcomes are unlikely to have influenced the exposure measurements. Given the robust follow-up in these cohorts for outcomes and data availability, future studies will be able to consider key confounders as well as predictors of recurrence and survival.

Collaboration

A substantial amount of time has been spent in creating the harmonized dataset of baseline variables from cohorts within the FOCUS Consortium, with follow-up data collection and FOCM biomarker analyses. Any person interested in collaborating, learning about the FOCUS Consortium or in getting access to FOCUS data and in-depth analyses can contact the coordinating study PIs Prof. Andrea Gsur (andrea.gsur@meduniwien.ac.at) and Prof. Cornelia Ulrich (neli.ulrich@hci.utah.edu). Requests for data will be discussed and decided by all PIs and will require a Data Transfer Agreement.

For peer review only - http://bmjopen.bmj.com/site/about/guidelines.xhtml

Acknowledgements

ColoCare Heidelberg: We thank all ColoCare study participants, the liquid biobank of the National Center for Tumor Diseases for storage of the blood samples according to the SOPs of the Biomaterialbank Heidelberg (BMBH), Michaela Magenreuter, Maria Thomalla-Starzl, Mareen, Dupovac, Marzena Knyssok-Sypniewski, Lin Zielske, Anett Brendel, Renate Skatula, and Marita Wenzel for laboratory work, Rifraz Farook, Dr. Werner Diehl and Inna Müller for data management and clinical follow-up data collection, Dr. Clare Abbenhardt-Martin, Susanne Jakob, Judith Kammer for patient recruitment and follow-up, and Dr. Petra Schrotz-King and Dr. Jürgen Boehm for study coordination.

ColoCare Huntsman Cancer Institute: We thank all ColoCare study participants, the biorepository at Huntsman Cancer Institute for storage of the blood samples according to standardized SOPs, and Christy Warby for laboratory work. We thank Tyler Farr, Sarah Fischbuch and Debra Ma for data management and clinical follow-up data collection. We are very grateful for patient recruitment and follow-up by Elisa Santori, Hanna Omar-Payne and Marissa Grande, and Dr. Jürgen Boehm for study coordination.

ColoCare Fred Hutchinson Cancer Research Center: We thank all ColoCare study participants, the biorepository at Fred Hutchinson Cancer Research Center for storage of the blood samples according to standardized SOPs and the laboratory team for sample processing. We thank the ColoCare team for patient recruitment and follow-up and data management.

EnCoRe: We would like to thank all participants of the EnCoRe study and the health professionals in the three hospitals involved in the recruitment of participants of the study: Maastricht University Medical Center+, VieCuri Medical Center, and Zuyderland Medical Center. We would also like to thank the MEMIC center for data and information management for facilitating the logistic processes and data management of our study. Finally, we would like to thank the research dieticians and research assistant who are responsible for patient inclusion and follow-up, performing home visits, as well as data collection and processing.

CORSA: We kindly thank all individuals who agreed to participate in the CORSA study. Furthermore, we thank Thomas Bachleitner-Hofmann, Michael M. Bergmann, Judith Karner-Hanusch and Anton Stift (Department of Surgery, Medical University of Vienna), Karl Mach, Azita Deutinger-Permoon (KRAGES, Austria), and their co-workers for supporting CORSA recruitment. In addition, we would like to acknowledge Elisabeth Feik, Philipp Hofer and Cornelia Zöchmeister, for patient recruitment, sample processing, and follow-up.

For peer review only - http://bmjopen.bmj.com/site/about/guidelines.xhtml

COLON: The authors would like to thank the COLON participants, COLON investigators at Wageningen University & Research and the involved co-workers in the participating hospitals.

Contributors

BG, MW, EK, PMU, CMU, AG, ABU, NH: conceived of the project, developed the overall research plan, and provided study oversight; BG, EHvR, ANH, SB, AJMRG, AU, JO, JLK, VD, RK, TG, TL, GK, TK, DEK, FJBvD, MJLB, AB, CIL, WMG, KV: conducted hands-on experiments and data collection; BG, EHvR, ANH, SB, AJMRG, AU, JO, JLK, VD, RK, TG, TL, GK, TK, DEK, FJBvD, CIL, WMG, KV, CMU: provided essential reagents or essential materials, databases, and so forth, necessary for the research; BG, EHvR, MJLB, AB, ANH, VD, SB: analyzed the data or performed the statistical analysis and made a major contribution to writing the paper; BG, MS, EK, PMU, ABU, MW, AG, CMU: had primary responsibility for the final content; all authors: reviewed the manuscript critically, provided feedback, and read and approved the final manuscript.

Funding

The FOCUS Consortium is an ERA-NET (European Research Area Network) on Translational Cancer Research (TRANSCAN) project funded by the local research funding agencies (German Ministry of Education and Research, Germany, 01KT1503; Dutch Cancer Society with grant number UW2014-6877, the Netherlands; FWF Austrian Science Fund, Austria (API02104FW); Research Council Norway/Norwegian Cancer Society (RNC), Norway). The COLON study is further sponsored by Wereld Kanker Onderzoek Fonds, including funds from grant 2014/1179 as part of the World Cancer Research Fund International Regular Grant Programme; Alpe d'Huzes/Dutch Cancer Society (UM 2012-5653, UW 2013-5927, UW 2015-7946); and ERA-NET on Translational Cancer Research (TRANSCAN/Dutch Cancer Society: UW2013-6397 and the Netherlands Organization for Health Research and Development (ZonMw), the Netherlands). The EnCoRe study was supported by grants from the Stichting Alpe d'HuZes within the research program 'Leven met kanker' of the Dutch Cancer Society (Grant No. UM-2010-4867 and UM-2012-5653), and by grants from Kankeronderzoekfonds Limburg as part of Health Foundation Limburg (Grant No. 00005739), and by a grant from Wereld Kanker Onderzoek Fonds (WKOF), as part of the World Cancer Research Fund International grant programme (grant number 2016/1620).

For peer review only - http://bmjopen.bmj.com/site/about/guidelines.xhtml

Biljana Gigic was funded by the ERA-NET on Translational Cancer Research (TRANSCAN), the German Ministry of Education and Research project 01KT1503, the National Institutes of Health/National Cancer Institute (NHI/NCI) projects R01 CA189184 and U01 CA206110, the Stiftung LebensBlicke, and the Matthias-Lackas Foundations. Eline H. van Roekel was funded by the Wereld Kanker Onderzoek Fonds (WKOF), part of the World Cancer Research Fund International grant programme (grant number 2016/1620). Martijn Bours was funded by Alpe d'HuZes within the research program 'Leven met kanker' of the Dutch Cancer Society (Grant No. UM-2012-5653). Dieuwertje E. Kok is supported by a Veni grant of the Netherlands Organisation for Scientific Research (grant no. 016.Veni.188.082). Andreana N. Holowatyj was supported by the National Institutes of Health under Ruth L. Kirschstein National Research Service Award T32 HG008962 from the National Human Genome Research Institute. Nina Habermann was funded by the TRANSCAN project 01KT1512, Cornelia M. Ulrich and Jennifer Ose were funded by the Huntsman Cancer Foundation, and NIH/NCI projects R01 CA189184, R01 CA207371, U01 CA206110, U01 CA206110 and P30 CA042014. NIH grants (P30 CA15704, U01 CA152756, R01 CA194663, R01 CA220004, P01 CA077852), Rodger C. Haggitt Endowed Chair, R.A.C.E. Charities, Cottrell Family Fund, Listwin Family Foundation, Seattle Translational Tumor Research program, Fred Hutchinson Cancer Research Center (William M. Grady). Stefanie Brezina was funded by the ERA-NET on Translational Cancer Research (TRANSCAN) project FOCUS I2104-B26 and the ERA-NET on Translational Cancer Research (TRANSCAN) project MetaboCCC I 1578 – B19. J.L. Koole was supported by Kankeronderzoekfonds Limburg as part of Health Foundation Limburg (Grant No. 00005739). Victoria Damerell was funded by the German Ministry of Education and Research project (01KD2101D).

Disclaimer

The funder had no role in the design of the FOCUS consortium, or conduct of the study, collection, management, analysis, and interpretation of the data, preparation, review, or approval of the manuscript, or decision to submit the manuscript for publication.

Competing interests

None declared.

Patient consent for publication

Not applicable.

For peer review only

For peer review only - http://bmjopen.bmj.com/site/about/guidelines.xhtml

Patient and public involvement

Patients and/or the public were not involved in the design, or conduct, or reporting, or dissemination plans of this research**.**

Ethics approval

The Heidelberg ColoCare Study was approved by the Ethics Committee of the Medical Faculty of Heidelberg (approval no. S-310/2001 and S-134/2016). The ColoCare Study at the Huntsman Cancer Institute and the Fred Hutchinson Cancer Research Center were approved by the respective IRBs (#77147 and #6407). For the CORSA study all subjects gave written informed consent and the study was approved by the institutional review boards: 'Ethikkommission Burgenland' (33/2010), by the ethical review committee of the Medical University of Vienna (1160/2016) and by the "Ethikkommission der Stadt Wien" (EK 06-150VK). The EnCoRe Study was approved by the Medical Ethics Committee of the Maastricht University Hospital and Maastricht University (METC 11-3-075).

Data availability

Data described in the manuscript, code book, and analytic code have been generated from European-based consortia and as such are subject to regulations from multiple European countries, which limit our availability to share data. The consortium's funding has ended, and no centralized staff is available to support

data requests. However, the FOCUS PIs have agreed to answer any queries or discuss potential projects with anyone interested in future collaborative research. For further questions please contact colocarestudy_admin@hci.utah.edu.

For peer review only - http://bmjopen.bmj.com/site/about/guidelines.xhtml

References

1. Ferlay J, Colombet M, Soerjomataram I, et al. Estimating the global cancer incidence and mortality in 2018: GLOBOCAN sources and methods. *International journal of cancer Journal international du cancer* 2019;144(8):1941-53. doi: 10.1002/ijc.31937 [published Online First: 2018/10/24]
2. De Angelis R, Sant M, Coleman MP, et al. Cancer survival in Europe 1999-2007 by country and age: results of EUROCARE--5-a population-based study. *The Lancet Oncology* 2014;15(1):23-34. doi: 10.1016/S1470-2045(13)70546-1
3. Ferlay J, Colombet M, Soerjomataram I, et al. Cancer incidence and mortality patterns in Europe: Estimates for 40 countries and 25 major cancers in 2018. *European journal of cancer* 2018;103:356-87. doi: 10.1016/j.ejca.2018.07.005 [published Online First: 2018/08/14]
4. Gigic B, Boeing H, Toth R, et al. Associations Between Dietary Patterns and Longitudinal Quality of Life Changes in Colorectal Cancer Patients: The ColoCare Study. *Nutrition and cancer* 2017:1-10. doi: 10.1080/01635581.2018.1397707 [published Online First: 2017/12/16]
5. Velicer CM, Ulrich CM. Vitamin and mineral supplement use among US adults after cancer diagnosis: a systematic review. *Journal of clinical oncology : official journal of the American Society of Clinical Oncology* 2008;26(4):665-73. doi: 10.1200/JCO.2007.13.5905
6. Ulrich CM, Potter JD. Folate supplementation: too much of a good thing? *Cancer Epidemiol Biomarkers Prev* 2006;15(2):189-93. doi: 10.1158/1055-9965.EPI-152CO [published Online First: 2006/02/24]
7. Jatoi A, Williams B, Nichols F, et al. Is voluntary vitamin and mineral supplementation associated with better outcome in non-small cell lung cancer patients? Results from the Mayo Clinic lung cancer cohort. *Lung Cancer* 2005;49(1):77-84. doi: 10.1016/j.lungcan.2005.01.004 [published Online First: 2005/06/14]
8. Ferrucci LM, McCorkle R, Smith T, et al. Factors related to the use of dietary supplements by cancer survivors. *J Altern Complement Med* 2009;15(6):673-80. doi: 10.1089/acm.2008.0387
9. Caudill MA. Folate bioavailability: implications for establishing dietary recommendations and optimizing status. *The American journal of clinical nutrition* 2010;91(5):1455S-60S. doi: 10.3945/ajcn.2010.28674E [published Online First: 2010/03/12]
10. Ducker GS, Rabinowitz JD. One-Carbon Metabolism in Health and Disease. *Cell Metab* 2017;25(1):27-42. doi: 10.1016/j.cmet.2016.08.009 [published Online First: 2016/09/20]
11. Miller JW, Ulrich CM. Folic acid and cancer--where are we today? *Lancet* 2013;381(9871):974-6. doi: 10.1016/S0140-6736(13)60110-5 [published Online First: 2013/01/29]
12. Kim YI. Folate and colorectal cancer: an evidence-based critical review. *Mol Nutr Food Res* 2007;51(3):267-92. doi: 10.1002/mnfr.200600191 [published Online First: 2007/02/14]
13. Ulrich CM, Toriola AT, Siegel EM, et al. Plasma 25-hydroxyvitamin D3, folate and vitamin B12 biomarkers among international colorectal cancer patients: a pilot study. *J Nutr Sci* 2013;2:e9. doi: 10.1017/jns.2012.28
14. Cole BF, Baron JA, Sandler RS, et al. Folic acid for the prevention of colorectal adenomas: a randomized clinical trial. *Jama* 2007;297(21):2351-9. doi: 10.1001/jama.297.21.2351 [published Online First: 2007/06/07]
15. Lawrance AK, Deng L, Rozen R. Methylenetetrahydrofolate reductase deficiency and low dietary folate reduce tumorigenesis in Apc min/+ mice. *Gut* 2009;58(6):805-11. doi: 10.1136/gut.2007.143107 [published Online First: 2009/01/29]
16. Lindzon GM, Medline A, Sohn KJ, et al. Effect of folic acid supplementation on the progression of colorectal aberrant crypt foci. *Carcinogenesis* 2009;30(9):1536-43. doi: 10.1093/carcin/bgp152 [published Online First: 2009/06/23]
17. Mason JB. Folate, cancer risk, and the Greek god, Proteus: a tale of two chameleons. *Nutrition reviews* 2009;67(4):206-12. doi: 10.1111/j.1753-4887.2009.00190.x [published Online First: 2009/04/02]
18. Ulrich CM, Miller JW. Folate and health: present and future. *Mol Nutr Food Res* 2013;57(4):561. doi: 10.1002/mnfr.201370034 [published Online First: 2013/04/05]
19. Choi JH, Yates Z, Martin C, et al. Gene-Nutrient Interaction between Folate and Dihydrofolate Reductase in Risk for Adenomatous Polyp Occurrence: A Preliminary Report. *J Nutr Sci*

For peer review only

For peer review only - http://bmjopen.bmj.com/site/about/guidelines.xhtml

Vitaminol (Tokyo) 2015;61(6):455-9. doi: 10.3177/jnsv.61.455 [published Online First: 2016/02/16]
20. Sawaengsri H, Wang J, Reginaldo C, et al. High folic acid intake reduces natural killer cell cytotoxicity in aged mice. *J Nutr Biochem* 2016;30:102-7. doi: 10.1016/j.jnutbio.2015.12.006 [published Online First: 2016/03/26]
21. Ulrich CM, Gigic B, Bohm J, et al. The ColoCare Study: A Paradigm of Transdisciplinary Science in Colorectal Cancer Outcomes. *Cancer Epidemiol Biomarkers Prev* 2019;28(3):591-601. doi: 10.1158/1055-9965.EPI-18-0773 [published Online First: 2018/12/14]
22. Winkels RM, Heine-Broring RC, van Zutphen M, et al. The COLON study: Colorectal cancer: Longitudinal, Observational study on Nutritional and lifestyle factors that may influence colorectal tumour recurrence, survival and quality of life. *BMC cancer* 2014;14:374. doi: 10.1186/1471-2407-14-374 [published Online First: 2014/06/03]
23. van Roekel EH, Bours MJ, de Brouwer CP, et al. The applicability of the international classification of functioning, disability, and health to study lifestyle and quality of life of colorectal cancer survivors. *Cancer Epidemiol Biomarkers Prev* 2014;23(7):1394-405. doi: 10.1158/1055-9965.EPI-13-1144 [published Online First: 2014/05/08]
24. Fayers P, Bottomley A, Group EQoL, et al. Quality of life research within the EORTC-the EORTC QLQ-C30. European Organisation for Research and Treatment of Cancer. *European journal of cancer* 2002;38 Suppl 4:S125-33.
25. Postma TJ, Aaronson NK, Heimans JJ, et al. The development of an EORTC quality of life questionnaire to assess chemotherapy-induced peripheral neuropathy: the QLQ-CIPN20. *European journal of cancer* 2005;41(8):1135-9. doi: 10.1016/j.ejca.2005.02.012
26. Molloy AM, Scott JM. Microbiological assay for serum, plasma, and red cell folate using cryopreserved, microtiter plate method. *Methods Enzymol* 1997;281:43-53. [published Online First: 1997/01/01]
27. Kelleher BP, Broin SD. Microbiological assay for vitamin B12 performed in 96-well microtitre plates. *J Clin Pathol* 1991;44(7):592-5. [published Online First: 1991/07/01]
28. Hannisdal R, Ueland PM, Svardal A. Liquid chromatography-tandem mass spectrometry analysis of folate and folate catabolites in human serum. *Clin Chem* 2009;55(6):1147-54. doi: 10.1373/clinchem.2008.114389 [published Online First: 2009/04/11]
29. Midttun O, Hustad S, Ueland PM. Quantitative profiling of biomarkers related to B-vitamin status, tryptophan metabolism and inflammation in human plasma by liquid chromatography/tandem mass spectrometry. *Rapid Commun Mass Spectrom* 2009;23(9):1371-9. doi: 10.1002/rcm.4013 [published Online First: 2009/04/02]
30. Midttun O, Kvalheim G, Ueland PM. High-throughput, low-volume, multianalyte quantification of plasma metabolites related to one-carbon metabolism using HPLC-MS/MS. *Anal Bioanal Chem* 2013;405(6):2009-17. doi: 10.1007/s00216-012-6602-6 [published Online First: 2012/12/13]
31. Midttun O, McCann A, Aarseth O, et al. Combined Measurement of 6 Fat-Soluble Vitamins and 26 Water-Soluble Functional Vitamin Markers and Amino Acids in 50 muL of Serum or Plasma by High-Throughput Mass Spectrometry. *Anal Chem* 2016;88(21):10427-36. doi: 10.1021/acs.analchem.6b02325 [published Online First: 2016/11/02]
32. Meyer K, Ueland PM. Targeted quantification of C-reactive protein and cystatin c and its variants by immuno-MALDI-MS. *Anal Chem* 2014;86(12):5807-14. doi: 10.1021/ac500704y [published Online First: 2014/05/23]
33. Geijsen A, Ulvik A, Gigic B, et al. Circulating Folate and Folic Acid Concentrations: Associations With Colorectal Cancer Recurrence and Survival. *JNCI Cancer Spectr* 2020;4(5):pkaa051. doi: 10.1093/jncics/pkaa051 [published Online First: 2020/11/03]
34. Kiblawi R, Holowatyj AN, Gigic B, et al. One-carbon metabolites, B-vitamins and associations with systemic inflammation and angiogenesis biomarkers among colorectal cancer patients: results from the ColoCare Study. *The British journal of nutrition* 2020:1-32. doi: 10.1017/S0007114520000422 [published Online First: 2020/02/06]
35. Koole JL, Bours MJL, Geijsen A, et al. Circulating B-vitamin biomarkers and B-vitamin supplement use in relation to quality of life in patients with colorectal cancer: results from the FOCUS consortium. *The American journal of clinical nutrition* 2021 doi: 10.1093/ajcn/nqaa422 [published Online First: 2021/03/06]

For peer review only - http://bmjopen.bmj.com/site/about/guidelines.xhtml

36. Schrotz-King P, Dölp K, Paskow MJ, et al. Dietary Supplement Use among German Colorectal Cancer Patients: the ColoCare Study: AACR/MEG Post-GWAS Horizons in Molecular Epidemiology: Digging Deeper into the Environment., 2012.
37. Lao VV, Welcsh P, Luo Y, et al. Altered RECQ Helicase Expression in Sporadic Primary Colorectal Cancers. *Transl Oncol* 2013;6(4):458-69.
38. Luo Y, Kaz AM, Kanngurn S, et al. NTRK3 is a potential tumor suppressor gene commonly inactivated by epigenetic mechanisms in colorectal cancer. *PLoS Genet* 2013;9(7):e1003552. doi: 10.1371/journal.pgen.1003552
39. Luo Y, Tsuchiya KD, Il Park D, et al. RET is a potential tumor suppressor gene in colorectal cancer. *Oncogene* 2013;32(16):2037-47. doi: 10.1038/onc.2012.225
40. Sherwood AM, Emerson RO, Scherer D, et al. Tumor-infiltrating lymphocytes in colorectal tumors display a diversity of T cell receptor sequences that differ from the T cells in adjacent mucosal tissue. *Cancer Immunol Immunother* 2013;62(9):1453-61. doi: 10.1007/s00262-013-1446-2
41. Skender S, Bohm J, Schrotz-King P, et al. Plasma 25-Hydroxyvitamin D3 Levels in Colorectal Cancer Patients and Associations with Physical Activity. *Nutrition and cancer* 2017;69(2):229-37. doi: 10.1080/01635581.2017.1265131
42. Luo Y, Wong CJ, Kaz AM, et al. Differences in DNA methylation signatures reveal multiple pathways of progression from adenoma to colorectal cancer. *Gastroenterology* 2014;147(2):418-29 e8. doi: 10.1053/j.gastro.2014.04.039
43. Barrow TM, Klett H, Toth R, et al. Smoking is associated with hypermethylation of the APC 1A promoter in colorectal cancer: the ColoCare Study. *J Pathol* 2017;243(3):366-75. doi: 10.1002/path.4955 [published Online First: 2017/08/10]
44. Yu M, Carter KT, Makar KW, et al. MethyLight droplet digital PCR for detection and absolute quantification of infrequently methylated alleles. *Epigenetics* 2015;10(9):803-9. doi: 10.1080/15592294.2015.1068490
45. Klett H, Balavarca Y, Toth R, et al. Robust prediction of gene regulation in colorectal cancer tissues from DNA methylation profiles. *Epigenetics* 2018;13(4):386-97. doi: 10.1080/15592294.2018.1460034 [published Online First: 2018/04/27]
46. Ristau J, Staffa J, Schrotz-King P, et al. Suitability of circulating miRNAs as potential prognostic markers in colorectal cancer. *Cancer Epidemiol Biomarkers Prev* 2014;23(12):2632-7. doi: 10.1158/1055-9965.EPI-14-0556
47. Yuan Z, Baker K, Redman MW, et al. Dynamic plasma microRNAs are biomarkers for prognosis and early detection of recurrence in colorectal cancer. *British journal of cancer* 2017;117(8):1202-10. doi: 10.1038/bjc.2017.266 [published Online First: 2017/08/16]
48. Zeller G, Tap J, Voigt AY, et al. Potential of fecal microbiota for early-stage detection of colorectal cancer. *Mol Syst Biol* 2014;10:766. doi: 10.15252/msb.20145645
49. Thomas AM, Manghi P, Asnicar F, et al. Metagenomic analysis of colorectal cancer datasets identifies cross-cohort microbial diagnostic signatures and a link with choline degradation. *Nat Med* 2019;25(4):667-78. doi: 10.1038/s41591-019-0405-7 [published Online First: 2019/04/03]
50. Wirbel J, Pyl PT, Kartal E, et al. Meta-analysis of fecal metagenomes reveals global microbial signatures that are specific for colorectal cancer. *Nat Med* 2019;25(4):679-89. doi: 10.1038/s41591-019-0406-6 [published Online First: 2019/04/03]
51. Liesenfeld DB, Grapov D, Fahrmann JF, et al. Metabolomics and transcriptomics identify pathway differences between visceral and subcutaneous adipose tissue in colorectal cancer patients: the ColoCare study. *The American journal of clinical nutrition* 2015;102(2):433-43. doi: 10.3945/ajcn.114.103804
52. Liesenfeld DB, Habermann N, Toth R, et al. Changes in urinary metabolic profiles of colorectal cancer patients enrolled in a prospective cohort study (ColoCare). *Metabolomics* 2015;11(4):998-1012. doi: 10.1007/s11306-014-0758-3 [published Online First: 2015/08/01]
53. Ose J, Gigic B, Lin T, et al. Multiplatform Urinary Metabolomics Profiling to Discriminate Cachectic from Non-Cachectic Colorectal Cancer Patients: Pilot Results from the ColoCare Study. *Metabolites* 2019;9(9) doi: 10.3390/metabo9090178 [published Online First: 2019/09/11]

For peer review only - http://bmjopen.bmj.com/site/about/guidelines.xhtml

54. Bohm J, Pianka F, Stuttgen N, et al. Discovery of novel plasma proteins as biomarkers for the development of incisional hernias after midline incision in patients with colorectal cancer: The ColoCare study. *Surgery* 2017;161(3):808-17. doi: 10.1016/j.surg.2016.08.025

55. Gong J, Hutter CM, Newcomb PA, et al. Genome-Wide Interaction Analyses between Genetic Variants and Alcohol Consumption and Smoking for Risk of Colorectal Cancer. *PLoS Genet* 2016;12(10):e1006296. doi: 10.1371/journal.pgen.1006296 [published Online First: 2016/10/11]

56. Delphan M, Lin T, Liesenfeld DB, et al. Associations of branched-chain amino acids with parameters of energy balance and survival in colorectal cancer patients: Results from the ColoCare Study. *Metabolomics* 2018;2018(14) doi: 10.1007/s11306-017-1314-8 [published Online First: 2018/05/01]

57. Rosenthal EA, Shirts BH, Amendola LM, et al. Rare loss of function variants in candidate genes and risk of colorectal cancer. *Hum Genet* 2018 doi: 10.1007/s00439-018-1938-4 [published Online First: 2018/09/30]

58. Nattenmueller J, Hoegenauer H, Boehm J, et al. CT-based compartmental quantification of adipose tissue versus body metrics in colorectal cancer patients. *Eur Radiol* 2016;26(11):4131-40. doi: 10.1007/s00330-016-4231-8

59. Ozoya OO, Siegel EM, Srikumar T, et al. Quantitative Assessment of Visceral Obesity and Postoperative Colon Cancer Outcomes. *J Gastrointest Surg* 2017;21(3):534-42. doi: 10.1007/s11605-017-3362-9 [published Online First: 2017/01/20]

60. van Zutphen M, Geelen A, Boshuizen HC, et al. Pre-to-post diagnosis weight trajectories in colorectal cancer patients with non-metastatic disease. *Supportive care in cancer : official journal of the Multinational Association of Supportive Care in Cancer* 2019;27(4):1541-49. doi: 10.1007/s00520-018-4560-z [published Online First: 2018/11/30]

61. van Zutphen M, Boshuizen HC, Kok DE, et al. Colorectal cancer survivors only marginally change their overall lifestyle in the first 2 years following diagnosis. *J Cancer Surviv* 2019;13(6):956-67. doi: 10.1007/s11764-019-00812-7 [published Online First: 2019/10/28]

62. Wesselink E, Bours MJL, de Wilt JHW, et al. Chemotherapy and vitamin D supplement use are determinants of serum 25-hydroxyvitamin D levels during the first six months after colorectal cancer diagnosis. *J Steroid Biochem Mol Biol* 2020;199:105577. doi: 10.1016/j.jsbmb.2020.105577 [published Online First: 2020/01/10]

63. Wesselink E, Kok DE, de Wilt JHW, et al. Sufficient 25-Hydroxyvitamin D Levels 2 Years after Colorectal Cancer Diagnosis are Associated with a Lower Risk of All-cause Mortality. *Cancer Epidemiol Biomarkers Prev* 2021;30(4):765-73. doi: 10.1158/1055-9965.EPI-20-1388 [published Online First: 2021/02/04]

64. Wesselink E, Balvers M, Bours MJL, et al. The association between circulating levels of vitamin D and inflammatory markers in the first 2 years after colorectal cancer diagnosis. *Therap Adv Gastroenterol* 2020;13:1756284820923922. doi: 10.1177/1756284820923922 [published Online First: 2020/06/18]

65. Wesselink E, Kok DE, Bours MJL, et al. Vitamin D, magnesium, calcium, and their interaction in relation to colorectal cancer recurrence and all-cause mortality. *The American journal of clinical nutrition* 2020;111(5):1007-17. doi: 10.1093/ajcn/nqaa049 [published Online First: 2020/03/20]

66. van Zutphen M, Winkels RM, van Duijnhoven FJ, et al. An increase in physical activity after colorectal cancer surgery is associated with improved recovery of physical functioning: a prospective cohort study. *BMC cancer* 2017;17(1):74. doi: 10.1186/s12885-017-3066-2 [published Online First: 2017/01/27]

67. Wesselink E, van Baar H, van Zutphen M, et al. Inflammation Is a Mediating Factor in the Association between Lifestyle and Fatigue in Colorectal Cancer Patients. *Cancers (Basel)* 2020;12(12) doi: 10.3390/cancers12123701 [published Online First: 2020/12/16]

68. Wesselink E, Staritsky LE, van Zutphen M, et al. The association between the adapted dietary inflammatory index and colorectal cancer recurrence and all-cause mortality. *Clin Nutr* 2021;40(6):4436-43. doi: 10.1016/j.clnu.2021.01.004 [published Online First: 2021/01/23]

69. van Baar H, Winkels RM, Brouwer JGM, et al. Associations of Abdominal Skeletal Muscle Mass, Fat Mass, and Mortality among Men and Women with Stage I-III Colorectal Cancer. *Cancer*

For peer review only - http://bmjopen.bmj.com/site/about/guidelines.xhtml

Epidemiol Biomarkers Prev 2020;29(5):956-65. doi: 10.1158/1055-9965.EPI-19-1134 [published Online First: 2020/03/07]

70. van Baar H, Beijer S, Bours MJL, et al. Low radiographic muscle density is associated with lower overall and disease-free survival in early-stage colorectal cancer patients. *J Cancer Res Clin Oncol* 2018;144(11):2139-47. doi: 10.1007/s00432-018-2736-z [published Online First: 2018/08/19]
71. van Baar H, Bours MJL, Beijer S, et al. Body composition and its association with fatigue in the first 2 years after colorectal cancer diagnosis. *J Cancer Surviv* 2021;15(4):597-606. doi: 10.1007/s11764-020-00953-0 [published Online First: 2020/10/18]
72. Winkels RM, van Brakel L, van Baar H, et al. Are Ergothioneine Levels in Blood Associated with Chronic Peripheral Neuropathy in Colorectal Cancer Patients Who Underwent Chemotherapy? *Nutrition and cancer* 2020;72(3):451-59. doi: 10.1080/01635581.2019.1637005 [published Online First: 2019/07/13]
73. Wesselink E, Winkels RM, van Baar H, et al. Dietary Intake of Magnesium or Calcium and Chemotherapy-Induced Peripheral Neuropathy in Colorectal Cancer Patients. *Nutrients* 2018;10(4) doi: 10.3390/nu10040398 [published Online First: 2018/03/24]
74. Postma EM, Kok DE, de Graaf C, et al. Chemosensory perception and food preferences in colorectal cancer patients undergoing adjuvant chemotherapy. *Clin Nutr ESPEN* 2020;40:242-51. doi: 10.1016/j.clnesp.2020.09.012 [published Online First: 2020/11/14]
75. Hofer P, Hagmann M, Brezina S, et al. Bayesian and frequentist analysis of an Austrian genome-wide association study of colorectal cancer and advanced adenomas. *Oncotarget* 2017;8(58):98623-34. doi: 10.18632/oncotarget.21697 [published Online First: 2017/12/13]
76. Schmit SL, Edlund CK, Schumacher FR, et al. Novel Common Genetic Susceptibility Loci for Colorectal Cancer. *Journal of the National Cancer Institute* 2019;111(2):146-57. doi: 10.1093/jnci/djy099 [published Online First: 2018/06/20]
77. Schafmayer C, Harrison JW, Buch S, et al. Genome-wide association analysis of diverticular disease points towards neuromuscular, connective tissue and epithelial pathomechanisms. *Gut* 2019 doi: 10.1136/gutjnl-2018-317619 [published Online First: 2019/01/21]
78. Zochmeister C, Brezina S, Hofer P, et al. Leukocyte telomere length throughout the continuum of colorectal carcinogenesis. *Oncotarget* 2018;9(17):13582-92. doi: 10.18632/oncotarget.24431 [published Online First: 2018/03/24]
79. Jiraskova K, Hughes DJ, Brezina S, et al. Functional Polymorphisms in DNA Repair Genes Are Associated with Sporadic Colorectal Cancer Susceptibility and Clinical Outcome. *Int J Mol Sci* 2018;20(1) doi: 10.3390/ijms20010097 [published Online First: 2018/12/29]
80. Luna Coronell JA, Sergelen K, Hofer P, et al. The Immunome of Colon Cancer: Functional In Silico Analysis of Antigenic Proteins Deduced from IgG Microarray Profiling. *Genomics Proteomics Bioinformatics* 2018;16(1):73-84. doi: 10.1016/j.gpb.2017.10.002 [published Online First: 2018/03/06]
81. Geijsen A, van Roekel EH, van Duijnhoven FJB, et al. Plasma metabolites associated with colorectal cancer stage: Findings from an international consortium. *International journal of cancer Journal international du cancer* 2019 doi: 10.1002/ijc.32666 [published Online First: 2019/09/10]
82. Van Roekel EH, Bours MJ, Breedveld-Peters JJ, et al. Light Physical Activity Is Associated with Quality of Life after Colorectal Cancer. *Med Sci Sports Exerc* 2015;47(12):2493-503. doi: 10.1249/MSS.0000000000000698 [published Online First: 2015/05/15]
83. van Roekel EH, Winkler EA, Bours MJ, et al. Associations of sedentary time and patterns of sedentary time accumulation with health-related quality of life in colorectal cancer survivors. *Prev Med Rep* 2016;4:262-9. doi: 10.1016/j.pmedr.2016.06.022 [published Online First: 2016/07/16]
84. van Roekel EH, Bours MJ, Breedveld-Peters JJ, et al. Modeling how substitution of sedentary behavior with standing or physical activity is associated with health-related quality of life in colorectal cancer survivors. *Cancer causes & control : CCC* 2016;27(4):513-25. doi: 10.1007/s10552-016-0725-6 [published Online First: 2016/02/20]
85. van Roekel EH, Duchateau J, Bours MJL, et al. Longitudinal associations of light-intensity physical activity with quality of life, functioning and fatigue after colorectal cancer. *Quality of life research : an international journal of quality of life aspects of treatment, care and*

For peer review only - http://bmjopen.bmj.com/site/about/guidelines.xhtml

rehabilitation 2020;29(11):2987-98. doi: 10.1007/s11136-020-02566-7 [published Online First: 2020/07/04]
86. Breedveld-Peters JJL, Koole JL, Muller-Schulte E, et al. Colorectal cancers survivors' adherence to lifestyle recommendations and cross-sectional associations with health-related quality of life. *The British journal of nutrition* 2018;120(2):188-97. doi: 10.1017/S0007114518000661 [published Online First: 2018/04/17]
87. van Roekel EH, Bours MJL, Te Molder MEM, et al. Associations of adipose and muscle tissue parameters at colorectal cancer diagnosis with long-term health-related quality of life. *Quality of life research : an international journal of quality of life aspects of treatment, care and rehabilitation* 2017;26(7):1745-59. doi: 10.1007/s11136-017-1539-z [published Online First: 2017/03/21]
88. van Heinsbergen M, Konsten JL, Bours MJL, et al. Preoperative handgrip strength is not associated with complications and health-related quality of life after surgery for colorectal cancer. *Sci Rep* 2020;10(1):13005. doi: 10.1038/s41598-020-69806-1 [published Online First: 2020/08/05]
89. Koole JL, Bours MJL, Breedveld-Peters JJL, et al. Is dietary supplement use longitudinally associated with fatigue in stage I-III colorectal cancer survivors? *Clin Nutr* 2019 doi: 10.1016/j.clnu.2018.12.037 [published Online First: 2019/02/17]
90. Breedveld-Peters JJL, Bours MJL, Cords CI, et al. The impact of participation restrictions on everyday life in long-term colorectal cancer survivors in the EnCoRe study: A mixed-method study. *Eur J Oncol Nurs* 2020;45:101724. doi: 10.1016/j.ejon.2020.101724 [published Online First: 2020/02/12]
91. Koole JL, Bours MJL, Breedveld-Peters JJL, et al. Evaluating the Validity of a Food Frequency Questionnaire in Comparison with a 7-Day Dietary Record for Measuring Dietary Intake in a Population of Survivors of Colorectal Cancer. *J Acad Nutr Diet* 2020;120(2):245-57. doi: 10.1016/j.jand.2019.09.008 [published Online First: 2019/12/07]
92. Revesz D, van Kuijk SMJ, Mols F, et al. Development and internal validation of prediction models for colorectal cancer survivors to estimate the 1-year risk of low health-related quality of life in multiple domains. *BMC Med Inform Decis Mak* 2020;20(1):54. doi: 10.1186/s12911-020-1064-9 [published Online First: 2020/03/14]
93. Ose J, Botma A, Balavarca Y, et al. Pathway analysis of genetic variants in folate-mediated one-carbon metabolism-related genes and survival in a prospectively followed cohort of colorectal cancer patients. *Cancer Med* 2018 doi: 10.1002/cam4.1407 [published Online First: 2018/05/31]
94. Perry CA, Renna SA, Khitun E, et al. Ethnicity and race influence the folate status response to controlled folate intakes in young women. *The Journal of nutrition* 2004;134(7):1786-92. doi: 10.1093/jn/134.7.1786 [published Online First: 2004/07/01]
95. Nohr EA, Liew Z. How to investigate and adjust for selection bias in cohort studies. *Acta Obstet Gynecol Scand* 2018;97(4):407-16. doi: 10.1111/aogs.13319 [published Online First: 2018/02/08]

For peer review only

For peer review only - http://bmjopen.bmj.com/site/about/guidelines.xhtml

945 **Figure legends**

946 **Figure 1** FOCUS Consortium design

For peer review only

For peer review only - http://bmjopen.bmj.com/site/about/guidelines.xhtml

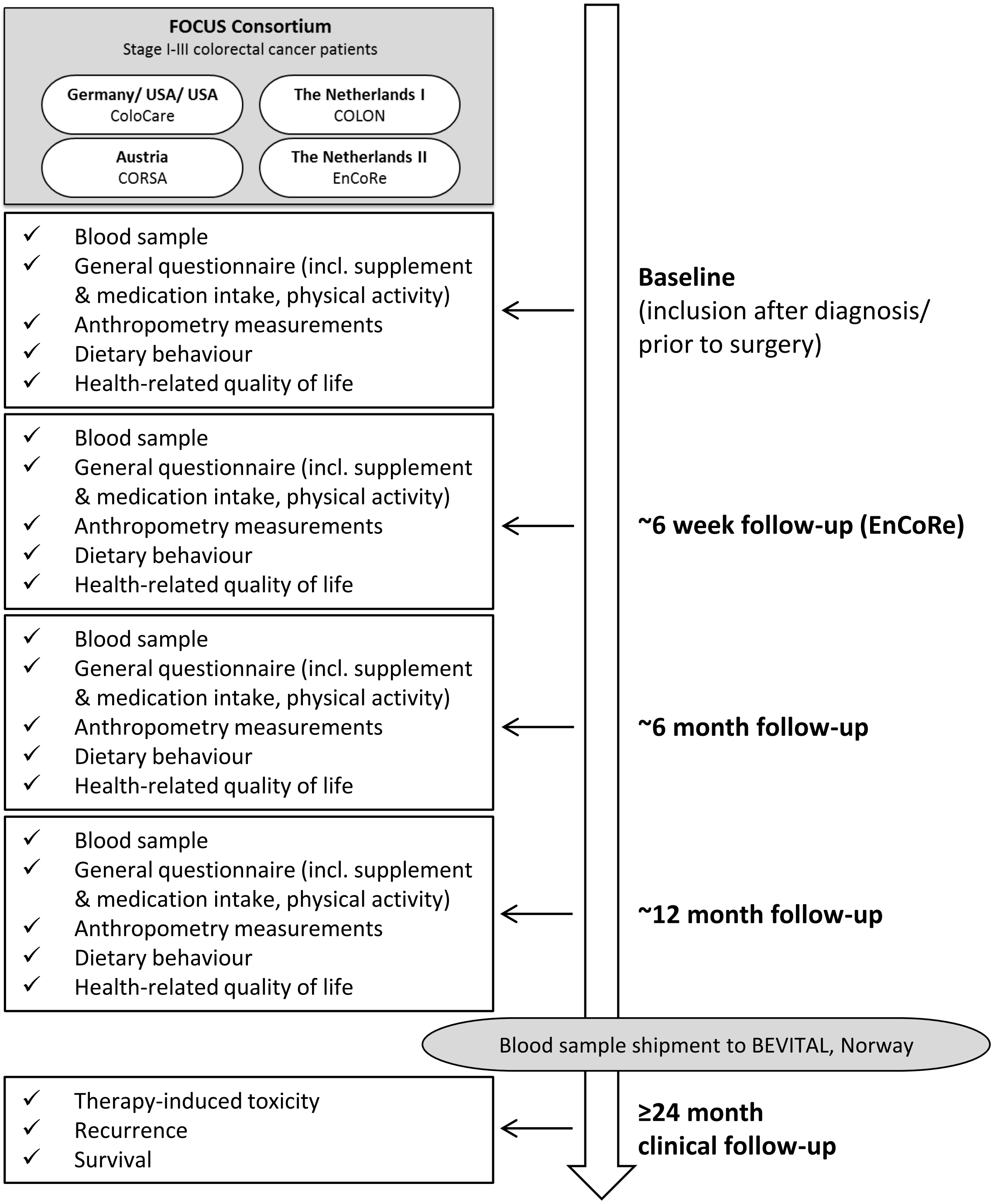
FOCUS Consortium
Stage I-III colorectal cancer patients
Germany/ USA/ USA
ColoCare
The Netherlands I
COLON
Austria
CORSA
The Netherlands II
EnCoRe
✓ Blood sample
✓ General questionnaire (incl. supplement & medication intake, physical activity)
✓ Anthropometry measurements
✓ Dietary behaviour
✓ Health-related quality of life
Baseline
(inclusion after diagnosis/ prior to surgery)
✓ Blood sample
✓ General questionnaire (incl. supplement & medication intake, physical activity)
✓ Anthropometry measurements
✓ Dietary behaviour
✓ Health-related quality of life
~6 week follow-up (EnCoRe)
✓ Blood sample
✓ General questionnaire (incl. supplement & medication intake, physical activity)
✓ Anthropometry measurements
✓ Dietary behaviour
✓ Health-related quality of life
~6 month follow-up
✓ Blood sample
✓ General questionnaire (incl. supplement & medication intake, physical activity)
✓ Anthropometry measurements
✓ Dietary behaviour
✓ Health-related quality of life
~12 month follow-up
Blood sample shipment to BEVITAL, Norway
✓ Therapy-induced toxicity
✓ Recurrence
✓ Survival
≥24 month
clinical follow-up

BMJ Open

Cohort profile: Biomarkers related to folate-dependent one-carbon metabolism in colorectal cancer recurrence and survival: The FOCUS Consortium

Journal:	*BMJ Open*
Manuscript ID	bmjopen-2022-062930.R2
Article Type:	Cohort profile
Date Submitted by the Author:	05-Oct-2022
Complete List of Authors:	Gigic, Biljana; University Hospital Heidelberg, Department of General, Visceral and Transplantation Surgery<br>van Roekel, Eline ; Maastricht University, Department of Epidemiology<br>Holowatyj, Andreana; Vanderbilt University Medical Center, Medicine; Vanderbilt-Ingram Cancer Center,<br>Brezina, Stefanie; Medical University of Vienna, Institute of Cancer Research, Department of Medicine I<br>Geijsen, Anne; Wageningen University & Research, Division of Human Nutrition and Health<br>Ulvik, Arve; BEVITAL<br>Ose, Jennifer; Huntsman Cancer Institute; University of Utah, Department of Population Health Sciences<br>Koole, Janna L.; Maastricht University, Department of Epidemiology<br>Damerell, Victoria; University Hospital Heidelberg, Department of General, Visceral and Transplantation Surgery<br>Kiblawi, Rama; Huntsman Cancer Institute; University of Utah, Department of Population Health Sciences<br>Gumpenberger, Tanja; Medical University of Vienna, Institute of Cancer Research, Department of Medicine I<br>Lin, Tengda; Huntsman Cancer Institute; University of Utah, Department of Population Health Sciences<br>Kvalheim, Gry; BEVITAL<br>Koelsch, Torsten; Heidelberg University Hospital, Department of General, Visceral and Transplantation Surgery<br>Kok, Dieuwertje; Wageningen University & Research, Division of Human Nutrition and Health<br>van Duijnhoven, Franzel J; Wageningen University & Research, Division of Human Nutrition and Health<br>Bours, Martijn J.; Maastricht University, Department of Epidemiology<br>Baierl, Andreas; University of Vienna, Department of Statistics and Operations Research<br>Li, Christopher I.; Fred Hutchinson Cancer Research Center<br>Grady, William; Fred Hutchinson Cancer Research Center; University of Washington School of Medicine, Department of Medicine<br>Vickers, Kathy; Fred Hutchinson Cancer Research Center<br>Habermann, Nina; European Molecular Biology Laboratory (EMBL), Genome Biology<br>Schneider, Martin; Heidelberg University Hospital, Department of

	General, Visceral and Transplantation Surgery<br>Kampman, Ellen; Wageningen University & Research, Division of Human Nutrition and Health<br>Ueland, Per Magne; BEVITAL<br>Ulrich, Alexis; Städtische Kliniken Neuss, Lukaskrankenhaus GmbH, Surgical Department I; University Hospital Heidelberg, Department of General, Visceral and Transplantation Surgery<br>Weijenberg, Matty ; Maastricht University, Department of Epidemiology<br>Gsur, Andrea; Medical University of Vienna, Institute of Cancer Research, Department of Medicine I<br>Ulrich, Cornelia; Huntsman Cancer Institute, Population Sciences; University of Utah, Department of Population Health Sciences
**Primary Subject Heading**:	Epidemiology
Secondary Subject Heading:	Nutrition and metabolism, Oncology, Public health
Keywords:	Epidemiology < ONCOLOGY, Nutritional support < ONCOLOGY, Gastrointestinal tumours < ONCOLOGY

SCHOLARONE™
Manuscripts

I, the Submitting Author has the right to grant and does grant on behalf of all authors of the Work (as defined in the below author licence), an exclusive licence and/or a non-exclusive licence for contributions from authors who are: i) UK Crown employees; ii) where BMJ has agreed a CC-BY licence shall apply, and/or iii) in accordance with the terms applicable for US Federal Government officers or employees acting as part of their official duties; on a worldwide, perpetual, irrevocable, royalty-free basis to BMJ Publishing Group Ltd ("BMJ") its licensees and where the relevant Journal is co-owned by BMJ to the co-owners of the Journal, to publish the Work in this journal and any other BMJ products and to exploit all rights, as set out in our licence.

The Submitting Author accepts and understands that any supply made under these terms is made by BMJ to the Submitting Author unless you are acting as an employee on behalf of your employer or a postgraduate student of an affiliated institution which is paying any applicable article publishing charge ("APC") for Open Access articles. Where the Submitting Author wishes to make the Work available on an Open Access basis (and intends to pay the relevant APC), the terms of reuse of such Open Access shall be governed by a Creative Commons licence – details of these licences and which Creative Commons licence will apply to this Work are set out in our licence referred to above.

Other than as permitted in any relevant BMJ Author's Self Archiving Policies, I confirm this Work has not been accepted for publication elsewhere, is not being considered for publication elsewhere and does not duplicate material already published. I confirm all authors consent to publication of this Work and authorise the granting of this licence.

For peer review only

Cohort profile: Biomarkers related to folate-dependent one-carbon metabolism in colorectal cancer recurrence and survival: The FOCUS Consortium

Authors:
Biljana Gigic[1], Eline H. van Roekel[2], Andreana N. Holowatyj[3,4,5,6], Stefanie Brezina[7], Anne J.M.R. Geijsen[8], Arve Ulvik[9], Jennifer Ose[3,4], Janna L. Koole[2], Victoria Damerell[1], Rama Kiblawi[3,4], Tanja Gumpenberger[7], Tengda Lin[3,4], Gry Kvalheim[9], Torsten Koelsch[1], Dieuwertje E. Kok[8], Fränzel J.B. van Duijnhoven[8], Martijn J. L. Bours[2], Andreas Baierl[10], Christopher I. Li[11], William M. Grady[11,12,13], Kathy Vickers[11], Nina Habermann[14], Martin Schneider[1], Ellen Kampman[8,16], Per-Magne Ueland[9,16], Alexis B. Ulrich[1,15,16], Matty Weijenberg[2,16], Andrea Gsur[7,16,17] and Cornelia M. Ulrich[3,4,16,17]

Affiliations:
[1] Department of General, Visceral and Transplantation Surgery, Heidelberg University Hospital, Heidelberg, Germany
[2] Department of Epidemiology, Maastricht University, Maastricht, the Netherlands
[3] Huntsman Cancer Institute, Salt Lake City, UT
[4] Department of Population Health Sciences, University of Utah, Salt Lake City, UT
[5] Department of Medicine, Vanderbilt University Medical Center, Nashville, TN
[6] Vanderbilt-Ingram Cancer Center, Nashville, TN
[7] Institute of Cancer Research, Department of Medicine I, Medical University of Vienna, Austria
[8] Division of Human Nutrition and Health, Wageningen University & Research, Wageningen, The Netherlands
[9] BEVITAL, Bergen, Norway
[10] Department of Statistics and Operations Research, University of Vienna, Austria
[11] Public Health Sciences Division, Fred Hutchinson Cancer Research Center, Seattle, Washington
[12] Department of Medicine, University of Washington School of Medicine, Seattle, Washington
[13] Clinical Research Division, Fred Hutchinson Cancer Research Center, Seattle, Washington
[14] Genome Biology, European Molecular Biology Laboratory (EMBL), Heidelberg, Germany
[15] Department of Surgery I, Lukas Hospital Neuss, Neuss, Germany
[16] These authors contributed equally
[17] Corresponding authors

Keywords: cohort, consortium, B-vitamins, folate, folic acid, one-carbon metabolism, dietary supplements, biomarkers, treatment toxicity, colorectal cancer, recurrence, survival

Corresponding author:
Cornelia M. Ulrich, MS, PhD
Chief Scientific Officer and Executive Director of the Cancer Center
Huntsman Cancer Institute
2000 Circle of Hope Drive
Salt Lake City, UT 84112
Phone: +1 (801) 213-5716
Email: neli.ulrich@hci.utah.edu

For peer review only - http://bmjopen.bmj.com/site/about/guidelines.xhtml

Abstract

Purpose: The overarching goal of the FOCUS (Biomarkers related to folate-dependent one-carbon metabolism in colorectal cancer recurrence and survival) Consortium is to unravel the effect of folate and folate-mediated one-carbon metabolism (FOCM) biomarkers on colorectal cancer (CRC) prognosis to provide clinically relevant advice on folate intake to cancer patients and define future tertiary prevention strategies.

Participants: The FOCUS Consortium is an international, prospective cohort of 2,401 women and men above 18 years of age who were diagnosed with a primary invasive non-metastatic (stage I-III) CRC. The Consortium comprises patients from Austria, two sites from the Netherlands, Germany and two sites from the USA. Patients are recruited after CRC diagnosis and followed at six and twelve months after enrolment. At each time point, sociodemographic data, data on health behavior, and clinical data are collected, blood samples are drawn.

Findings to date: An increased risk of cancer recurrences was observed among patients with higher compared to lower circulating folic acid concentrations. Furthermore, specific folate species within the FOCM pathway were associated with both inflammation and angiogenesis pathways among CRC patients. In addition, higher vitamin B_6 status was associated with better quality of life at six months post treatment.

Future plans: Better insights into the research on associations between folate and FOCM biomarkers and clinical outcomes in CRC patients will facilitate the development of guidelines regarding folate intake in order to provide clinically relevant advice to cancer patients, health professionals involved in patient care, and ultimately further tertiary prevention strategies in the future. The FOCUS Consortium offers an excellent infrastructure for short- and long-term research projects and for combining additional biomarkers and data resulting from the individual cohorts within the next years, e.g. microbiome data, omics- and multi-omics data or CT-quantified body composition data.

Strengths and limitations of this study

- FOCUS is the largest consortium to date addressing the research question of folate and FOCM biomarkers in relation to survival, recurrence, treatment toxicity, and health-related quality of life outcomes in CRC patients
- The cohorts included in the FOCUS Consortium are designed to enable future pooling of data using harmonized and standardized methods to collect data and biospecimens
- The pooled sample size provides sufficient power to investigate subgroup analyses across CRC patients
- Study time point definitions differ between some of the cohorts and have to be adapted for specific projects
- A selection bias for follow-up can arise because it is possible that patients who experience more severe toxicities, worse clinical outcome or health-related quality of life are underrepresented among those completing follow-ups

For peer review only - http://bmjopen.bmj.com/site/about/guidelines.xhtml

Introduction

Among men and women world-wide, colorectal cancer (CRC) accounts for nearly 10% of all incident cancer cases.[1] In Europe, 5-year survival for CRC patients is approximately 55%, with substantial differences by stage.[2 3] The number of CRC patients continues to increase due to implementation of improved screening strategies and/or enhanced treatment modalities.[4] Many cancer patients seek information on what they can do themselves to improve survival - for instance by improving their dietary habits and other lifestyle factors.[5] However, there may be behavioural aspects among individuals diagnosed with CRC, which may be harmful in some cases, such as the use of high-dose nutritional supplements containing synthetic folate. In general, knowledge on short and long-term effects is insufficient to make sound recommendations on use of dietary supplements, in particular folate, to cancer survivors, even though dietary supplements are used by 20%-85% of patients with cancer[6-8], highlighting the importance of thorough evaluation of potential benefits and harms and to support development of evidence-based recommendations on use of dietary supplements to cancer patients.

Folic acid is the synthetic form of the B-vitamin folate and is often used in dietary supplements or fortified foods. However, there is broad agreement that food folate is less bioavailable than folic acid with a median relative bioavailability of 65% (range: 44–80%), an estimate that approximates the 60% value derived from the Dietary Folate Equivalents equation.[9] Folate and folic acid play an important role in one-carbon metabolism, which is a complex series of biochemical reactions essential in nucleotide synthesis, methylation reactions and amino acid homeostasis.[10] One-carbon metabolism refers to a complex network of biochemical reactions linked to nucleotide synthesis and provides methyl groups for DNA, RNA or protein methylation. Thus, one-carbon metabolism is directly controlling processes determining DNA synthesis and integrity, both processes known to be linked to tumor growth.[11] To what extent folic acid supplement use and biomarkers of folate-mediated one-carbon metabolism (FOCM) impact cancer survival and treatment efficacy and toxicity still needs to be clarified.[11 12] Folate and FOCM biomarker deficiencies may increase cancer risk, but high levels, especially of synthetic folic acid, may also be driving factors in carcinogenesis.[6 13] An increasing body of evidence suggests that folate plays a dual role in carcinogenesis, involving both the prevention of early lesions and potential harm once pre-neoplastic or neoplastic lesions have developed.[6 12 14-20]

For peer review only - http://bmjopen.bmj.com/site/about/guidelines.xhtml

The overarching goal of the FOCUS (biomarkers related to FOlate-dependent one-carbon metabolism in Colorectal cancer recUrrence and Survival) Consortium is to study associations between folate and FOCM biomarkers and recurrence and survival in CRC patients. Better insights into these associations will facilitate the development of guidelines regarding folate intake in order to provide clinically relevant advice to cancer patients, health professionals involved in patient care, and ultimately further tertiary prevention strategies in the future. The FOCUS Consortium is a large-scale international consortium with CRC patients from six prospective cohort studies. The primary objectives of the FOCUS Consortium are: (i) to determine possible associations of folate and other FOCM biomarkers at diagnosis with recurrence or survival in non-metastatic (stage I-III) CRC; (ii) to elucidate whether biomarkers related to FOCM are associated with dietary and supplemental intake of these respective nutrients; (iii) to explore whether FOCM biomarkers are associated with treatment toxicity in CRC patients undergoing chemotherapy; and (iv) to collect comprehensive data of patient characteristics at baseline and follow-up including biomarkers of FOCM to establish an unique resource for future scientific research. The FOCUS Consortium is funded by the European Research Area Network (ERA-NET) on Translational Cancer Research (TRANSCAN).

The main purpose of the FOCUS Cohort profile is to (1) inform the scientific community about the FOCUS Consortium, (2) describe the complex methodology of a large consortium, (3) present ongoing studies using this infrastructure as well as (4) advise interested researchers of opportunities for collaboration. This joint research may lead to a better understanding of the role of folate- and FOCM-related mechanisms in the prognosis of CRC and be a precursor for data for future randomized controlled trials (RCTs), which will be critical for the development of guidelines regarding folate intake among CRC patients.

Cohort description

The FOCUS Consortium is an international, prospective consortium including six cohort studies recruiting women and men age 18 years and older diagnosed with a primary CRC. The FOCUS Consortium is comprised of patients from the ColoCare Study at the University of Heidelberg, Germany (n=298, 12.3%), the ColoCare Study at the Huntsman Cancer Institute in Salt Lake City, Utah, USA (n=80, 3.3%), the ColoCare Study at the Fred Hutchinson Cancer Research Center in Seattle, Washington, USA (n=157, 6.3%), the COLON Study (COlorectal cancer: Longitudinal, Observational study on Nutritional and lifestyle factors that may influence colorectal tumor recurrence, survival and quality of life) at Wageningen University & Research in the Netherlands (n=1,365, 56.1%), the CORSA Study (Colorectal

For peer review only - http://bmjopen.bmj.com/site/about/guidelines.xhtml

Cancer Study of Austria) at the Medical University of Vienna in Austria (n=218, 9.0%), and the EnCoRe Study (Energy for life after ColoRectal cancer) at Maastricht University Medical Center+ in the Netherlands (n=317, 13.0%).

In total, n=2,435 CRC patients were considered for the FOCUS Consortium, of which n=34 patients were excluded due to tumor staging being either 0 or IV leading to a total number of n=2,401 stage I-III CRC patients included in further analyses.

Patients were recruited after CRC diagnosis and repeated study measurements were conducted at time of recruitment, and at six and twelve months thereafter. At each study time point, sociodemographic data, data on lifestyle factors (e.g., diet, supplement use, physical activity), and clinical data (e.g., tumor site and stage, cancer treatment, treatment-induced toxicities, recurrence, survival) were collected, and blood samples were drawn (**Figure 1**). All individuals signed informed consent and the Institutional Review Board at each site approved the corresponding study. Below, a more specific description of each included study is provided.

The ColoCare Study

The ColoCare Study (ClinicalTrials.gov identifier: NCT02328677) is an ongoing international, multi-center prospective cohort study among women and men newly diagnosed with a primary invasive CRC, with the goal to investigate predictors of cancer recurrence, survival, treatment toxicities and health-related quality of life.[4 21] Three ColoCare Consortium sites participate in the FOCUS Consortium: the Fred Hutchinson Cancer Research Center in Seattle (Washington, USA), and the University Hospital Heidelberg, Heidelberg (Germany) as well as the Huntsman Cancer Institute in Salt Lake City (Utah, USA). Patients are enrolled prior to undergoing CRC surgery according to the following inclusion criteria: individuals who are 18 years of age and older, newly diagnosed (i.e., non-recurrent) with invasive CRC. Blood draws and other biospecimens are obtained prior to surgery and at regular intervals (e.g., six, twelve, twenty-four months). Questionnaires are administered to assess lifestyle behavior, health-related quality of life, and clinical outcomes such as CRC recurrence, treatment, and treatment symptoms at each study time point. Clinical data are obtained through reviews of patient medical records, pathology and imaging reports. Vital status is obtained through medical records, routine follow-up mailings, periodic requests for outside medical records, and state or national cancer and death registries. The Heidelberg ColoCare Study was approved by the Ethics Committee of the Medical Faculty of Heidelberg (approval no. S-310/2001 and S-134/2016). The ColoCare Study at the Huntsman Cancer Institute and

For peer review only - http://bmjopen.bmj.com/site/about/guidelines.xhtml

the Fred Hutchinson Cancer Research Center were approved by the respective IRBs (#77147 and #6407).

The COLON Study

The COLON Study (ClinicalTrials.gov identifier: NCT03191110) started in 2010 and is an ongoing, multicenter prospective cohort study specifically designed to assess associations between nutrition, lifestyle, and dietary supplement use with quality of life, CRC recurrence and survival among CRC patients (stages I-IV).[22] Persons with a history of CRC or (partial) bowel resection, chronic inflammatory bowel disease, or a known hereditary CRC syndrome are excluded from the study. Patients are recruited from eleven regional and academic hospitals prior to surgery. Individuals donate blood samples and provide information on diet, lifestyle, and dietary supplement use at CRC diagnosis, i.e. baseline, and at six, twelve (chemotherapy patients only), twenty-four and sixty months after diagnosis. Clinical data are collected through review of medical records (treatment-induced toxicity) or through linkage with the Dutch ColoRectal Audit (DCRA). Mortality data (i.e. death or alive and date of death) are retrieved from the Municipal Personal Records Database. Recurrence data have been retrieved in collaboration with the Netherlands Cancer Registry. Ethical approval for the study was granted by the Committee on Research involving Human Subjects, region Arnhem-Nijmegen under file number 2009/349.

The CORSA Study

CORSA is an ongoing case-control study of women and men recruiting CRC patients, patients with high and low risk adenomas and population-based colonoscopy negative controls, with an age range between 30 and 90 years. Since 2003, more than 13,500 participants have been recruited across nine sites in Austria. The multicentre recruitment within CORSA follows standardized protocols resulting in consistent data from all recruitment sites. These sites include the Medical University of Vienna (Department of Surgery), two hospitals in Vienna (Clinic Favoriten and Clinic Landstraße), and the Hospital Wiener Neustadt in Lower Austria. Furthermore, the recruitment for CORSA was performed in four hospitals in the federal state Burgenland within the population-based screening program "Burgenland PREvention Trial of colorectal cancer DIsease with ImmunologiCal Testing" (B-PREDICT). B-PREDICT is a two-stage screening project where more than 150,000 inhabitants of Burgenland aged between 40 and 80 are invited annually to participate in this program using a Fecal Immunochemical Test (FIT) as an initial screening. FIT-positive

For peer review only - http://bmjopen.bmj.com/site/about/guidelines.xhtml

(≥10 µg hemoglobin / g feces) tested individuals are offered a complete colonoscopy and are asked to participate in CORSA. Biospecimen are collected at each site using harmonized protocols. CORSA participants provide a basic CORSA questionnaire assessing data on body mass index (BMI), smoking history, alcohol consumption, education level, family status, profession, basic dietary habits, and diabetes. Clinical data are abstracted from medical records and processed in a structured database following standardized documentation guidelines and according to the General Data Protection Regulation (GDPR). All subjects gave written informed consent and the study was approved by the institutional review boards: 'Ethikkommission Burgenland' (33/2010), by the ethical review committee of the Medical University of Vienna (1160/2016) and by the "Ethikkommission der Stadt Wien" (EK 06-150VK).

The EnCoRe Study

The EnCoRe Study is a prospective cohort study initiated in 2012 at the Maastricht University Medical Center+ in the Netherlands that focuses on the importance of lifestyle factors for quality of life, recurrence and survival of CRC patients.[23] The EnCoRe Study is registered in the Netherlands Trial Registry for experimental and observational studies (www.trialregister.nl, registration number 7099). Stage I-III CRC patients are enrolled at diagnosis at three hospitals in the South-Eastern region of the Netherlands and followed up until five years after completion of treatment with repeated measurements at diagnosis (pre-treatment), and at six weeks and six, twelve, twenty-four, and sixty months after the end of the initial anti-cancer treatment (i.e., surgery, chemotherapy, radiotherapy). Patients with stage IV CRC, an inability to understand the Dutch language in speech or writing, with comorbidities obstructing successful participation (e.g., Alzheimer disease), or with severe visibility or hearing disorders are excluded from the study. Repeated home visits by trained dieticians are conducted and data are collected amongst others on sociodemographic factors, quality of life, functioning, physical activity, comorbidity, dietary intake, supplement use, and anthropometry. In addition, clinical data are collected from hospital records and blood samples are drawn at all time points. Recurrence data have been retrieved in collaboration with the Netherlands Cancer Registry. Mortality data (i.e. death or alive and date of death) are retrieved from the Municipal Personal Records Database. The EnCoRe Study was approved by the Medical Ethics Committee of the Maastricht University Hospital and Maastricht University (METC 11-3-075).

For peer review only

For peer review only - http://bmjopen.bmj.com/site/about/guidelines.xhtml

The FOCUS Consortium

Sociodemographic and clinical characteristics of the 2,401 participants in the FOCUS Consortium are presented in **Table 1**. The mean age at CRC diagnosis was 65.4 years (standard deviation (SD): 10.2; range: 22 to 93 years), and the majority of participants were male (64.0%). The mean BMI was 27.0 kg/m² (SD: 4.6).

In terms of education status, 35.9% of the patients reported lower education, 28.8% reported intermediate education and 27.3% high education. Most of the patients were married or part of a living community (73.1%). About half of the overall cohort reported to be a former smoker (50.8%), 31.7% were never smokers, and 12.1% were current smokers (5.4% were unknown). Median alcohol intake was 7.9 g/day. In total, 16.9% of the patients reported regular dietary supplementation (i.e., at least once per week during the last four weeks) of folic acid. Over half of all participants (54.6%) reported adherence to the physical activity guidelines of at least 150 minutes per week of moderate-to-vigorous physical activity. Lifestyle characteristics of the FOCUS cohort are presented in **Table 2**.

Regarding clinical characteristics, 57.0% of participants were diagnosed with colon cancer, 33.9% with rectal cancer, and 1.1% with rectosigmoid cancer (8.1% were of unknown tumor subsite). In total, 26.0% were diagnosed with stage I, 28.7% with stage II, and 40.2% with stage III CRC. Approximately 1% of participants were classified with an unspecified cancer stage, as distinction between stage I and II or II and III was not possible, and for 4.4% of the total population the cancer stage was unknown. In total, 89.3% of patients underwent surgery, whereas neoadjuvant chemotherapy was administered to 10.6% of patients and 26.8% received adjuvant chemotherapy. Neoadjuvant radiotherapy was administered to 20.7% of patients and adjuvant radiotherapy in 9.4% of the population.

Patient and public involvement

Patients and the public were not involved in the design, conduct, reporting or dissemination plans of this research.

For peer review only - http://bmjopen.bmj.com/site/about/guidelines.xhtml

Table 1: Sociodemographic and clinical factors of eligible FOCUS Consortium participants at baseline (n=2,401)

	Overall	ColoCare HD	ColoCare FHCRC	ColoCare HCI	COLON	CORSA	EnCoRe
n (%)	2,401 (100)	298 (12.4)	150 (6.3)	80 (3.3)	1,338 (55.7)	218 (9.1)	317 (13.2)
**Age**							
**Mean** y ± SD	65.4 ± 10.2	63.7 ± 12.2	58.3 ± 12.8	61.3 ± 11.2	66.1 ± 8.7	67.3 ± 12.0	66.9 ± 9.3
**Sex**							
**Female** n (%)	864 (36.0)	100 (33.6)	67 (44.7)	32 (40.0)	486 (36.3)	76 (34.9)	103 (32.5)
**Male** n (%)	1,537 (64.0)	198 (66.4)	83 (55.3)	48 (60.0)	852 (63.7)	142 (65.1)	214 (67.5)
**Education**							
**Low** n (%)	861 (35.9)	123 (41.3)	18 (12.0)	17 (21.3)	543 (40.6)	73 (33.5)	87 (27.4)
**Intermediate** n (%)	694 (28.9)	57 (19.1)	71 (47.3)	40 (50.0)	332 (24.8)	73 (33.5)	121 (38.2)
**High** n (%)	658 (27.4)	88 (29.5)	28 (18.7)	8 (10.0)	417 (31.7)	14 (6.4)	103 (32.5)
**Unknown** n (%)	188 (7.8)	30 (10.1)	33 (22.0)	15 (18.7)	46 (3.4)	58 (26.6)	6 (1.9)
**Marital status**							
**Unmarried** n (%)	122 (5.1)	19 (6.4)	15 (10.0)	2 (2.5)	60 (4.5)	10 (4.6)	16 (5.1)
**Married** n (%)	1,738 (72.4)	193 (64.8)	71 (47.3)	50 (62.5)	1,071 (80.0)	113 (51.8)	240 (75.7)
**Divorced/Separated** n (%)	138 (5.7)	24 (8.0)	11 (7.3)	10 (12.5)	64 (4.8)	14 (6.4)	15 (4.7)
**Widowed** n (%)	194 (8.1)	31 (10.4)	7 (4.7)	1 (1.3)	90 (6.7)	26 (11.9)	39 (12.3)
**Living community** n (%)	20 (0.8)	0	13 (8.7)	2 (2.5)	4 (0.3)	1 (0.5)	0
**Unknown** n (%)	189 (8.9)	31 (10.4)	33 (22.0)	15 (18.7)	49 (3.7)	54 (24.8)	7 (2.2)
**Smoking status**							
**Current** n (%)	290 (12.1)	53 (17.8)	8 (5.3)	6 (7.5)	143 (10.7)	38 (17.4)	42 (13.3)
**Former** n (%)	1,219 (50.8)	132 (44.3)	53 (35.32)	21 (26.3)	767 (57.3)	76 (34.9)	170 (53.6)
**Never** n (%)	767 (31.9)	90 (30.2)	56 (37.3)	39 (48.7)	386 (28.9)	97 (44.5)	99 (31.2)
**Unknown** n (%)	125 (5.2)	23 (7.7)	33 (22.0)	14 (17.5)	42 (3.1)	7 (3.2)	6 (1.9)
**Alcohol intake**							
**Mean** g/day ± SD	13.6 ± 17.1	16.1 ± 19.2	8.8 ± 18.5	2.3 ± 10.2	14.0 ± 17.0		12.9 ± 16.5
**Unknown** n (%)	633 (26.4)	208 (69.8)	81 (54.0)	60 (75.0)	54 (4.1)	218 (100)	12 (3.8)
**BMI at diagnosis**							
**Mean** kg/m² ± SD	27.1 ± 4.6	26.6 ± 4.1	28.7 ± 7.3	29.4 ± 7.5	26.6 ± 4.0	27.7 ± 4.4	28.3 ± 4.6
**Unknown** n (%)	42 (1.7)	2 (0.7)	0	14 (17.5)	10 (0.8)	14 (6.4)	2 (0.6)

For peer review only - http://bmjopen.bmj.com/site/about/guidelines.xhtml

	Overall	ColoCare HD	ColoCare FHCRC	ColoCare HCI	COLON	CORSA	EnCoRe
**Tumor site**							
**Colon** n (%)	1,450 (60.4)	140 (57.0)	75 (50.0)	45 (56.3)	867 (64.8)	131 (60.1)	192 (60.6)
**Rectum** n (%)	862 (35.9)	143 (48.0)	60 (40.0)	22 (27.5)	434 (32.4)	78 (35.8)	125 (39.4)
**Rectosigmoid** n (%)	42 (1.7)	15 (5.0)	15 (10.0)	7 (8.7)	0	5 (2.3)	0
**Unknown** n (%)	47 (2.0)	0	0	6 (7.5)	37 (2.8)	4 (1.8)	0
**Tumor stage**							
**I** n (%)	629 (26.2)	76 (25.5)	35 (23.3)	21 (26.3)	326 (24.4)	82 (37.6)	89 (28.1)
**II** n (%)	694 (28.9)	114 (38.3)	49 (32.7)	23 (28.7)	386 (28.9)	52 (23.9)	70 (22.1)
**III** n (%)	973 (40.5)	108 (36.2)	66 (44.0)	36 (45.0)	564 (42.1)	52 (23.9)	147 (46.4)
**Unspecified*** n (%)	16 (0.7)	0	0	0	4 (0.3)	12 (5.5)	0
**Unknown** n (%)	89 (3.7)	0	0	0	58 (4.3)	20 (9.2)	11 (3.5)
**Treatment**							
**Neoadjuvant chemotherapy** n (%)	331 (13.8)	55 (18.5)	47 (31.3)	15 (18.8)	138 (10.3)	14 (6.4)	62 (19.6)
**Unknown** n (%)	58 (2.4)	2 (0.7)	1 (0.7)	10 (12.5)	38 (2.8)	7 (3.2)	0
**Neoadjuvant radiotherapy** n (%)	523 (21.8)	72 (24.2)	48 (32.0)	13 (16.3)	290 (21.7)	14 (6.4)	86 (27.1)
**Unknown** n (%)	58 (2.4)	2 (0.7)	1 (0.7)	10 (12.5)	38 (2.8)	7 (3.2)	0
**Adjuvant chemotherapy** n (%)	682 (28.4)	100 (33.6)	80 (53.3)	32 (40.0)	315 (23.8)	56 (25.7)	99 (31.2)
**Unknown** n (%)	121 (5.0)	17 (5.7)	3 (2.0)	28 (35.0)	63 (4.7)	9 (4.1)	1 (0.3)
**Adjuvant radiotherapy** n (%)	227 (9.5)	188 (63.1)	7 (4.7)	1 (1.3)	16 (1.2)	13 (6.0)	2 (0.6)
**Unknown** n (%)	164 (6.8)	17 (5.7)	3 (2.0)	28 (35.0)	106 (7.9)	9 (4.1)	1 (0.3)
**Surgery** n (%)	2,291 (95.4)	298 (100.0)	149 (99.3)	71 (88.7)	1,290 (96.4)	195 (89.5)	288 (90.9)
**Unknown** n (%)	44 (1.8)	0	0	3 (3.8)	39 (2.9)	1 (0.5)	1 (0.3)

*Distinguishing between stage I and II or II and III not possible

For peer review only

For peer review only - http://bmjopen.bmj.com/site/about/guidelines.xhtml

Table 2: Lifestyle characteristics of eligible FOCUS Consortium participants at baseline (n=2,401)

	Overall	ColoCare HD	ColoCare FHCRC	ColoCare HCI	COLON	CORSA	EnCoRe
n (%)	2,401 (100)	298 (12.4)	150 (6.3)	80 (3.3)	1,338 (55.7)	218 (9.1)	317 (13.2)
**Folic acid supplementation**							
**Yes** n (%)	405 (16.9)	4 (1.3)	9 (5.9)	2 (2.5)	325 (24.3)		65 (20.5)
**Unknown** n (%)	383 (15.9)	27 (9.1)	86 (56.6)	14 (17.5)	38 (2.8)	218 (100)	0
**Vitamin B_{12} supplementation**							
**Yes** n (%)	430 (17.9)	10 (3.4)	8 (5.3)	14 (17.5)	336 (25.1)		62 (19.6)
**Unknown** n (%)	384 (16.0)	27 (9.1)	86 (56.6)	14 (17.5)	39 (2.9)	218 (100)	0
**Vitamin B_6 supplementation**							
**Yes** n (%)	77 (3.2)	8 (2.7)	4 (2.6)	4 (5.0)			61 (19.2)
**Unknown** n (%)	1,684 (70.1)	27 (9.1)	86 (56.6)	14 (17.5)	1,339 (100)	218 (100)	0
**Vitamin B_2 supplementation**							
**Yes** n (%)	62 (2.6)	0	2 (1.3)	0			60 (18.9)
**Unknown** n (%)	1,684 (70.1)	27 (9.1)	86 (56.6)	14 (17.5)	1,339 (100)	218 (100)	0
**Dietary intake of folate equivalents**							
**Mean** µg/day ± SD	227.1 ± 94.3	115.1 ± 38.1	149.6 ± 97.5	116.2 ± 112.8	229.0 ± 88.7		276.3 ± 82.2
**Unknown** n (%)	638 (26.5)	208 (69.8)	83 (54.6)	60 (75.0)	57 (4.3)	218 (100)	12 (3.8)
**Dietary intake of vitamin B_{12}**							
**Mean** µg/day ± SD	4.6 ± 2.4	6.2 ± 2.9	7.1 ± 5.8	4.2 ± 2.2	4.3 ± 2.1		4.6 ± 2.0
**Unknown** n (%)	638 (26.5)	208 (69.8)	83 (54.6)	60 (75.0)	57 (4.3)	218 (100)	12 (3.8)
**Dietary intake of vitamin B_6**							
**Mean** mg/day ± SD	1.5 ± 0.5	1.7 ± 0.5	2.0 ± 0.7	1.5 ± 0.7	1.5 ± 0.5		1.8 ± 0.5
**Unknown** n (%)	638 (26.5)	208 (69.8)	83 (54.6)	60 (75.0)	57 (4.3)	218 (100)	12 (3.8)
**Dietary intake of vitamin B_2**							
**Mean** mg/day ± SD	1.4 ± 0.5	1.5 ± 0.5	2.2 ± 1.0	1.6 ± 0.8	1.3 ± 0.4		1.4 ± 0.5
**Unknown** n (%)	638 (26.5)	208 (69.8)	83 (54.6)	60 (75.0)	57 (4.3)	218 (100)	12 (3.8)
**Total energy intake**							
**Mean** kcal/day ± SD	1,941.8 ± 593.6	2,330.9 ± 714.0	1835.0 ± 775.7	1,398.5 ± 597.8	1,856.9 ± 517.2		2,243.7 ± 651.0
**Unknown** n (%)	638 (26.5)	208 (69.8)	83 (54.6)	60 (75.0)	57 (4.3)	218 (100)	12 (3.8)
**Adherence to physical activity guidelines***							

For peer review only - http://bmjopen.bmj.com/site/about/guidelines.xhtml

**Yes** n (%)	1,310 (54.5)	84 (28.2)	35 (23.0)	20 (25.0)	941 (70.3)		230 (72.6)
**Unknown** n (%)	370 (15.4)	33 (11.1)	53 (34.9)	15 (18.7)	45 (3.4)	218 (100)	6 (1.9)

*Self-reported engagement in at least 150 minutes per week of moderate-to-vigorous physical activity

For peer review only

Cohort follow-up

At each study site, collection of biospecimen, clinical, demographic, questionnaire and anthropometric data occurred at baseline, and at six and twelve months following recruitment. Baseline measurements in CORSA, COLON and EnCoRe have been performed at diagnosis (preferably prior to any cancer treatment) while in the ColoCare Study such measurements have been done prior to surgery (i.e. neoadjuvant treatment might have been applied prior to baseline blood and data collection).

Blood samples at baseline were collected from n=2,132 (88.8%) study participants. N=1,537 (64.0%) participants donated blood at the 6 month and n=614 (25.6%) at the 12 month follow-up time point. Additionally, n=251 (10.5%) EnCoRe patients provided blood samples at 6 weeks post-treatment, a subset specifically of interest at the Maastricht study site.

Data collection

Harmonized baseline and follow-up time points are summarized in **Table 3** and are briefly described below.

Lifestyle and demographic data

ColoCare questionnaires and standardized Food Frequency Questionnaires (FFQ) are used to assess intake of dietary supplements and medication, smoking, dietary intake and other health behaviors at each study time point. In COLON, patients provide information on diet, lifestyle, and supplement use via COLON questionnaires and FFQs. Patients from the CORSA Study are requested to complete a questionnaire assessing anthropometric and demographic factors. Patients enrolled in EnCoRe receive repeated home visits by trained dieticians, where extensive measurements are performed that include assessment of demographic data, physical activity (both questionnaire- and accelerometry-based) and smoking behavior (questionnaire data), dietary intake (FFQs at diagnosis and 7-day food diaries at follow-up measurements), supplement use (registered by dieticians), and anthropometry measurements.

For peer review only

Table 3: Variables available at baseline, and at 6 and 12 month follow-up within the FOCUS Consortium.

Category	Variables	Baseline	6m	12m
**Demographics**	Age	x	x	x
	Gender	x		
	Highest education	x		
	Social status	x		
	Height	x		
	Weight	x	x	x
	BMI	x	x	x
	Smoking status	x	x	x
	Smoking duration	x	x	x
	Smoking pack years	x	x	x
	Menopausal status	x		
	Postmenopausal hormone use	x		
	Race	x		
**Cancer characteristics**	Cancer site	x		
	Cancer stage	x		
	TNM classification	x		
**Treatment**	Preoperative chemotherapy	x		
	Preoperative radiotherapy	x		
	Surgery	x	x	
	Postoperative chemotherapy		x	x
	Postoperative radiotherapy		x	x
**Supplement intake**	Folic acid supplements	x	x	x
	Vitamin B_2 supplements	x	x	x
	Vitamin B_6 supplements	x	x	x
	Vitamin B_{12} supplements	x	x	x
	Vitamin A supplements	x	x	x
	Vitamin C supplements	x	x	x
	Vitamin D supplements	x	x	x
**Supplement intake**	Vitamin E supplements	x	x	x
	Calcium supplements	x	x	x
	Magnesium supplements	x	x	x
	Iron supplements	x	x	x
	Multivitamins	x	x	x
**Dietary nutrients**	Folate equivalents	x	x	x
	Vitamin B_2	x	x	x
	Vitamin B_6	x	x	x
	Vitamin B_{12}	x	x	x
	Vitamin A	x	x	x
	Vitamin C	x	x	x
	Vitamin D	x	x	x
	Vitamin E	x	x	x
	Total protein	x	x	x
	Total fat	x	x	x
	Total carbohydrate	x	x	x

For peer review only

**Dietary nutrients**	Fibre	x	x	x
	Saturated fatty acids	x	x	x
	Monounsaturated fatty acids	x	x	x
	Polyunsaturated fatty acids	x	x	x
	Alcohol	x	x	x
	Total energy	x	x	x
**Physical activity**	Light physical activity	x	x	x
	Moderate physical activity	x	x	x
	Vigorous physical activity	x	x	x
	Adherence to physical activity guidelines	x	x	x
**Medical history**	Diabetes mellitus	x	x	x
	Asthma, Chronical Bronchitis, COPD, Emphysema	x		
	Heart attack, Heart Failure	x		
	Hypertension	x		
	Stroke	x		
	Ulcer of Stomach or Duodenum	x		
	Hypothyroidism/Hyperthyroidism	x		
	Systemic Lupus Erythematosus	x		
	Inflammatory Bowel Disease (Crohn's disease, ulcerative colitis)	x		
	Familial Adenomatous Polyposis	x		
**Medical history**	Lynch Syndrome (hereditary nonpolyposis colorectal cancer)	x		
**Regular medication use**	Aspirin	x	x	x
	NSAID	x	x	x
	Ibuprofen	x	x	x
	Naproxen	x	x	x
	Celecoxib/Etoricoxib	x	x	x
**Health-related quality of life**	EORTC QLQ-C30	x	x	x
	EORTC QLQ-CR29	x	x	x
	EORTC QLQ-CIPN20	x	x	x
**General information**	Date of questionnaire completion	x	x	x
	Date of blood collection	x	x	x
	Freeze-thaw cycles of blood samples	x	x	x
	Hemolysis	x	x	x
	Time between blood draw and processing/storage	x	x	x

NSAID=non-steroidal anti-inflammatory drug; EORTC=European Organization for Research and Treatment of Cancer; QLQ=quality of life questionnaire; C30=core 30-items; CR29=colon/rectum 29-items; CIPN20=chemotherapy-induced polyneuropathy 20-items.

306

For peer review only

Clinical data and outcomes - Medical chart abstraction

All cancers and medical diagnoses are classified according to ICD-10 (International Classification of Diseases, Tenth Revision) codes. Details on CRC treatment, including type of treatment regimens and treatment toxicity, are abstracted via medical record for all cohorts. Detailed information on the primary outcomes of interest, CRC recurrence and survival, are ascertained through reviews of medical records, pathology reports, and imaging reports documenting the diagnosis of a recurrence. Data on recurrence and vital status is supplemented from the clinical cancer registries and survival data is verified by the inhabitant registries that exist at all study sites. For COLON and EnCoRe, all data on recurrence are retrieved in collaboration with the Netherlands Cancer Registry. Moreover, survival data for CORSA participants was obtained by the Main Association of Austrian Social Insurance Carriers as well as from Statistics Austria.

Patient-reported outcomes

Health-related quality of life is assessed by the validated and widely used cancer-specific 30-item core questionnaire QLQ-C30 and the 29-item CRC module QLQ-CR29, developed by the European Organization for Research and Treatment of Cancer (EORTC).[24] Chemotherapy-induced peripheral neuropathy (CIPN), which is a common toxicity in CRC patients, is measured by the EORTC QLQ-CIPN20.[25] Patient-reported outcomes are available for ColoCare Heidelberg, COLON and EnCoRe.

Biomarkers of FOCM

Blood is processed in identical settings across all study centers. Plasma (CORSA, COLON and EnCoRe) and serum (for the three ColoCare Study sites) samples were collected and immediately centrifuged, aliquoted, and stored at -80°C.

All biological analyses were performed at BEVITAL AS (Bergen, Norway, http://www.bevital.no), which carried out metabolic profiling of biomarkers allocated to seven complementary analytical platforms. Apart from analyses of microbiological active folate[26] and vitamin B_{12}[27]), all analyses were based on mass spectrometry. Circulating folate, separate folate species, 5-methyltetrahydrofolate (the main form in plasma), folic acid (folate in supplements), 4a-OH 5-methyltetrahydrofolate (the primary oxidation form of circulating folate), folate catabolites (p-aminobenzoylglutamate and acetyl p-aminobenzoylglutamate)[28], B_6, B_1 and B_3 vitamins, kynurenines, cotinine, trans-3'-hydroxycotinine, trigonelline[29], choline and its metabolites, creatinine, methylhistidines, arginine and methylated arginines[30]

For peer review only

For peer review only - http://bmjopen.bmj.com/site/about/guidelines.xhtml

were analyzed by liquid chromatography-tandem mass spectroscopy, whereas other amino acids, in addition to total homocysteine, total cysteine and methylmalonic acid were analyzed by gass chromatography-tandem mass spectroscopy.[31] C-reactive protein (hsCRP) and cystatin C and its variants were measured by MALDI-TOF mass spectroscopy.[32]

A comprehensive overview of the panel of biomarkers measured in blood samples of patients enrolled in the FOCUS Consortium is provided in **Table 4**.

Findings to date

The FOCUS Consortium provides a unique opportunity to conduct comprehensive research on folate and FOCM biomarkers status in the tertiary prevention of CRC using data collected both at diagnosis and during standardized follow-up time points. This well-characterized study design provides sufficient statistical power to discern prospective associations with relevant clinical outcomes, including CRC recurrence and survival within relevant subgroups.

Key findings and publications

We investigated associations of circulating concentrations of folate, folic acid, and folate catabolites pABG and apABG, measured around time of diagnosis, with recurrence and survival among 2024 patients diagnosed with stage I-III CRC within the international FOCUS Consortium. We did not observe any statistically significant associations for folate, pABG, and apABG concentrations. However, an increased risk of cancer recurrences was observed among patients with higher compared to lower circulating folic acid concentrations.[33] Further, Kiblawi et al. measured associations between one-carbon metabolites, inflammation- and angiogenesis-related biomarkers in a cross-sectional analysis of 238 patients from the ColoCare Heidelberg cohort. The study showed that specific folate species within the one-carbon metabolism pathway are associated with both inflammation and angiogenesis pathways among CRC patients. In particular, vitamin B6 species, pyridoxal 5'-phosphate (PLP), pyridoxal (PL) and pyridoxic acid (PA), were inversely associated with inflammatory biomarkers C-reactive protein (CRP), serum amyloid A (SAA), IL-6 and IL-8. Thiamine and thiamine monophosphate were inversely correlated with the CRP and IL-6. In addition, positive correlations of PA, PL and PLP with angiogenesis biomarker VEGF-D were observed. Our findings reinforce the notion that B vitamins involved in the one-carbon metabolism may be correlated with carcinogenic processes.[34] This and further research will support the evidence base needed for the development of dietary guidelines for CRC patients.

For peer review only - http://bmjopen.bmj.com/site/about/guidelines.xhtml

Table 4: Measured metabolites and biomarkers within the FOCUS Consortium

Folate and one-carbon metabolites	Abbreviation	Description
**Anthranilic acid**	AA	Tryptophan metabolite
**Asymmetric dimethylarginine**	ADMA	Inhibitor of nitric oxide synthase
**α-Ketoglutaric acid**	aKG	Keto acid of the Krebs cycle
**Alanine**	Ala	Amino acid
**Acetamidobenzoylglutamate**	apABG	Folate catabolite
**Arginine**	Arg	Amino acid
**Asparagine**	Asn	Amino acid
**Aspartic acid**	Asp	Amino acid
**Betaine**	Betaine	Methyl donor
**Choline**	Choline	Methyl donor
**C-reactive protein**	hsCRP	Inflammatory marker
**Cystatin C-desS**	CnCds	Cystatin C isoform
**Cystatin C-desSSP**	CnDcssp	Cystatin C isoform
**Cystatin C**	CnCn	Marker of kidney function
**3Pro-OH cystatin C**	CnCo	Cystatin C isoform
**3Pro-OH cystatin C-desS**	CnCods	Cystatin C isoform
**Total concentration of detected cystatin C isoforms**	CnCt	Marker of kidney function
**Cobalamin**	B_{12}	Vitamin B_{12}
**Cotinine**	Cot	Nicotine metabolite
**Creatinine**	Creat	Marker of kidney function
**C-reactive protein**	CRP	Inflammation
**Cystathionine**	Cysta	Thioether, transsulfuration intermediate
**Dimethylglycine**	DMG	Betaine metabolite
**Folic acid**	B_9	Synthetic form of folate
**Flavin mononucleotide**	FMN	B_2 vitamin
**5-Formyl-tetrahydrofolate**	fTHF	Folate species
**Glutamine**	Gln	Amino acid
**Glutamic acid**	Glu	Amino acid
**Glycine**	Gly	Amino acid
**3-Hydroxyanthranilic acid**	HAA	Tryptophan metabolite
**Homoarginine**	hArg	Amino acid
**Histidine**	Hist	Tryptophan metabolite
**3-Hydroxykynurenine**	HK	Tryptophan metabolite
**4-Alfa-hydroxy-5-methyl-THF**	hmTHF	Folate oxidation product
**Isoleucine**	Ile	Amino acid
**Kynurenic acid**	KA	Tryptophan metabolite
**Kynurenine**	Kyn	Tryptophan metabolite
**Leucine**	Leu	Amino acid
**Lysine**	Lys	Amino acid
**3-Methylhistidine (3-MH)**	m3His	Marker of muscle degradation and meat intake
**1-Methylhistidine (1-MH)**	m1His	Marker of muscle degradation and meat intake
**Methionine**	Met	Amino acid
**Methionine sulfoxide**	MetSo	Oxidation product of Met
**Methylmalonic acid**	MMA	Marker of B_{12} status
**N1-methylnicotinamide**	mNAM	B_3 vitamin
**5-Methyl-tetrahydrofolate**	mTHF	Folate species

For peer review only - http://bmjopen.bmj.com/site/about/guidelines.xhtml

**Nicotinic acid**	NA	B_3 vitamin
**Nicotinamide**	NAM	B_3 vitamin
**Neopterin**	Neopt	Inflammatory marker
**Trans-3'-hydroxycotinine**	OHCot	Nicotine metabolite
**Ornithine**	Orn	Amino acid
**4-Pyridoxic acid**	PA	Vitamin B_6 catabolite
**Para-aminobenzoylglutamate**	pABG	Folate catabolite
**Phenylalanine**	Phe	Amino acid
**Picolinic acid**	Pic	Tryptophan metabolite
**Pyridoxal**	PL	B_6 vitamin
**Pyridoxal 5'-phosphate**	PLP	B_6 vitamin
**Pyridoxine**	PN	Synthetic form of vitamin B_6
**Proline**	Pro	Amino acid
**Quinolinic acid**	QA	Tryptophan metabolite
**Riboflavin**	Ribo	Main circulating B_2 form
**Symmetric dimethylarginine**	SDMA	Marker of renal function
**Serine**	Ser	Amino acid
**Folate**	spFolate	Microbiologically active folate
**Total cysteine**	tCys	Amino acid
**Homocysteine**	tHcy	Marker of folate and B_{12} status
**Thiamine**	Thi	B_1 vitamin
**Threonine**	Thr	Amino acid
**Trimethylamineoxide**	TMAO	Choline metabolite
**Trimethyllysine**	TML	Amino acid
**Thiamine monophosphate**	TMP	B_1 vitamin
**Trigonelline**	Trig	Marker of coffee consumption
**Tryptophan**	Trp	Amino acid
**Valine**	Val	Amino acid
**Leucine**	Leu	Amino acid
**Xanthurenic acid**	XA	Tryptophan metabolite
**Kyn/Trp ratio**	KTR	Marker of immune activation
**HK/XA ratio**		Marker of B_6 status

Further, we investigated circulating concentrations of nine biomarkers related to the B-vitamins folate, riboflavin, vitamin B_6, and cobalamin, measured at diagnosis and six months post-diagnosis, in association with health-related quality of life as assessed by the EORTC QLQ-C30 questionnaire six months post-treatment in three FOCUS cohorts (ColoCare Heidelberg, EnCoRe and COLON).[35] Higher PLP concentrations were cross-sectionally associated with better physical, role, and social functioning, and reduced fatigue six months post-diagnosis. Higher HKr (3-hydroxykynurenine:(kynurenic acid+xanthurenic acid+3-hydroxyanthranilic acid+anthranilic acid)), an inverse marker of vitamin B_6 status, was cross-sectionally associated with worse global quality of life, and lower physical and role functioning. Dose-response relations were observed for PLP with global quality of life, physical, role, and social functioning. No associations were observed for changes in biomarker concentrations between diagnosis and six months with quality of life outcomes. We

For peer review only - http://bmjopen.bmj.com/site/about/guidelines.xhtml

therefore concluded that higher vitamin B_6 status was associated with better quality of life at six months post treatment and that further study is needed to clarify the role of vitamin B_6 in relation to quality of life.

Previous relevant findings from individual cohorts within the consortium

To date, individual cohorts from the FOCUS Consortium have initiated the examination of dietary supplement use and dietary habits over time. Among CRC patients enrolled in the ColoCare Study the proportion of supplement users was found to be highest post-diagnosis (35%).[36] Moreover, within an international investigation including ColoCare participants from multiple sites, Ulrich et al. showed differences in plasma folate concentration between Heidelberg and the US sites, probably reflecting variation in folic acid fortification and supplement use.[13] Furthermore, ColoCare has published data on RECQ helicase expression[37], NTRK3[38], RET[39], tumor-infiltrating lymphocytes and T cell receptor sequences[40], 25-25-hydroxyvitamin D_3[13 41], DNA methylation[42-45], miRNAs[46 47], fecal microbiota[48-50], metabolomics and transcriptomics[51-53], plasma proteins[54], gene expression[55], branched-chain amino acids[56], genetic variants[57], body composition[53 58 59], physical activity[41], and dietary patterns[4] in CRC patients. Within the COLON study, results have been published on body weight trajectories[60], changes in lifestyle[61], 25-hydroxy vitamin D levels[62 63], and inflammation markers[64] over time, as well as vitamin D[64], calcium or magnesium intake[65], physical activity[66], inflammation[67 68], skeletal muscle mass[69]/ density[70] and other measures of body composition[71] in relation to cancer recurrence, survival, or physical functioning or fatigue. Moreover, in a subset of patients undergoing chemotherapy, dietary factors in relation to chronic CIPN[72 73] as well as chemosensory perception and food preferences[74] were studied. The Austrian CORSA Study has published results on genomic data[75-77], telomere length[78], DNA repair processes[79], tumor autoantibodies[80] as well as metabolomics.[33 81] To date, publications from the EnCoRe Study have reported on associations of physical activity and sedentary behaviour[82-85], adherence to lifestyle guidelines[86], and parameters of body composition[87 88] measured through CT scans with quality of life, functioning and fatigue in CRC survivors. Recently, longitudinal associations between supplement use and fatigue were investigated from diagnosis to 2 years post-CRC treatment. No overall association between supplement use and fatigue was found but results suggest that increased levels of fatigue may be a reason for the use of supplements among CRC survivors.[89] Higher concentrations of 25OHD3 were longitudinally associated with better global quality of life and less fatigue in colorectal cancer survivors within EnCoRe suggesting a potential beneficial role of vitamin D

For peer review only - http://bmjopen.bmj.com/site/about/guidelines.xhtml

in colorectal cancer survivors.[35] In a mixed-method study using data of the EnCoRe study, colorectal cancer (treatment) related health and functioning problems negatively impacted the ability of nearly 1 in 5 long-term CRC survivors to participate in everyday life situations and their satisfaction with participation.[90] The validity of the food frequency questionnaire for measuring dietary intake among survivors of colorectal cancer within the EnCoRe study appeared to be moderate to good for most nutrients and food groups, relative to a 7-day dietary record.[91] Prediction models that we developed for estimating 1-year risk of low health-related quality of life in seven domains in colorectal cancer survivors performed well when externally validated among survivors within the EnCoRe and COLON studies.[92]

Future plans

The consortium specified a comprehensive manuscript list of future projects using data from the FOCUS Consortium. Some selected projects are described below:

a) Recently, the investigation of longitudinal associations of adherence to the dietary World Cancer Research Fund/American Institute for Cancer Research (WCRF/AICR) and Dutch Healthy Diet (DHD) recommendations with plasma kynurenine levels in colorectal cancer survivors after treatment has been finalized and the corresponding manuscript is under review at an international journal.

b) Further, near-term future plans include the investigation of (1) biomarkers related to FOCM and associations with folate intake (from diet and supplements); (2) associations between FOCM biomarkers such as vitamin B12 and tryptophan and recurrence, survival, and patient-reported outcomes in CRC; (3) the impact of folate status (FOCM biomarkers and diet/supplements) on treatment toxicity in patients treated with 5-FU modifiers; (4) the interaction between biomarkers related to FOCM and polymorphisms in FOCM-related genes in relation to CRC prognosis (recurrence & survival); (5) prognosis (disease-free and overall survival) in stage I-III CRC and associations with dietary and supplement use at diagnosis and changes during and after treatment; (6) FOCM-related biomarkers and their association with body composition in stage I to III CRC patients; (7) associations between folate status (FOCM biomarkers and diet/supplement use) and recurrence, survival and patient-reported outcomes in young-onset CRC.

c) Long-term plans include the combination of additional biomarkers measured by the individual cohorts within the next years (e.g. microbiome data, omics- and multi-omics data etc.).

For peer review only - http://bmjopen.bmj.com/site/about/guidelines.xhtml

Strengths and limitations

This is the largest consortium to date addressing the research question of folate and FOCM biomarkers in relation to survival, recurrence, treatment toxicity, and health-related quality of life outcomes in CRC patients. The cohorts included in the FOCUS Consortium are designed to enable future pooling of data.[22] For that reason, methodologies, time points and measurement instruments generally overlap, with each study presenting unique features such as additional blood collection at the 6-week follow-up time point within the EnCoRe Study and blood draws during chemotherapy within the COLON Study. The pooled sample size provides sufficient power to investigate subgroup analyses across CRC patients, e.g., within groups of patients who underwent 5-floururacil based chemotherapy or stratified by disease stage. Furthermore, the assessment of biomarkers related to FOCM are conducted at a single, state-of-the art laboratory and the biological materials are processed and stored according to standardized operation protocols across all study sites, enabling precise and accurate measurements of FOCM biomarkers. While RCTs are the gold standard for establishing causality, the FOCUS cohort with its longitudinal design can contribute to establish causal relationships, with appropriate statistical analyses. Further, the FOCUS data includes a time-varying exposure on dietary supplement intake for future studies to consider. The collection of the longitudinal data on dietary supplement intake, a key-exposure, is essential to obtain meaningful estimates and thus required for developing recommendations and guidelines regarding dietary intakes among CRC patients. The study population is predominantly based on European cohorts (90.4%) in countries that have not implemented mandatory folic acid fortification. This enables us to study a population where dietary intake and dietary supplement use determine differences in folate status, yielding information of direct relevance to cancer patients. However, the generalizability of results to populations that have introduced folic acid fortification, including the United States, might therefore also be limited.[93] Performing sensitivity analyses by excluding countries without folic acid fortification (e.g. Germany) or investigating analyses separately for Germany and the US might help to address differences in fortification status. Moreover, patients were predominantly White, thus, it is not possible to address racial and ethnic minorities. Ethnicity/race is an important determinant of folate status and metabolism may be different between African Americans and Hispanics[94], thus, recommendations should be limited to this current population. Future studies are warranted in diverse populations and compared with the FOCUS cohort.

Another limitation of the consortium includes differences in collection strategies of the baseline study time point across included studies: CORSA, COLON and EnCoRe are

For peer review only - http://bmjopen.bmj.com/site/about/guidelines.xhtml

recruiting CRC patients at diagnosis, preferably prior to any cancer treatment while ColoCare is recruiting patients prior to surgery (i.e. neoadjuvant treatment might have occurred prior to baseline blood and data collection). Nevertheless, the ColoCare protocol requires that no blood draw occurs within 2 weeks of neoadjuvant or adjuvant chemotherapy, limiting the influence of ongoing treatments on blood biomarkers. In some cohorts, the timing with respect to adjuvant chemotherapy is less clearly defined and this will be carefully considered in sensitivity analyses. Furthermore, survivor bias – a type of selection bias – may be introduced as some patients died or did not complete follow-up questionnaires or did not provide blood samples. It is possible that patients who experience more severe toxicities, worse clinical outcomes, or worse health-related quality of life, are underrepresented among those patients who completed follow-up measurements.[4] Cohort studies such as the one presented here generate critical knowledge about preventable causes of disease. However, selection bias may affect estimates. This is particularly true for non-participation at follow-up that may depend on both the exposure and outcome. Within a review, Nohr et al. showed a range of methods to quantify and adjust for selection bias. Even with limited data on nonparticipants and those lost to follow up, it is possible to examine how effect estimates in a specific study may be biased by selection.[95] The likelihood for reverse causation is small in this prospective cohort, as the exposure measurements (blood folate levels and intake through diet/supplements) were collected before the outcome (survival, recurrence, and quality of life) occurred. Therefore, these outcomes are unlikely to have influenced the exposure measurements. Given the robust follow-up in these cohorts for outcomes and data availability, future studies will be able to consider key confounders as well as predictors of recurrence and survival.

Collaboration

A substantial amount of time has been spent in creating the harmonized dataset of baseline variables from cohorts within the FOCUS Consortium, with follow-up data collection and FOCM biomarker analyses. Any person interested in collaborating, learning about the FOCUS Consortium or in getting access to FOCUS data and in-depth analyses can contact the coordinating study PIs Prof. Andrea Gsur (andrea.gsur@meduniwien.ac.at) and Prof. Cornelia Ulrich (neli.ulrich@hci.utah.edu). Requests for data will be discussed and decided by all PIs and will require a Data Transfer Agreement.

For peer review only - http://bmjopen.bmj.com/site/about/guidelines.xhtml

Acknowledgements

ColoCare Heidelberg: We thank all ColoCare study participants, the liquid biobank of the National Center for Tumor Diseases for storage of the blood samples according to the SOPs of the Biomaterialbank Heidelberg (BMBH), Michaela Magenreuter, Maria Thomalla-Starzl, Mareen, Dupovac, Marzena Knyssok-Sypniewski, Lin Zielske, Anett Brendel, Renate Skatula, and Marita Wenzel for laboratory work, Rifraz Farook, Dr. Werner Diehl and Inna Müller for data management and clinical follow-up data collection, Dr. Clare Abbenhardt-Martin, Susanne Jakob, Judith Kammer for patient recruitment and follow-up, and Dr. Petra Schrotz-King and Dr. Jürgen Boehm for study coordination.

ColoCare Huntsman Cancer Institute: We thank all ColoCare study participants, the biorepository at Huntsman Cancer Institute for storage of the blood samples according to standardized SOPs, and Christy Warby for laboratory work. We thank Tyler Farr, Sarah Fischbuch and Debra Ma for data management and clinical follow-up data collection. We are very grateful for patient recruitment and follow-up by Elisa Santori, Hanna Omar-Payne and Marissa Grande, and Dr. Jürgen Boehm for study coordination.

ColoCare Fred Hutchinson Cancer Research Center: We thank all ColoCare study participants, the biorepository at Fred Hutchinson Cancer Research Center for storage of the blood samples according to standardized SOPs and the laboratory team for sample processing. We thank the ColoCare team for patient recruitment and follow-up and data management.

EnCoRe: We would like to thank all participants of the EnCoRe study and the health professionals in the three hospitals involved in the recruitment of participants of the study: Maastricht University Medical Center+, VieCuri Medical Center, and Zuyderland Medical Center. We would also like to thank the MEMIC center for data and information management for facilitating the logistic processes and data management of our study. Finally, we would like to thank the research dieticians and research assistant who are responsible for patient inclusion and follow-up, performing home visits, as well as data collection and processing.

CORSA: We kindly thank all individuals who agreed to participate in the CORSA study. Furthermore, we thank Thomas Bachleitner-Hofmann, Michael M. Bergmann, Judith Karner-Hanusch and Anton Stift (Department of Surgery, Medical University of Vienna), Karl Mach, Azita Deutinger-Permoon (KRAGES, Austria), and their co-workers for supporting CORSA recruitment. In addition, we would like to acknowledge Elisabeth Feik, Philipp Hofer and Cornelia Zöchmeister, for patient recruitment, sample processing, and follow-up.

For peer review only

For peer review only - http://bmjopen.bmj.com/site/about/guidelines.xhtml

COLON: The authors would like to thank the COLON participants, COLON investigators at Wageningen University & Research and the involved co-workers in the participating hospitals.

Contributors

BG, MW, EK, PMU, CMU, AG, ABU, NH: conceived of the project, developed the overall research plan, and provided study oversight; BG, EHvR, ANH, SB, AJMRG, AU, JO, JLK, VD, RK, TG, TL, GK, TK, DEK, FJBvD, MJLB, AB, CIL, WMG, KV: conducted hands-on experiments and data collection; BG, EHvR, ANH, SB, AJMRG, AU, JO, JLK, VD, RK, TG, TL, GK, TK, DEK, FJBvD, CIL, WMG, KV, CMU: provided essential reagents or essential materials, databases, and so forth, necessary for the research; BG, EHvR, MJLB, AB, ANH, VD, SB: analyzed the data or performed the statistical analysis and made a major contribution to writing the paper; BG, MS, EK, PMU, ABU, MW, AG, CMU: had primary responsibility for the final content; all authors: reviewed the manuscript critically, provided feedback, and read and approved the final manuscript.

Funding

The FOCUS Consortium is an ERA-NET (European Research Area Network) on Translational Cancer Research (TRANSCAN) project funded by the local research funding agencies (German Ministry of Education and Research, Germany, 01KT1503; Dutch Cancer Society with grant number UW2014-6877, the Netherlands; FWF Austrian Science Fund, Austria (API02104FW); Research Council Norway/Norwegian Cancer Society (RNC), Norway). The COLON study is further sponsored by Wereld Kanker Onderzoek Fonds, including funds from grant 2014/1179 as part of the World Cancer Research Fund International Regular Grant Programme; Alpe d'Huzes/Dutch Cancer Society (UM 2012-5653, UW 2013-5927, UW 2015-7946); and ERA-NET on Translational Cancer Research (TRANSCAN/Dutch Cancer Society: UW2013-6397 and the Netherlands Organization for Health Research and Development (ZonMw), the Netherlands). The EnCoRe study was supported by grants from the Stichting Alpe d'HuZes within the research program 'Leven met kanker' of the Dutch Cancer Society (Grant No. UM-2010-4867 and UM-2012-5653), and by grants from Kankeronderzoekfonds Limburg as part of Health Foundation Limburg (Grant No. 00005739), and by a grant from Wereld Kanker Onderzoek Fonds (WKOF), as part of the World Cancer Research Fund International grant programme (grant number 2016/1620).

For peer review only - http://bmjopen.bmj.com/site/about/guidelines.xhtml

Biljana Gigic was funded by the ERA-NET on Translational Cancer Research (TRANSCAN), the German Ministry of Education and Research project 01KT1503, the National Institutes of Health/National Cancer Institute (NHI/NCI) projects R01 CA189184 and U01 CA206110, the Stiftung LebensBlicke, and the Matthias-Lackas Foundations. Eline H. van Roekel was funded by the Wereld Kanker Onderzoek Fonds (WKOF), part of the World Cancer Research Fund International grant programme (grant number 2016/1620). Martijn Bours was funded by Alpe d'HuZes within the research program 'Leven met kanker' of the Dutch Cancer Society (Grant No. UM-2012-5653). Dieuwertje E. Kok is supported by a Veni grant of the Netherlands Organisation for Scientific Research (grant no. 016.Veni.188.082). Andreana N. Holowatyj was supported by the National Institutes of Health under Ruth L. Kirschstein National Research Service Award T32 HG008962 from the National Human Genome Research Institute. Nina Habermann was funded by the TRANSCAN project 01KT1512, Cornelia M. Ulrich and Jennifer Ose were funded by the Huntsman Cancer Foundation, and NIH/NCI projects R01 CA189184, R01 CA207371, U01 CA206110, U01 CA206110 and P30 CA042014. NIH grants (P30 CA15704, U01 CA152756, R01 CA194663, R01 CA220004, P01 CA077852), Rodger C. Haggitt Endowed Chair, R.A.C.E. Charities, Cottrell Family Fund, Listwin Family Foundation, Seattle Translational Tumor Research program, Fred Hutchinson Cancer Research Center (William M. Grady). Stefanie Brezina was funded by the ERA-NET on Translational Cancer Research (TRANSCAN) project FOCUS I2104-B26 and the ERA-NET on Translational Cancer Research (TRANSCAN) project MetaboCCC I 1578 – B19. J.L. Koole was supported by Kankeronderzoekfonds Limburg as part of Health Foundation Limburg (Grant No. 00005739). Victoria Damerell was funded by the German Ministry of Education and Research project (01KD2101D).

Disclaimer

The funder had no role in the design of the FOCUS consortium, or conduct of the study, collection, management, analysis, and interpretation of the data, preparation, review, or approval of the manuscript, or decision to submit the manuscript for publication.

Competing interests

None declared.

Patient consent for publication

Not applicable.

For peer review only

Ethics approval

The Heidelberg ColoCare Study was approved by the Ethics Committee of the Medical Faculty of Heidelberg (approval no. S-310/2001 and S-134/2016). The ColoCare Study at the Huntsman Cancer Institute and the Fred Hutchinson Cancer Research Center were approved by the respective IRBs (#77147 and #6407). For the CORSA study all subjects gave written informed consent and the study was approved by the institutional review boards: 'Ethikkommission Burgenland' (33/2010), by the ethical review committee of the Medical University of Vienna (1160/2016) and by the "Ethikkommission der Stadt Wien" (EK 06-150VK). The EnCoRe Study was approved by the Medical Ethics Committee of the Maastricht University Hospital and Maastricht University (METC 11-3-075).

Data availability

Data described in the manuscript, code book, and analytic code have been generated from European-based consortia and as such are subject to regulations from multiple European countries, which limit our availability to share data. The consortium's funding has ended, and no centralized staff is available to support

data requests. However, the FOCUS PIs have agreed to answer any queries or discuss potential projects with anyone interested in future collaborative research. For further questions please contact colocarestudy_admin@hci.utah.edu.

For peer review only

References

1. Ferlay J, Colombet M, Soerjomataram I, et al. Estimating the global cancer incidence and mortality in 2018: GLOBOCAN sources and methods. *International journal of cancer Journal international du cancer* 2019;144(8):1941-53. doi: 10.1002/ijc.31937 [published Online First: 2018/10/24]
2. De Angelis R, Sant M, Coleman MP, et al. Cancer survival in Europe 1999-2007 by country and age: results of EUROCARE--5-a population-based study. *The Lancet Oncology* 2014;15(1):23-34. doi: 10.1016/S1470-2045(13)70546-1
3. Ferlay J, Colombet M, Soerjomataram I, et al. Cancer incidence and mortality patterns in Europe: Estimates for 40 countries and 25 major cancers in 2018. *European journal of cancer* 2018;103:356-87. doi: 10.1016/j.ejca.2018.07.005 [published Online First: 2018/08/14]
4. Gigic B, Boeing H, Toth R, et al. Associations Between Dietary Patterns and Longitudinal Quality of Life Changes in Colorectal Cancer Patients: The ColoCare Study. *Nutrition and cancer* 2017:1-10. doi: 10.1080/01635581.2018.1397707 [published Online First: 2017/12/16]
5. Velicer CM, Ulrich CM. Vitamin and mineral supplement use among US adults after cancer diagnosis: a systematic review. *Journal of clinical oncology : official journal of the American Society of Clinical Oncology* 2008;26(4):665-73. doi: 10.1200/JCO.2007.13.5905
6. Ulrich CM, Potter JD. Folate supplementation: too much of a good thing? *Cancer Epidemiol Biomarkers Prev* 2006;15(2):189-93. doi: 10.1158/1055-9965.EPI-152CO [published Online First: 2006/02/24]
7. Jatoi A, Williams B, Nichols F, et al. Is voluntary vitamin and mineral supplementation associated with better outcome in non-small cell lung cancer patients? Results from the Mayo Clinic lung cancer cohort. *Lung Cancer* 2005;49(1):77-84. doi: 10.1016/j.lungcan.2005.01.004 [published Online First: 2005/06/14]
8. Ferrucci LM, McCorkle R, Smith T, et al. Factors related to the use of dietary supplements by cancer survivors. *J Altern Complement Med* 2009;15(6):673-80. doi: 10.1089/acm.2008.0387
9. Caudill MA. Folate bioavailability: implications for establishing dietary recommendations and optimizing status. *The American journal of clinical nutrition* 2010;91(5):1455S-60S. doi: 10.3945/ajcn.2010.28674E [published Online First: 2010/03/12]
10. Ducker GS, Rabinowitz JD. One-Carbon Metabolism in Health and Disease. *Cell Metab* 2017;25(1):27-42. doi: 10.1016/j.cmet.2016.08.009 [published Online First: 2016/09/20]
11. Miller JW, Ulrich CM. Folic acid and cancer--where are we today? *Lancet* 2013;381(9871):974-6. doi: 10.1016/S0140-6736(13)60110-5 [published Online First: 2013/01/29]
12. Kim YI. Folate and colorectal cancer: an evidence-based critical review. *Mol Nutr Food Res* 2007;51(3):267-92. doi: 10.1002/mnfr.200600191 [published Online First: 2007/02/14]
13. Ulrich CM, Toriola AT, Siegel EM, et al. Plasma 25-hydroxyvitamin D3, folate and vitamin B12 biomarkers among international colorectal cancer patients: a pilot study. *J Nutr Sci* 2013;2:e9. doi: 10.1017/jns.2012.28
14. Cole BF, Baron JA, Sandler RS, et al. Folic acid for the prevention of colorectal adenomas: a randomized clinical trial. *Jama* 2007;297(21):2351-9. doi: 10.1001/jama.297.21.2351 [published Online First: 2007/06/07]
15. Lawrance AK, Deng L, Rozen R. Methylenetetrahydrofolate reductase deficiency and low dietary folate reduce tumorigenesis in Apc min/+ mice. *Gut* 2009;58(6):805-11. doi: 10.1136/gut.2007.143107 [published Online First: 2009/01/29]
16. Lindzon GM, Medline A, Sohn KJ, et al. Effect of folic acid supplementation on the progression of colorectal aberrant crypt foci. *Carcinogenesis* 2009;30(9):1536-43. doi: 10.1093/carcin/bgp152 [published Online First: 2009/06/23]
17. Mason JB. Folate, cancer risk, and the Greek god, Proteus: a tale of two chameleons. *Nutrition reviews* 2009;67(4):206-12. doi: 10.1111/j.1753-4887.2009.00190.x [published Online First: 2009/04/02]
18. Ulrich CM, Miller JW. Folate and health: present and future. *Mol Nutr Food Res* 2013;57(4):561. doi: 10.1002/mnfr.201370034 [published Online First: 2013/04/05]
19. Choi JH, Yates Z, Martin C, et al. Gene-Nutrient Interaction between Folate and Dihydrofolate Reductase in Risk for Adenomatous Polyp Occurrence: A Preliminary Report. *J Nutr Sci*

For peer review only - http://bmjopen.bmj.com/site/about/guidelines.xhtml

Vitaminol (Tokyo) 2015;61(6):455-9. doi: 10.3177/jnsv.61.455 [published Online First: 2016/02/16]
20. Sawaengsri H, Wang J, Reginaldo C, et al. High folic acid intake reduces natural killer cell cytotoxicity in aged mice. *J Nutr Biochem* 2016;30:102-7. doi: 10.1016/j.jnutbio.2015.12.006 [published Online First: 2016/03/26]
21. Ulrich CM, Gigic B, Bohm J, et al. The ColoCare Study: A Paradigm of Transdisciplinary Science in Colorectal Cancer Outcomes. *Cancer Epidemiol Biomarkers Prev* 2019;28(3):591-601. doi: 10.1158/1055-9965.EPI-18-0773 [published Online First: 2018/12/14]
22. Winkels RM, Heine-Broring RC, van Zutphen M, et al. The COLON study: Colorectal cancer: Longitudinal, Observational study on Nutritional and lifestyle factors that may influence colorectal tumour recurrence, survival and quality of life. *BMC cancer* 2014;14:374. doi: 10.1186/1471-2407-14-374 [published Online First: 2014/06/03]
23. van Roekel EH, Bours MJ, de Brouwer CP, et al. The applicability of the international classification of functioning, disability, and health to study lifestyle and quality of life of colorectal cancer survivors. *Cancer Epidemiol Biomarkers Prev* 2014;23(7):1394-405. doi: 10.1158/1055-9965.EPI-13-1144 [published Online First: 2014/05/08]
24. Fayers P, Bottomley A, Group EQoL, et al. Quality of life research within the EORTC-the EORTC QLQ-C30. European Organisation for Research and Treatment of Cancer. *European journal of cancer* 2002;38 Suppl 4:S125-33.
25. Postma TJ, Aaronson NK, Heimans JJ, et al. The development of an EORTC quality of life questionnaire to assess chemotherapy-induced peripheral neuropathy: the QLQ-CIPN20. *European journal of cancer* 2005;41(8):1135-9. doi: 10.1016/j.ejca.2005.02.012
26. Molloy AM, Scott JM. Microbiological assay for serum, plasma, and red cell folate using cryopreserved, microtiter plate method. *Methods Enzymol* 1997;281:43-53. [published Online First: 1997/01/01]
27. Kelleher BP, Broin SD. Microbiological assay for vitamin B12 performed in 96-well microtitre plates. *J Clin Pathol* 1991;44(7):592-5. [published Online First: 1991/07/01]
28. Hannisdal R, Ueland PM, Svardal A. Liquid chromatography-tandem mass spectrometry analysis of folate and folate catabolites in human serum. *Clin Chem* 2009;55(6):1147-54. doi: 10.1373/clinchem.2008.114389 [published Online First: 2009/04/11]
29. Midttun O, Hustad S, Ueland PM. Quantitative profiling of biomarkers related to B-vitamin status, tryptophan metabolism and inflammation in human plasma by liquid chromatography/tandem mass spectrometry. *Rapid Commun Mass Spectrom* 2009;23(9):1371-9. doi: 10.1002/rcm.4013 [published Online First: 2009/04/02]
30. Midttun O, Kvalheim G, Ueland PM. High-throughput, low-volume, multianalyte quantification of plasma metabolites related to one-carbon metabolism using HPLC-MS/MS. *Anal Bioanal Chem* 2013;405(6):2009-17. doi: 10.1007/s00216-012-6602-6 [published Online First: 2012/12/13]
31. Midttun O, McCann A, Aarseth O, et al. Combined Measurement of 6 Fat-Soluble Vitamins and 26 Water-Soluble Functional Vitamin Markers and Amino Acids in 50 muL of Serum or Plasma by High-Throughput Mass Spectrometry. *Anal Chem* 2016;88(21):10427-36. doi: 10.1021/acs.analchem.6b02325 [published Online First: 2016/11/02]
32. Meyer K, Ueland PM. Targeted quantification of C-reactive protein and cystatin c and its variants by immuno-MALDI-MS. *Anal Chem* 2014;86(12):5807-14. doi: 10.1021/ac500704y [published Online First: 2014/05/23]
33. Geijsen A, Ulvik A, Gigic B, et al. Circulating Folate and Folic Acid Concentrations: Associations With Colorectal Cancer Recurrence and Survival. *JNCI Cancer Spectr* 2020;4(5):pkaa051. doi: 10.1093/jncics/pkaa051 [published Online First: 2020/11/03]
34. Kiblawi R, Holowatyj AN, Gigic B, et al. One-carbon metabolites, B-vitamins and associations with systemic inflammation and angiogenesis biomarkers among colorectal cancer patients: results from the ColoCare Study. *The British journal of nutrition* 2020:1-32. doi: 10.1017/S0007114520000422 [published Online First: 2020/02/06]
35. Koole JL, Bours MJL, Geijsen A, et al. Circulating B-vitamin biomarkers and B-vitamin supplement use in relation to quality of life in patients with colorectal cancer: results from the FOCUS consortium. *The American journal of clinical nutrition* 2021 doi: 10.1093/ajcn/nqaa422 [published Online First: 2021/03/06]

For peer review only - http://bmjopen.bmj.com/site/about/guidelines.xhtml

36. Schrotz-King P, Dölp K, Paskow MJ, et al. Dietary Supplement Use among German Colorectal Cancer Patients: the ColoCare Study: AACR/MEG Post-GWAS Horizons in Molecular Epidemiology: Digging Deeper into the Environment., 2012.
37. Lao VV, Welcsh P, Luo Y, et al. Altered RECQ Helicase Expression in Sporadic Primary Colorectal Cancers. *Transl Oncol* 2013;6(4):458-69.
38. Luo Y, Kaz AM, Kanngurn S, et al. NTRK3 is a potential tumor suppressor gene commonly inactivated by epigenetic mechanisms in colorectal cancer. *PLoS Genet* 2013;9(7):e1003552. doi: 10.1371/journal.pgen.1003552
39. Luo Y, Tsuchiya KD, Il Park D, et al. RET is a potential tumor suppressor gene in colorectal cancer. *Oncogene* 2013;32(16):2037-47. doi: 10.1038/onc.2012.225
40. Sherwood AM, Emerson RO, Scherer D, et al. Tumor-infiltrating lymphocytes in colorectal tumors display a diversity of T cell receptor sequences that differ from the T cells in adjacent mucosal tissue. *Cancer Immunol Immunother* 2013;62(9):1453-61. doi: 10.1007/s00262-013-1446-2
41. Skender S, Bohm J, Schrotz-King P, et al. Plasma 25-Hydroxyvitamin D3 Levels in Colorectal Cancer Patients and Associations with Physical Activity. *Nutrition and cancer* 2017;69(2):229-37. doi: 10.1080/01635581.2017.1265131
42. Luo Y, Wong CJ, Kaz AM, et al. Differences in DNA methylation signatures reveal multiple pathways of progression from adenoma to colorectal cancer. *Gastroenterology* 2014;147(2):418-29 e8. doi: 10.1053/j.gastro.2014.04.039
43. Barrow TM, Klett H, Toth R, et al. Smoking is associated with hypermethylation of the APC 1A promoter in colorectal cancer: the ColoCare Study. *J Pathol* 2017;243(3):366-75. doi: 10.1002/path.4955 [published Online First: 2017/08/10]
44. Yu M, Carter KT, Makar KW, et al. MethyLight droplet digital PCR for detection and absolute quantification of infrequently methylated alleles. *Epigenetics* 2015;10(9):803-9. doi: 10.1080/15592294.2015.1068490
45. Klett H, Balavarca Y, Toth R, et al. Robust prediction of gene regulation in colorectal cancer tissues from DNA methylation profiles. *Epigenetics* 2018;13(4):386-97. doi: 10.1080/15592294.2018.1460034 [published Online First: 2018/04/27]
46. Ristau J, Staffa J, Schrotz-King P, et al. Suitability of circulating miRNAs as potential prognostic markers in colorectal cancer. *Cancer Epidemiol Biomarkers Prev* 2014;23(12):2632-7. doi: 10.1158/1055-9965.EPI-14-0556
47. Yuan Z, Baker K, Redman MW, et al. Dynamic plasma microRNAs are biomarkers for prognosis and early detection of recurrence in colorectal cancer. *British journal of cancer* 2017;117(8):1202-10. doi: 10.1038/bjc.2017.266 [published Online First: 2017/08/16]
48. Zeller G, Tap J, Voigt AY, et al. Potential of fecal microbiota for early-stage detection of colorectal cancer. *Mol Syst Biol* 2014;10:766. doi: 10.15252/msb.20145645
49. Thomas AM, Manghi P, Asnicar F, et al. Metagenomic analysis of colorectal cancer datasets identifies cross-cohort microbial diagnostic signatures and a link with choline degradation. *Nat Med* 2019;25(4):667-78. doi: 10.1038/s41591-019-0405-7 [published Online First: 2019/04/03]
50. Wirbel J, Pyl PT, Kartal E, et al. Meta-analysis of fecal metagenomes reveals global microbial signatures that are specific for colorectal cancer. *Nat Med* 2019;25(4):679-89. doi: 10.1038/s41591-019-0406-6 [published Online First: 2019/04/03]
51. Liesenfeld DB, Grapov D, Fahrmann JF, et al. Metabolomics and transcriptomics identify pathway differences between visceral and subcutaneous adipose tissue in colorectal cancer patients: the ColoCare study. *The American journal of clinical nutrition* 2015;102(2):433-43. doi: 10.3945/ajcn.114.103804
52. Liesenfeld DB, Habermann N, Toth R, et al. Changes in urinary metabolic profiles of colorectal cancer patients enrolled in a prospective cohort study (ColoCare). *Metabolomics* 2015;11(4):998-1012. doi: 10.1007/s11306-014-0758-3 [published Online First: 2015/08/01]
53. Ose J, Gigic B, Lin T, et al. Multiplatform Urinary Metabolomics Profiling to Discriminate Cachectic from Non-Cachectic Colorectal Cancer Patients: Pilot Results from the ColoCare Study. *Metabolites* 2019;9(9) doi: 10.3390/metabo9090178 [published Online First: 2019/09/11]

For peer review only - http://bmjopen.bmj.com/site/about/guidelines.xhtml

54. Bohm J, Pianka F, Stuttgen N, et al. Discovery of novel plasma proteins as biomarkers for the development of incisional hernias after midline incision in patients with colorectal cancer: The ColoCare study. *Surgery* 2017;161(3):808-17. doi: 10.1016/j.surg.2016.08.025
55. Gong J, Hutter CM, Newcomb PA, et al. Genome-Wide Interaction Analyses between Genetic Variants and Alcohol Consumption and Smoking for Risk of Colorectal Cancer. *PLoS Genet* 2016;12(10):e1006296. doi: 10.1371/journal.pgen.1006296 [published Online First: 2016/10/11]
56. Delphan M, Lin T, Liesenfeld DB, et al. Associations of branched-chain amino acids with parameters of energy balance and survival in colorectal cancer patients: Results from the ColoCare Study. *Metabolomics* 2018;2018(14) doi: 10.1007/s11306-017-1314-8 [published Online First: 2018/05/01]
57. Rosenthal EA, Shirts BH, Amendola LM, et al. Rare loss of function variants in candidate genes and risk of colorectal cancer. *Hum Genet* 2018 doi: 10.1007/s00439-018-1938-4 [published Online First: 2018/09/30]
58. Nattenmueller J, Hoegenauer H, Boehm J, et al. CT-based compartmental quantification of adipose tissue versus body metrics in colorectal cancer patients. *Eur Radiol* 2016;26(11):4131-40. doi: 10.1007/s00330-016-4231-8
59. Ozoya OO, Siegel EM, Srikumar T, et al. Quantitative Assessment of Visceral Obesity and Postoperative Colon Cancer Outcomes. *J Gastrointest Surg* 2017;21(3):534-42. doi: 10.1007/s11605-017-3362-9 [published Online First: 2017/01/20]
60. van Zutphen M, Geelen A, Boshuizen HC, et al. Pre-to-post diagnosis weight trajectories in colorectal cancer patients with non-metastatic disease. *Supportive care in cancer : official journal of the Multinational Association of Supportive Care in Cancer* 2019;27(4):1541-49. doi: 10.1007/s00520-018-4560-z [published Online First: 2018/11/30]
61. van Zutphen M, Boshuizen HC, Kok DE, et al. Colorectal cancer survivors only marginally change their overall lifestyle in the first 2 years following diagnosis. *J Cancer Surviv* 2019;13(6):956-67. doi: 10.1007/s11764-019-00812-7 [published Online First: 2019/10/28]
62. Wesselink E, Bours MJL, de Wilt JHW, et al. Chemotherapy and vitamin D supplement use are determinants of serum 25-hydroxyvitamin D levels during the first six months after colorectal cancer diagnosis. *J Steroid Biochem Mol Biol* 2020;199:105577. doi: 10.1016/j.jsbmb.2020.105577 [published Online First: 2020/01/10]
63. Wesselink E, Kok DE, de Wilt JHW, et al. Sufficient 25-Hydroxyvitamin D Levels 2 Years after Colorectal Cancer Diagnosis are Associated with a Lower Risk of All-cause Mortality. *Cancer Epidemiol Biomarkers Prev* 2021;30(4):765-73. doi: 10.1158/1055-9965.EPI-20-1388 [published Online First: 2021/02/04]
64. Wesselink E, Balvers M, Bours MJL, et al. The association between circulating levels of vitamin D and inflammatory markers in the first 2 years after colorectal cancer diagnosis. *Therap Adv Gastroenterol* 2020;13:1756284820923922. doi: 10.1177/1756284820923922 [published Online First: 2020/06/18]
65. Wesselink E, Kok DE, Bours MJL, et al. Vitamin D, magnesium, calcium, and their interaction in relation to colorectal cancer recurrence and all-cause mortality. *The American journal of clinical nutrition* 2020;111(5):1007-17. doi: 10.1093/ajcn/nqaa049 [published Online First: 2020/03/20]
66. van Zutphen M, Winkels RM, van Duijnhoven FJ, et al. An increase in physical activity after colorectal cancer surgery is associated with improved recovery of physical functioning: a prospective cohort study. *BMC cancer* 2017;17(1):74. doi: 10.1186/s12885-017-3066-2 [published Online First: 2017/01/27]
67. Wesselink E, van Baar H, van Zutphen M, et al. Inflammation Is a Mediating Factor in the Association between Lifestyle and Fatigue in Colorectal Cancer Patients. *Cancers (Basel)* 2020;12(12) doi: 10.3390/cancers12123701 [published Online First: 2020/12/16]
68. Wesselink E, Staritsky LE, van Zutphen M, et al. The association between the adapted dietary inflammatory index and colorectal cancer recurrence and all-cause mortality. *Clin Nutr* 2021;40(6):4436-43. doi: 10.1016/j.clnu.2021.01.004 [published Online First: 2021/01/23]
69. van Baar H, Winkels RM, Brouwer JGM, et al. Associations of Abdominal Skeletal Muscle Mass, Fat Mass, and Mortality among Men and Women with Stage I-III Colorectal Cancer. *Cancer*

For peer review only - http://bmjopen.bmj.com/site/about/guidelines.xhtml

Epidemiol Biomarkers Prev 2020;29(5):956-65. doi: 10.1158/1055-9965.EPI-19-1134 [published Online First: 2020/03/07]

70. van Baar H, Beijer S, Bours MJL, et al. Low radiographic muscle density is associated with lower overall and disease-free survival in early-stage colorectal cancer patients. *J Cancer Res Clin Oncol* 2018;144(11):2139-47. doi: 10.1007/s00432-018-2736-z [published Online First: 2018/08/19]
71. van Baar H, Bours MJL, Beijer S, et al. Body composition and its association with fatigue in the first 2 years after colorectal cancer diagnosis. *J Cancer Surviv* 2021;15(4):597-606. doi: 10.1007/s11764-020-00953-0 [published Online First: 2020/10/18]
72. Winkels RM, van Brakel L, van Baar H, et al. Are Ergothioneine Levels in Blood Associated with Chronic Peripheral Neuropathy in Colorectal Cancer Patients Who Underwent Chemotherapy? *Nutrition and cancer* 2020;72(3):451-59. doi: 10.1080/01635581.2019.1637005 [published Online First: 2019/07/13]
73. Wesselink E, Winkels RM, van Baar H, et al. Dietary Intake of Magnesium or Calcium and Chemotherapy-Induced Peripheral Neuropathy in Colorectal Cancer Patients. *Nutrients* 2018;10(4) doi: 10.3390/nu10040398 [published Online First: 2018/03/24]
74. Postma EM, Kok DE, de Graaf C, et al. Chemosensory perception and food preferences in colorectal cancer patients undergoing adjuvant chemotherapy. *Clin Nutr ESPEN* 2020;40:242-51. doi: 10.1016/j.clnesp.2020.09.012 [published Online First: 2020/11/14]
75. Hofer P, Hagmann M, Brezina S, et al. Bayesian and frequentist analysis of an Austrian genome-wide association study of colorectal cancer and advanced adenomas. *Oncotarget* 2017;8(58):98623-34. doi: 10.18632/oncotarget.21697 [published Online First: 2017/12/13]
76. Schmit SL, Edlund CK, Schumacher FR, et al. Novel Common Genetic Susceptibility Loci for Colorectal Cancer. *Journal of the National Cancer Institute* 2019;111(2):146-57. doi: 10.1093/jnci/djy099 [published Online First: 2018/06/20]
77. Schafmayer C, Harrison JW, Buch S, et al. Genome-wide association analysis of diverticular disease points towards neuromuscular, connective tissue and epithelial pathomechanisms. *Gut* 2019 doi: 10.1136/gutjnl-2018-317619 [published Online First: 2019/01/21]
78. Zochmeister C, Brezina S, Hofer P, et al. Leukocyte telomere length throughout the continuum of colorectal carcinogenesis. *Oncotarget* 2018;9(17):13582-92. doi: 10.18632/oncotarget.24431 [published Online First: 2018/03/24]
79. Jiraskova K, Hughes DJ, Brezina S, et al. Functional Polymorphisms in DNA Repair Genes Are Associated with Sporadic Colorectal Cancer Susceptibility and Clinical Outcome. *Int J Mol Sci* 2018;20(1) doi: 10.3390/ijms20010097 [published Online First: 2018/12/29]
80. Luna Coronell JA, Sergelen K, Hofer P, et al. The Immunome of Colon Cancer: Functional In Silico Analysis of Antigenic Proteins Deduced from IgG Microarray Profiling. *Genomics Proteomics Bioinformatics* 2018;16(1):73-84. doi: 10.1016/j.gpb.2017.10.002 [published Online First: 2018/03/06]
81. Geijsen A, van Roekel EH, van Duijnhoven FJB, et al. Plasma metabolites associated with colorectal cancer stage: Findings from an international consortium. *International journal of cancer Journal international du cancer* 2019 doi: 10.1002/ijc.32666 [published Online First: 2019/09/10]
82. Van Roekel EH, Bours MJ, Breedveld-Peters JJ, et al. Light Physical Activity Is Associated with Quality of Life after Colorectal Cancer. *Med Sci Sports Exerc* 2015;47(12):2493-503. doi: 10.1249/MSS.0000000000000698 [published Online First: 2015/05/15]
83. van Roekel EH, Winkler EA, Bours MJ, et al. Associations of sedentary time and patterns of sedentary time accumulation with health-related quality of life in colorectal cancer survivors. *Prev Med Rep* 2016;4:262-9. doi: 10.1016/j.pmedr.2016.06.022 [published Online First: 2016/07/16]
84. van Roekel EH, Bours MJ, Breedveld-Peters JJ, et al. Modeling how substitution of sedentary behavior with standing or physical activity is associated with health-related quality of life in colorectal cancer survivors. *Cancer causes & control : CCC* 2016;27(4):513-25. doi: 10.1007/s10552-016-0725-6 [published Online First: 2016/02/20]
85. van Roekel EH, Duchateau J, Bours MJL, et al. Longitudinal associations of light-intensity physical activity with quality of life, functioning and fatigue after colorectal cancer. *Quality of life research : an international journal of quality of life aspects of treatment, care and*

For peer review only - http://bmjopen.bmj.com/site/about/guidelines.xhtml

rehabilitation 2020;29(11):2987-98. doi: 10.1007/s11136-020-02566-7 [published Online First: 2020/07/04]

86. Breedveld-Peters JJL, Koole JL, Muller-Schulte E, et al. Colorectal cancers survivors' adherence to lifestyle recommendations and cross-sectional associations with health-related quality of life. *The British journal of nutrition* 2018;120(2):188-97. doi: 10.1017/S0007114518000661 [published Online First: 2018/04/17]
87. van Roekel EH, Bours MJL, Te Molder MEM, et al. Associations of adipose and muscle tissue parameters at colorectal cancer diagnosis with long-term health-related quality of life. *Quality of life research : an international journal of quality of life aspects of treatment, care and rehabilitation* 2017;26(7):1745-59. doi: 10.1007/s11136-017-1539-z [published Online First: 2017/03/21]
88. van Heinsbergen M, Konsten JL, Bours MJL, et al. Preoperative handgrip strength is not associated with complications and health-related quality of life after surgery for colorectal cancer. *Sci Rep* 2020;10(1):13005. doi: 10.1038/s41598-020-69806-1 [published Online First: 2020/08/05]
89. Koole JL, Bours MJL, Breedveld-Peters JJL, et al. Is dietary supplement use longitudinally associated with fatigue in stage I-III colorectal cancer survivors? *Clin Nutr* 2019 doi: 10.1016/j.clnu.2018.12.037 [published Online First: 2019/02/17]
90. Breedveld-Peters JJL, Bours MJL, Cords CI, et al. The impact of participation restrictions on everyday life in long-term colorectal cancer survivors in the EnCoRe study: A mixed-method study. *Eur J Oncol Nurs* 2020;45:101724. doi: 10.1016/j.ejon.2020.101724 [published Online First: 2020/02/12]
91. Koole JL, Bours MJL, Breedveld-Peters JJL, et al. Evaluating the Validity of a Food Frequency Questionnaire in Comparison with a 7-Day Dietary Record for Measuring Dietary Intake in a Population of Survivors of Colorectal Cancer. *J Acad Nutr Diet* 2020;120(2):245-57. doi: 10.1016/j.jand.2019.09.008 [published Online First: 2019/12/07]
92. Revesz D, van Kuijk SMJ, Mols F, et al. Development and internal validation of prediction models for colorectal cancer survivors to estimate the 1-year risk of low health-related quality of life in multiple domains. *BMC Med Inform Decis Mak* 2020;20(1):54. doi: 10.1186/s12911-020-1064-9 [published Online First: 2020/03/14]
93. Ose J, Botma A, Balavarca Y, et al. Pathway analysis of genetic variants in folate-mediated one-carbon metabolism-related genes and survival in a prospectively followed cohort of colorectal cancer patients. *Cancer Med* 2018 doi: 10.1002/cam4.1407 [published Online First: 2018/05/31]
94. Perry CA, Renna SA, Khitun E, et al. Ethnicity and race influence the folate status response to controlled folate intakes in young women. *The Journal of nutrition* 2004;134(7):1786-92. doi: 10.1093/jn/134.7.1786 [published Online First: 2004/07/01]
95. Nohr EA, Liew Z. How to investigate and adjust for selection bias in cohort studies. *Acta Obstet Gynecol Scand* 2018;97(4):407-16. doi: 10.1111/aogs.13319 [published Online First: 2018/02/08]

For peer review only

Figure legends

Figure 1 FOCUS Consortium design

For peer review only

For peer review only - http://bmjopen.bmj.com/site/about/guidelines.xhtml

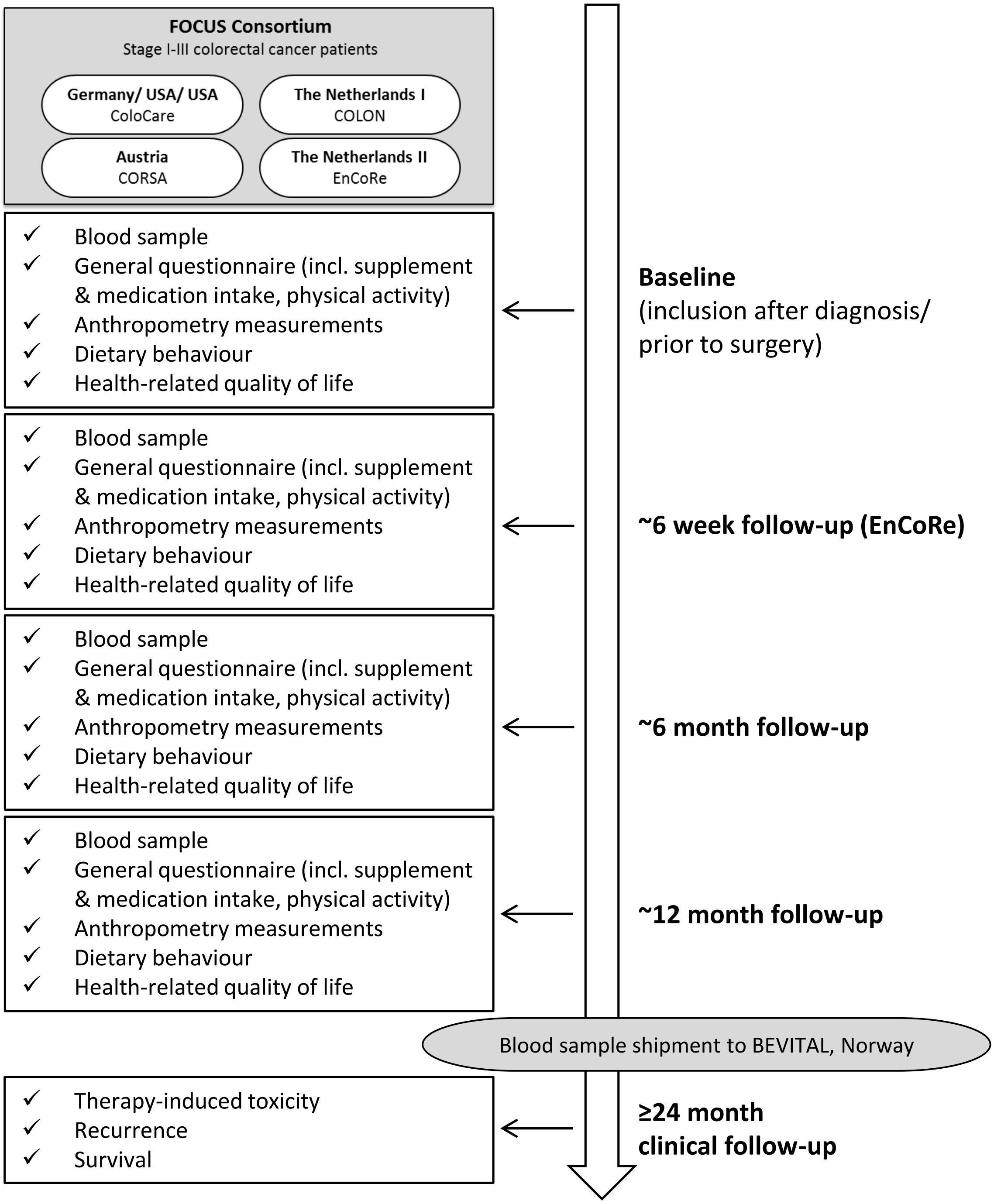
FOCUS Consortium
Stage I-III colorectal cancer patients
Germany/ USA/ USA
ColoCare
The Netherlands I
COLON
Austria
CORSA
The Netherlands II
EnCoRe
✓ Blood sample
✓ General questionnaire (incl. supplement & medication intake, physical activity)
✓ Anthropometry measurements
✓ Dietary behaviour
✓ Health-related quality of life
Baseline
(inclusion after diagnosis/ prior to surgery)
✓ Blood sample
✓ General questionnaire (incl. supplement & medication intake, physical activity)
✓ Anthropometry measurements
✓ Dietary behaviour
✓ Health-related quality of life
~6 week follow-up (EnCoRe)
✓ Blood sample
✓ General questionnaire (incl. supplement & medication intake, physical activity)
✓ Anthropometry measurements
✓ Dietary behaviour
✓ Health-related quality of life
~6 month follow-up
✓ Blood sample
✓ General questionnaire (incl. supplement & medication intake, physical activity)
✓ Anthropometry measurements
✓ Dietary behaviour
✓ Health-related quality of life
~12 month follow-up
Blood sample shipment to BEVITAL, Norway
✓ Therapy-induced toxicity
✓ Recurrence
✓ Survival
≥24 month
clinical follow-up
